# Global identification, structural analysis and expression characterization of cytochrome P450 monooxygenase superfamily in rice

**DOI:** 10.1186/s12864-017-4425-8

**Published:** 2018-01-10

**Authors:** Kaifa Wei, Huiqin Chen

**Affiliations:** School of Biological Sciences and Biotechnology, Minnan Normal University, 36 Xian-Qian-Zhi Street, Zhangzhou, Fujian 363000 China

**Keywords:** Cytochrome P450, Rice, Gene duplication, Expression profile, Development, Drought stress

## Abstract

**Background:**

The cytochrome P450 monooxygenases (CYP450, CYP, P450) catalyze numerous monooxygenation/hydroxylation reactions in biochemical pathways. Although CYP superfamily has been systematically studied in a few species, the genome-scale research about it in rice has not been done.

**Results:**

In this study, a total of 355 CYPs encoded by 326 genes were identified in *japonica* genome. The *OsCYP* genes are classified into 10 clans including 45 families according to phylogenetic analysis. More than half of the genes are distributed in 53 tandem duplicated gene clusters. Intron-exon structure of *OsCYP*s exhibits highly conserved and specificity within a family, and divergences of duplicate genes in gene structure result in non-functionalization, neo-functionalization or sub-functionalization. Selection pressure analysis showed that rice *CYP*s are under purifying selection. The microarray data analysis shows that some genes are tissue-specific expression, such as *OsCYP710A5* and *OsCYP71X14* in endosperm, *OsCYP99A3* and *OsCYP78A16* in root and *OsCYP93G2* and *OsCYP97D7* in leaf. Analysis of RNA-seq data derived from rice leaf developmental gradient indicates that some *OsCYP*s exhibit zone-specific expression patterns. *OsCYP87C2, OsCYP96B5*, *OsCYP96B8* and *OsCYP84A5* were specifically expressed in leaf base and transitional zone. The transcripts of lineages II and IV-1 members were highly abundant in maturing zone. Eighty three *OsCYP*s are differentially expressed in response to drought stress, of which *OsCYP51G3*, *OsCYP709C9*, *OsCYP709C5*, *OsCYP81A6*, *OsCYP72A18* and *OsCYP704A5* are strongly induced and *OsCYP78A16*, *OsCYP89C9* and *OsCYP704A5* are down-regulated significantly, and some of the results were validated by qPCR. And 23 up-regulated and 17 down-regulated genes are specific to *Osbhlh148* mutation under drought stress. Compared to those in wild type, the changes in transcript levels of several genes are slight in the mutant, such as *OsCYP51G3*, *OsCYP94C2*, *OsCYP709C9* and *OsCYP709C5*.

**Conclusion:**

The whole-genomic analysis of rice P450 superfamily provides a clue to understanding biological function of *OsCYP*s in development regulation and drought stress response, and is helpful to rice molecular breeding.

**Electronic supplementary material:**

The online version of this article (10.1186/s12864-017-4425-8) contains supplementary material, which is available to authorized users.

## Background

Cytochromes P450 (CYPs) constitute one of the largest family of enzymatic proteins, whose functions span the synthetic gamut from critical structural components to signaling molecules and defense compounds. In 1958, the first CYP was reported in rat liver microsomes, and then the first plant CYP was identified in cotton in 1969 [1]. Up to now, at least 5100 sequences of plant CYPs have been annotated and named, and most of them were uploaded into a periodically updated website Cytochrome P450 Homepage (http://drnelson.uthsc.edu/CytochromeP450.html). In plants, CYPs play crucial roles in developmental and physiological processes. Arabidopsis CYP715 functions as a key regulator in flower maturation, synchronizing petal expansion and volatile emission [2]. CYP704, CYP703 and CYP86 are required for anther cutin biosynthesis and pollen exine formation [3]. Overexpression of Arabidopsis *CYP78A9* results in large and seedless fruit and the loss-of-function of CYP78B5 in *japonica* leads to giant embryos [4, 5]. CYP73, CYP98, CYP84 and other families catalyze the synthetic reactions in phenylpropanoid pathway, providing a myriad of phenolic compounds functioning as structural components (lignin and suberin), UV protectants (flavonoids), antioxidants (polyphenols), antimicrobials (coumarins, lignans, isoflavonoids) [6]. CYPs are also involved in stress responses, such as drought, salinity, chemical toxicity, oxidative stress and pest infestation. *OsABA8ox3* RNAi lines confers drought tolerance to rice [7]. *Cyp96b4*/*dss1* mutant enhances the accumulation of abscisic acid (ABA) and ABA metabolites in response to drought stress [8]. Besides, rice CYP96B4 may be involved in lipid metabolism and secondary cell wall formation [9, 10]. Arabidopsis *CYP709B1*, *CYP709B2* and *CYP709B3* are induced by salt stress [11]. Tobacco CYP71A10 acting as N-demethylase and ring-methyl hydroxylase is capable of metabolizing phenylurea herbicides [12]. Maize CYP71C1 and CYP71C3 are involved in biosynthesis of DIMBOA which functions as natural defense against damaging pests, fungi and bacteria [13]. Although quite a few rice CYPs have been functionally characterized, the functions of majority of CYP members remain unknown.

The subcellular location of proteins is desirable to explore their biological functions. Almost all of identified plant CYPs are membrane-localized, and most of them are predictably retained in the endoplasmic reticulum (ER) by N-terminal transmembrane helix [14], while CYP74s and CYP97s are located on chloroplast membrane [15, 16]. It is common that animal CYPs are anchored on mitochondria, but there is no report about plant CYP mitochondrial location. Structurally, only a few motifs display high conservation in CYP amino acid sequences: the heme-binding motif FXXGXRXCXG (CXG motif) in C-term, the EXXR motif in the K-helix, the PXRX motif and the AGxD/ET motif associated oxygen binding and activation in the I-helix. Generally, the E-R-R triad (EXXR and PXRX) is required for locking the heme pockets into position and assures the stabilization of core structure. However, the CYP74 family members are specifically modified by the insertion of nine residues into the middle of the heme-binding loop and the lack of AGxD/ET motif. The crystal structures of a variety of bacterial and mammalian P450s have been solved, while most characterized plant P450s are membrane-bound proteins and resistant to standard X-ray and NMR structure determinations. Homology modeling is used as a reliable and relatively rapid alternative method for analyzing structure-function relationships and predicting substrates. The overall folds of CYPs are relatively conserved, however, some regions have the most variability in length and backbone conformation, particularly substrate-recognition regions [17].

In Arabidopsis, 246 full-length P450 genes were identified, and at least fifty CYPs have been functionally determined so far [18]. Recently, a large-scale co-expression analysis with functional annotation was performed to predict Arabidopsis *CYP* functions [19]. Although the evolutionary relationships of partial *japonica* and *indica CYP* genes obtained from GeneBank and Beijing Genomics Institute were discussed previously [18], the genome-wide identification, structural analysis and expression characterization of P450 superfamily have not been conducted based on new rice pseudomolecules and annotation released in 2011. In this study, we focused on the evolutionary, structural and expressional analysis of P450s in rice. It is helpful to further explore the functions of rice CYPs, elucidate the molecular mechanism of development and drought responses, and develop improved varieties.

## Results

### Identification and analyses of full-length OsCYP protein sequences

After checking conserved domain, 472 protein sequences were identified, whereas 117 of them (such as Os01g36350, Os01g43720 and Os01g50520) with incomplete P450 signature motifs were discarded. Eventually, a total of 355 proteins encoded by 326 genes were selected for further analysis. The Cys in CXG motif is strictly conserved across all OsCYPs except for OsCYP71AA2 (Arg-450) and OsCYP71C33 (Val-216). The sequence lengths of the OsCYPs range from 131 to 1933 amino acids (AA), most of which fall in between 450 to 550 AA. Twenty-seven out of 326 *OsCYP*s were predicted to be nearly intact pseudogenes and tagged with a “P” on the end of the CYP name. All genes were assigned to 45 families according to standard nomenclature and classified into A-type (18 families) and Non-A type (27 families) consisting of 185 and 141 genes, respectively. Each of the families CYP703, CYP715, CYP722, CYP724, CYP727, CYP733, CYP85, CYP88 and CYP98 consists of a single gene, while CYP71 with 80 members is the largest family. Of the encoding proteins, 341 OsCYPs were predicted to be located on ER, 11 on chloroplast (OsCYP701As and OsCYP74s) and three on mitochondrion (OsCYP51H7P, OsCYP711A2 and CYP727A1) by LocTree3. All the related information was summarized in Additional file 1: Table S1. In addition to the subcellular localization prediction, conserved motif analysis is one of the important methods for sequence-based protein function prediction. A total of 50 conserved motifs (motifs 1–50) were captured by MEME software (Additional file 2: Figure S1 and Additional file 3: Table S2). Motifs 1, 2, 3 and 5 correspond to motifs CXG, EXXR, PXRX and AGxD/ET, respectively. Several motifs were found in most proteins, such as motif 4 spanning the connection between the β2–2 strand and β1–3 strand, motif 7 immediately C-terminal to the AGxD/ET motif, and motif 9 immediately C-terminal to the CXG motif. While some are present in specific family, such as motif 28 in CYP51, motifs 41 and 48 in CYP710, motif 33 in CYP76 and motif 39 in CYP89.

### Homology modeling of OsCYPCYP51Gs and OsCYP74As

**Fig. 1:**
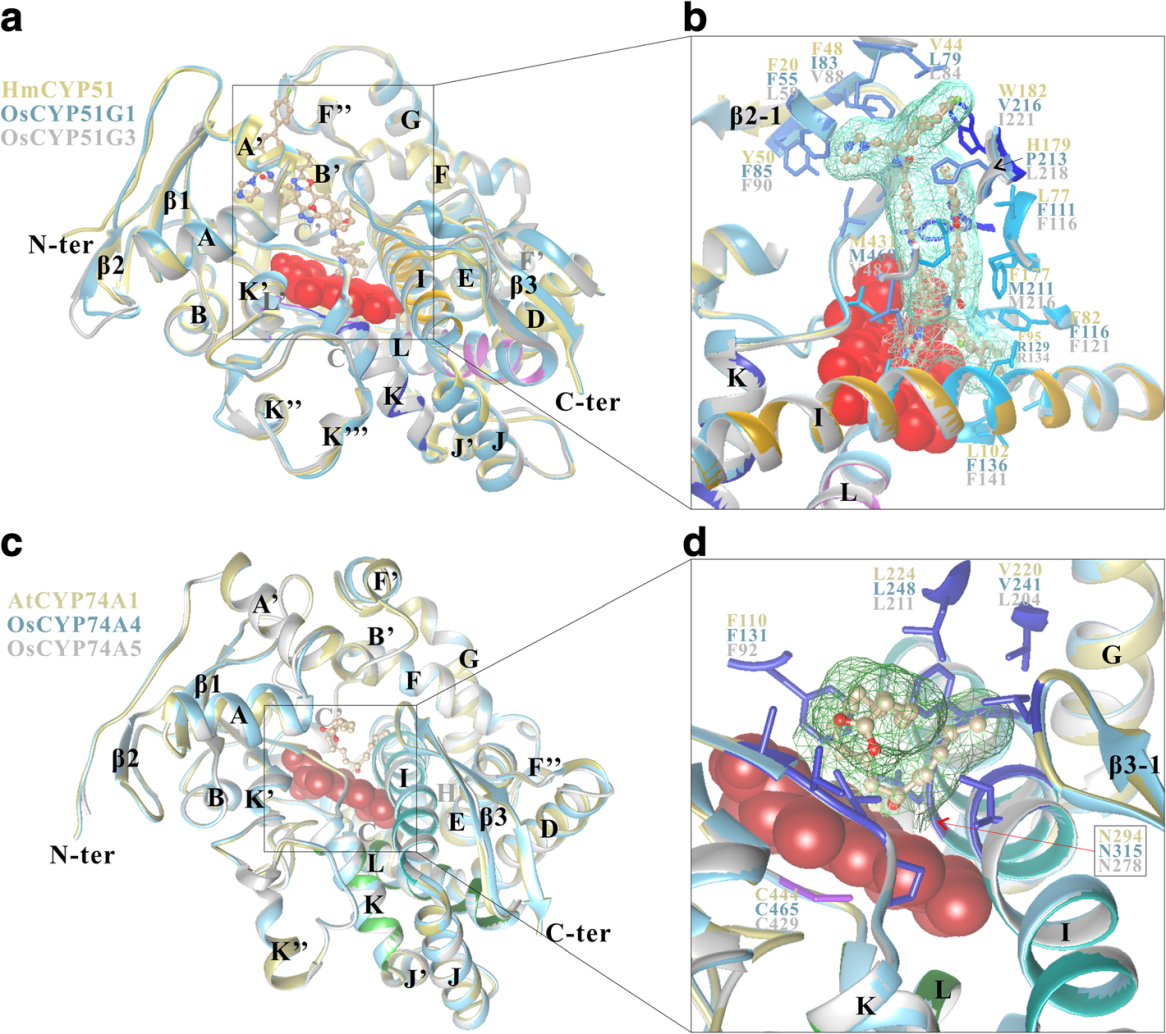
Superposition of three dimensional homology models of rice CYPs and template structures. **a** Structures of HmCYP51 (dark khaki), OsCYP51G1 (sky blue) and OsCYP51G3 (gray). Heme molecule is shown as sphere representation. Two VFV molecules are shown by ball-stick model. **b** Close-up view of the substrate (two VFV molecules surrounded with cyan mesh) environment. The substrate recognition sites were identified based on the alignment with template structure and shown by stick model. **c** Structures of AtCYP74A1 (dark khaki), OsCYP74A4 (sky blue) and OsCYP74A5 (gray). Heme molecule is shown as sphere representation. 13(S)-HPOT molecule is shown by ball-stick model. **d** Close-up view of the substrate (13(S)-HPOT molecule surrounded with forest green mesh) environment. The substrate recognition sites were identified based on the alignment with template structure and shown by stick model

To better understand tertiary structure and substrate recognition, homology models of OsCYP51Gs and OsCYP74As were built based on the best template structure of human CYP51 (PDB: 4UHL) and Arabidopsis CYP74A1 (PDB: 3DSI), respectively. The crystal structure of AtCYP74A1, free and in complexes with substrates or reaction intermediates analogues, have been solved, which is the first 3D structure of plant CYPs [20]. The ERRAT showed above 92 overall quality factor, indicating high quality model built (Additional file 4: Figure S2). As shown in Fig. 1a and c, these structures have 12 α-helices in common (A-L) and three β-sheets (a four-stranded sheet (β1), a three-stranded sheet (β3) and a two-stranded sheet (β2)). The β3–1 of CYP51 is located in DE-loop, while a coil is in that region of CYP74A. The β3–1 of CYP74A is immediately C-terminal to the αL. An insertion of nine residues into the middle of the heme-binding loop and the helix I without AGxD/ET motif are specific to CYP74As. αC, αF, αG, αI, αL, β1, BC-loop, FG-loop and the turn between β3–1 and β3–2 surround the heme and house the residues defining the SRS regions 1–6 (Fig. 1b and d and Additional file 5: Figure S3). Substrate binding residues of the HmCYP51 and AtCYP74A1 were marked respectively with red and blue triangles, and mapped onto the aligned sequences OsCYP51G1, OsCYP51G3, OsCYP71A4 and OsCYP71A5. However, the level of sequence identity among the putative SRSs is relatively low.

### Phylogenetic relationship of *CYPs*

To explore the evolutionary relationships among *OsCYP*s, an unrooted NJ tree of 326 full-length OsCYP sequences was constructed (Additional file 6: Figure S4a). Based on the phylogenetic analysis, *OsCYP*s were classified into 10 clans, including six single-family clans (CYP727, CYP74, CYP710, CYP51, CYP97 and CYP711) and four multi-family clans (CYP85, CYP86, CYP72 and CYP71). The three largest clans (in descending order) are CYP71, CYP86 and CYP72. Further, the ten clans were grouped into four clusters. CYP74 clan is considered as a special one, and CYP51, CYP710 and CYP85 clans were organized into a cluster, of which CYP51 clan is likely to be the oldest. CYP727, CYP72, CYP97 and CYP86 clans were tightly clustered together to form one cluster, and CYP711 and CPY71 clans were gathered in another cluster.

To investigate the occurrence, divergence and evolution of CYP families in rice, a comprehensive comparison among *Oryza sativa*, *Brachypodium distachyon*, *Populus alba*, *Carica papaya*, *Citrus clementina*, *Arabidopsis thaliana*, *Glycine max*, *Salvia miltiorrhiza*, *Nelumbo nucifera* and *Vitis vinifer* was conducted. Some families only occur in dicots, such as CYP716, CYP82 and CYP720, and CYP723 and CYP99 are only present in monocots (Additional file 7: Figure S5a, b which respectively fell inside CYP89 and CYP71 families on phylogenetic tree. Similarly, CYP724 fell inside CYP90 family. It’s worth noting that none of the defined rice CYP families emerged after divergence of rice from the other Gramineae. The gene number-based hierarchical clustering of CYP families of different species showed that the family loss seems to be limited to single taxa or single species. And the gene family expansion can be observed in six families including CYP71, CYP76, CYP94, CYP89, CYP72 and CYP81. Particularly, the members of families CYP94 associated with fatty acid metabolism and CYP71 with diverse functions in rice are more abundant than those in other species.

### Gene structure and duplication events of *OsCYP*s

The intron-exon organization compared with phylogenetic tree information is helpful to better establish evolutionary relatedness and predict gene functions. To understand the gene structural features of *OsCYP*s, intron-exon structure was detected based on their evolutionary relationships (Additional file 6: Figure S4b). Some families consist of intronless genes, such as CYP74, CYP710 and CYP723. CYP96 and CYP94 family members are generally intronless. Most members of CYP85 clan have up to eight or nine introns. CYP72 clan is characterized by four introns with relatively stable position and phase. *OsCYP97C2*, *OsCYP97B4* and *OsCYP97A4* are interrupted by 9, 13 and 14 introns, respectively. One hundred out of 185 genes in CYP71 clan have only a single phase-zero intron, implying their close phylogenetic relationship. Intron positions and phases are conserved in most members of a family, such as CYP701, CYP78, CYP71 families. For some pseudogenes, at least one intron was inserted into nuclear genes, such as *CYP89B7P*, *CYP89B8P* and *CYP89B12P*. Compared with *CYP72A32* and *CYP72A33*, *CYP72A31P* lost a region that corresponds to the first and second exons. In fact, the intron loss and gain occurred frequently during the evolutionary process, which may result in non-functionalization, neo-functionalization or sub-functionalization.

**Fig. 2:**
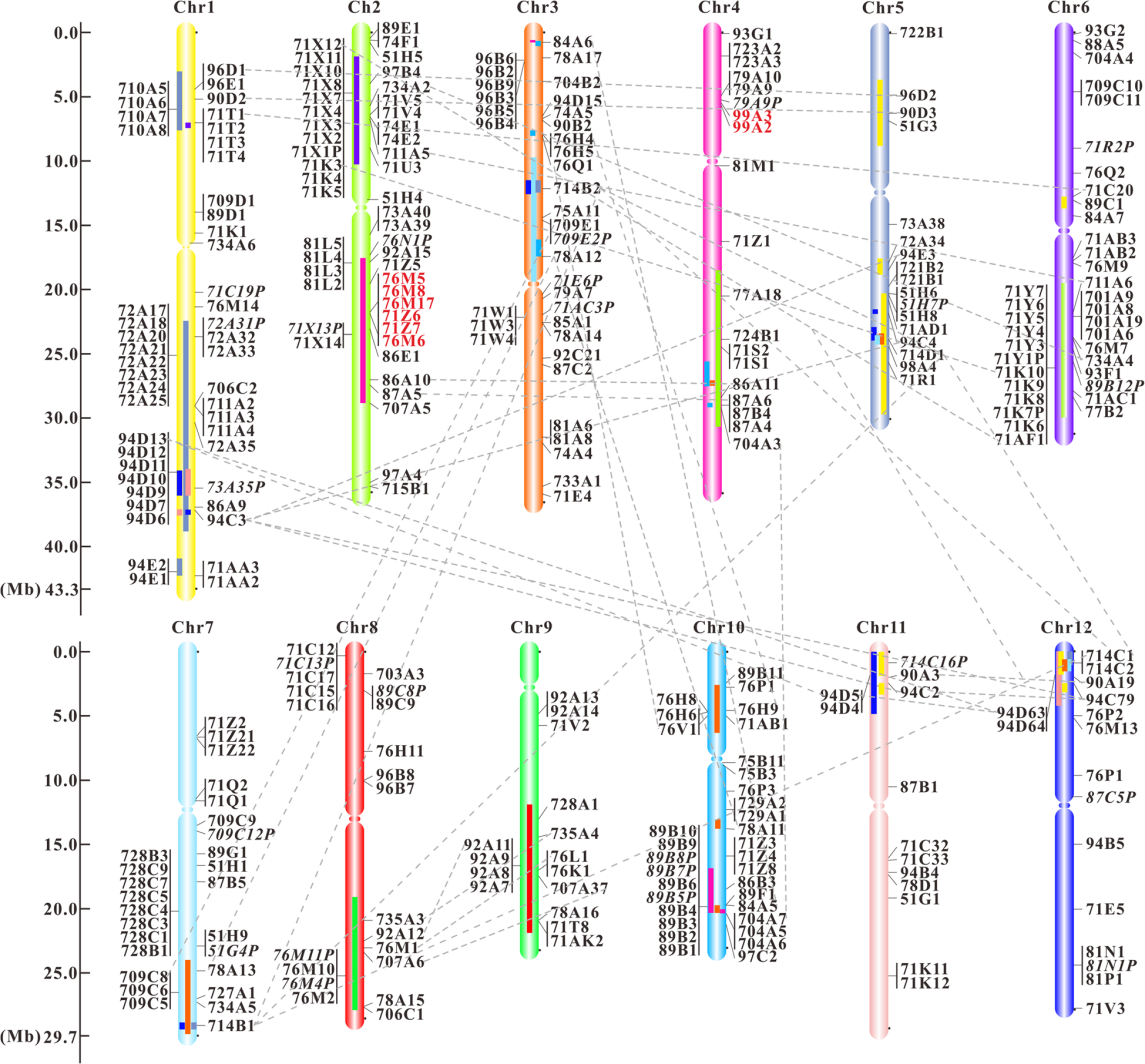
Chromosomal localization of *OsCYP* genes. All putative *OsCYP*s are shown on the chromosomes and indicated by CYP names omitting “CYP” root. The scale is in megabases (Mb). Chromosome numbers are indicated at the top of each bar, and ellipse on each chromosome shows the rough position of centromere. The rectangle filled with the colour of corresponding chromosome is superposed on chromosome to show the duplicated chromosomal segment. The segmentally duplicated genes are connected by gray dashed lines, and tandemly duplicated genes are joined with black vertical lines. The genes within metabolic gene clusters are marked with red characters

To further study evolutionary mechanism of rice CYP superfamily, both tandem and segmental duplication events were analyzed. As illustrated in Fig. 2, the relatively high gene densities were observed in some regions of the long arms of chromosomes 1, 3, 7, and 10, and of the short arms of chromosomes 2 and 6. More than half of the genes were found in 53 tandem duplicated clusters, 16 out of which contain more than three members. The two largest clusters (*CYP71X-K* and *CYP71Y-K-AF*) respectively composed of 12 members are on chromosomes 2p and 6p, and the second largest cluster consisting of 10 *CYP89*s is on chromosome 10q. The segmental duplication blocks were highlighted on corresponding chromosomal regions in genetic linkage maps. The results showed that 55 *OsCYP*s (16.87%) are involved in segmental duplication. Unlike tandem duplication, the collinear paired *CYP*s tend to belong to different subfamilies. For instance, *OsCYP714D1* on chromosome 5 is linked to *OsCYP714B1* on chromosome 7 and *OsCYP714C1* on chromosome 12.

### Selection pressure estimation

Many of the unstable CYP genes may be subject to natural selection during the evolution, and the location of positively selected residues is important for inferring gene function. Some positively selected sites were estimated from nine data sets by PAML and DATAMONKEY server, the results were listed in Additional file 8: Table S3, Additional file 9: Table S4, Additional file 10: Table S5, Additional file 11: Table S6, Additional file 12: Table S7 and Additional file 13: Table S8. Through integrative analysis, six positive selection sites were determined, one in CYP71 clan, one in CYP85 clan, one in CYP86 clan, and three in CYP710 clan. The results showed that positive selection in amino acid sequence is restricted to the unstable *CYP* genes. Recent study suggested that sites under positive selection in CYPs are more likely to be associated with substrate-binding or substrate-channel regions [21]. However, the sites detected in our analysis do not locate in those regions. It is clearly that the vast majority of *OsCYP*s are under strong purifying selection. The ratios of dN/dS for 230 homologous gene pairs were calculated, most of which centre around 0.35 (Additional file 14: Figure S6). The overall low ω values of the pairs indicated that the retained duplicates are evolving under purifying selection. Only five gene pairs (*CYP71K9*/*CYP71Y5, CYP71T3*/*CYP71T4*, *CYP71Z1*/*CYP71Z8*, *CYP81N1*/*CYP81NP* and *CYP709C10*/*CYP709C11*) have ω values of >1. Although successive gene duplication events resulted in the expansion of CYP71 clan, a few genes are under positive selection. As we expected, single-family clans are under purifying selection, while multiple-family clans tend to be under positive selection, which is associated with promoting sub/neo-functionalization.

### *Cis*-regulatory elements and potential miRNA targets

*Cis-regulatory* elements function as binding sites for transcription factor to control gene expression involved in the regulation of development and stress responses. A total of 59 putative *cis*-regulatory elements were found to be presented in 326 *OsCYP*s (Additional file 15: Table S9). Further analysis showed that hormone-response elements, e.g. ARE-2, CAAT-box, P-box, ABRE, DRE/CRT, GCC-box and as-1, are presented in most of the promoter regions of *OsCYP*s. Among them, ABRE and DRE/CRT elements that are related with salt, drought and cold stress responses are respectively found in 196 (60.1%) and 144 (44.2%) *OsCYP*s. A number of *OsCYP*s contain organogenesis-related elements, such as GCN4 motif or/and AACA motif in 109 *OsCYP*s for endosperm-specific gene expression, RY/Sph motif in 181 genes (e.g. *OsCYP85A1*, *OsCYP73A39* and *OsCYP86A11*) and SEF4 motif in ten genes (e.g. *OsCYP710A5*, *OsCYP709C9* and *OsCYP89C9*) for seed-specific expression.

MiRNAs play important roles in negatively regulating specific target mRNAs at posttranscriptional levels through transcript cleavage or repressing translation. A total of 452 miRNA-target pairs referring 474 target sites were obtained (Additional file 16: Table S10). Previous research reported that *OsCYP51G3* expression can be posttranscriptionally regulated by osa-miR1848 in developing organs and response to salt stress [22]. In this analysis, osa-miR1848 target *OsCYP51G3* mRNA with a score of 86. Of these predicted miRNA-target pairs, 33 pairs have a score of more than 86. Among them the osa-miR2927-*OsCYP94C3* pair has the highest score. High-throughput sequencing of miRNAs showed that 14 rice miRNA families (osa-miR156, miR160, miR164, miR166, miR167, miR168, miR171, miR319, miR396, miR397, miR408, miR528, miR530, miR820) were significantly down-regulated after drought treatment [23]. We found that 38 candidate mRNAs corresponding to 30 *OsCYP* genes may be targeted by these miRNAs (Additional file 16: Table S10), such as *OsCYP707A5*, *OsCYP709C5*, *OsCYP709C9*, *OsCYP71P1* and *OsCYP71X7*. On the contrary, Osa-miR1436 was abundantly expressed under drought stress [23], which were predicted to regulate the abundance of *OsCYP704A5* and *OsCYP89C9* transcripts with scores of 96 and 85 respectively.

### Spatial and temporal expression of *OsCYP*s during rice development

**Fig. 3:**
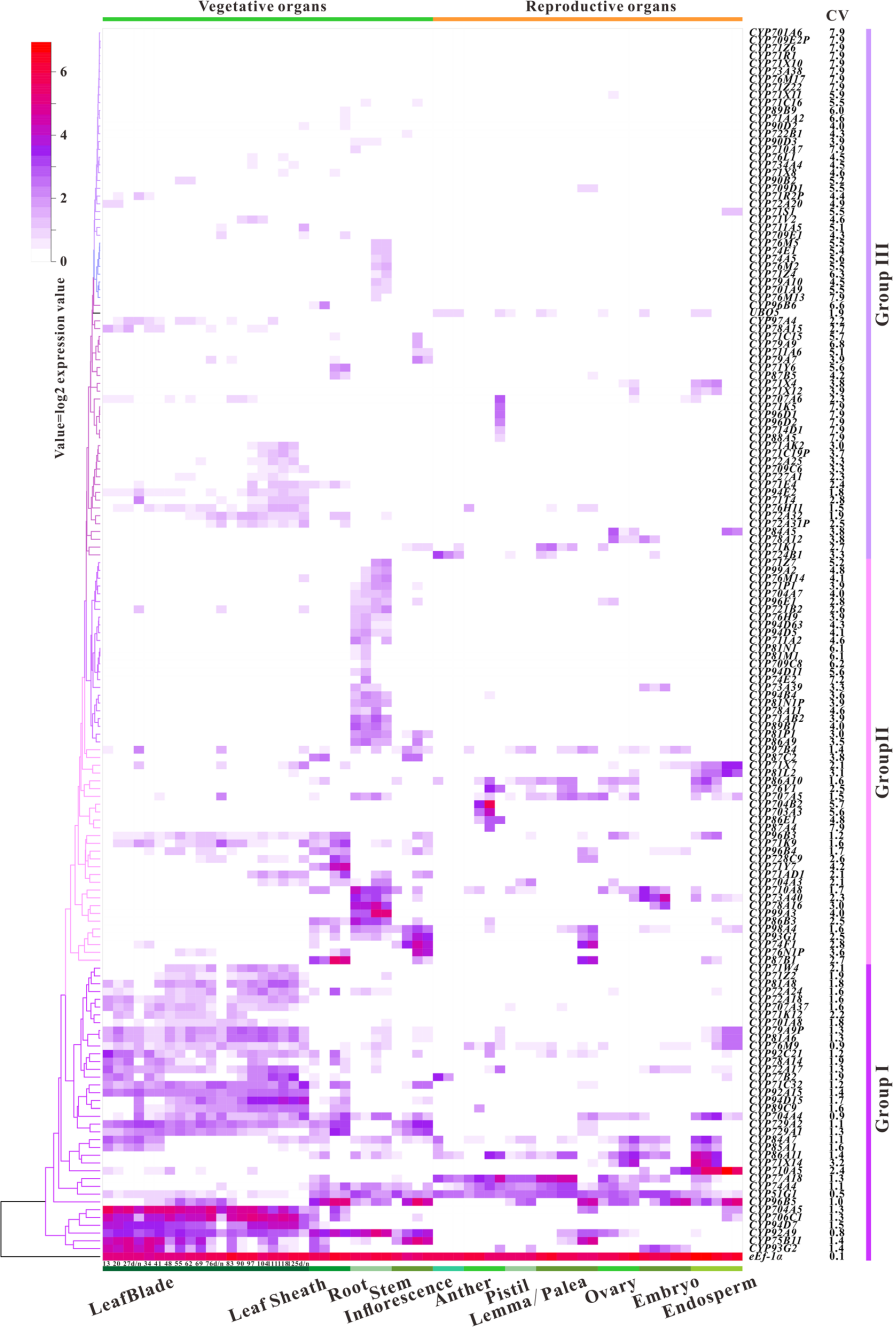
The expression profiling of *OsCYP*s throughout life cycle of rice grown. Hierarchial clustering heatmap displays expression patterns of 156 *OsCYP* genes in 62 distinct tissues representing 12 major organ systems in Nipponbare, which are divided into three groups. The CV values are added on the right side of CYP labels

**Fig. 4:**
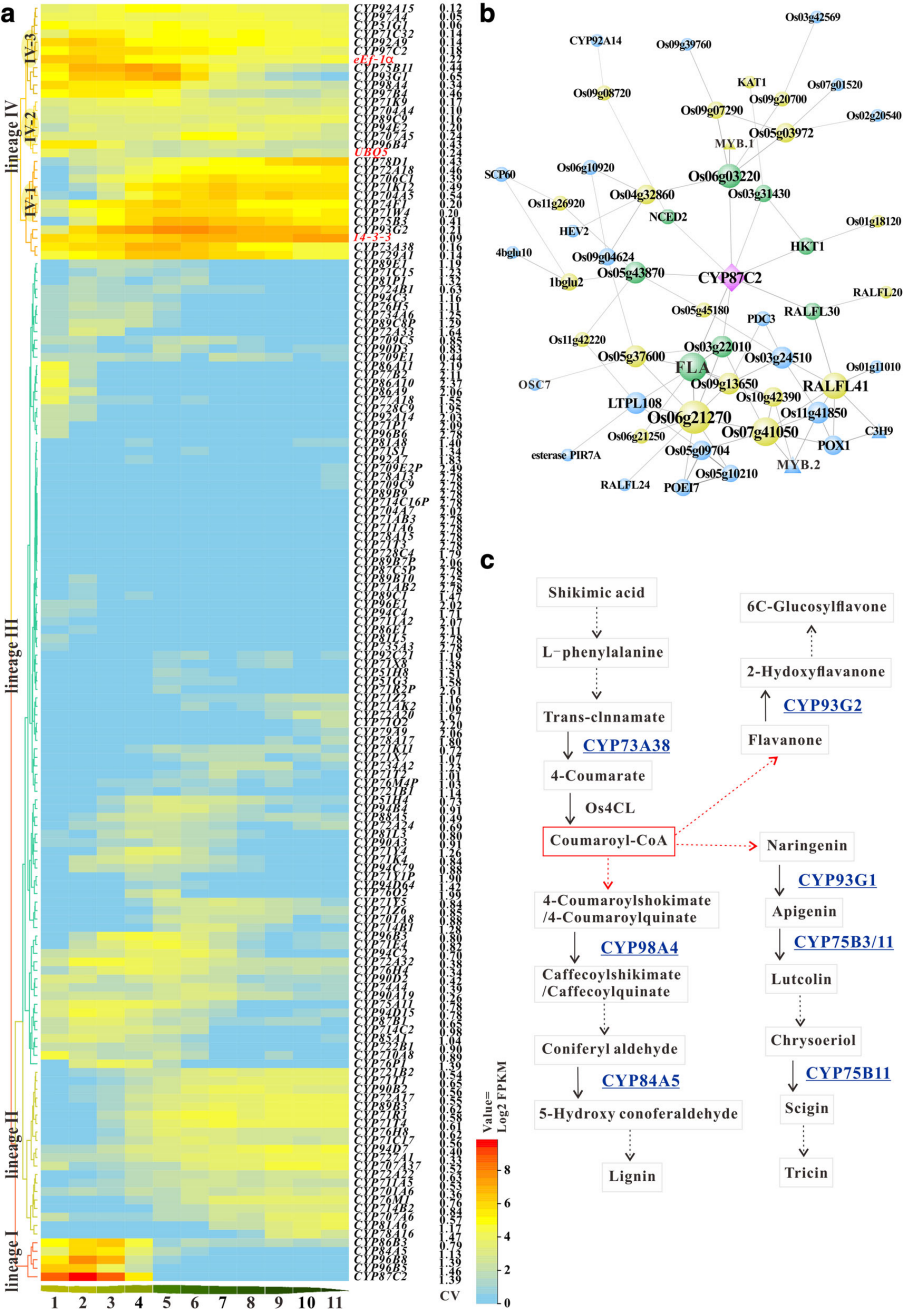
Spatially and temporally specific expression of *OsCYP*s in leaf. **a** Hierarchical clustering shows the similar and distinct expression patterns of 148 *OsCYP* genes in 11 continuous leaf sections. The CV values are shown on the right side of the CYP labels. **b** A co-expression network constructed using *OsCYP87C2* as “guide gene” and visualized using Cytoscape software. The three triangles represent for the transcription factor genes. **c** The schematic of phenylpropanoid pathway

As an initial step towards understanding of the biological function, the expression patterns of *OsCYP*s during entire growth and developmental cycle were analyzed using available oligonucleotide microarray data. The log2-transformed expression values of 156 *OsCYP*s were extracted, and the average values were visualized using a hierarchically clustered heatmap. As shown in Fig. 3, these *OsCYP*s were categorized into three groups with different expression patterns, and 121 out of 156 *OsCYP*s were considered as tissue-specific genes. Within group I, 37 genes were expressed at relatively higher levels in either vegetative or reproductive organs, of which 32 exhibited a CV >1. *OsCYP710A5*, *OsCYP71X14* and *OsCYP86A11* were expressed at extremely high levels in endosperm. The relatively high mRNA levels of *OsCYP77A18* were detected in inflorescence, anther, pistil, lemma and palea. Additionally, *OsCYP96B5* was expressed at high levels in leaf sheath, stem, endosperm (from 28 and 42 days after flowering), embryo (from 28 and 42 days after flowering), lemma (from >7.0 mm floret) and palea (from >7.0 mm floret). Most of the group members were relatively highly expressed in leaf blade at different development stages, such as *OsCYP704A5*, *OsCYP706C1*, *OsCYP94D7*, *OsCYP92A9*, *OsCYP93G2* and *OsCYP75B11*. Further, the RNA-seq data were used for analyzing expression patterns of *OsCYP450* genes along leaf developmental gradient. As shown in Fig. 4a, 148 *OsCYP*s with FPKM ≥1 in one or more leaf tissues were categorized into four lineages. In lineage I, *OsCYP87C2*, Os*CYP96B5*, *OsCYP96B8*, *OsCYP84A5* and *OsCYP86B3* transcripts were at high abundances from 6.3 to 481 FPKM in leaf base (sections 1–2, sink tissue) and transition zone (sections 3–4, undergoing the sink-source transition), and then reduced to extremely low levels. The expressions of genes in lineage II were gradually increased with leaf maturing, and the CV values of genes are lower than 1 except for those of *OsCYP81A6* and *OsCYP78A16*. Lineage III contains 95 genes with relatively low expression levels, 65 of which have CV of >1. Among them, *OsCYP86A11*, *OsCYP77B2*, *OsCYP86A10*, *OsCYP86A9*, *OsCYP728C9* and *OsCYP92A14* were specifically expressed in leaf base (sections 1–2). Several genes in lineage IV maintained high expression levels in sections 1 to 6, such as *OsCYP75B11 OsCYP93G1*, *OsCYP97C2* and *OsCYP92A9*. Obviously, *OsCYP75B3*, *OsCYP93G2*, *OsCYP73A38*, *OsCYP729A1*, *OsCYP704A5* and *OsCYP706C1* were highly expressed at sections 3 to 11. Oligonucleotide microarray data analysis showed that a majority of genes in group II showed root-specific expression, such as *OsCYP99A3*, *OsCYP78A16, OsCYP710A8*, *OsCYP86B3*, *OsCYP89B1* and *OsCYP71AB2*. And some genes were expressed in particular tissues, such as *OsCYP704B2*, *OsCYP703A3*, *OsCYP86B3* and *OsCYP87A4* in anther, *OsCYP71Y7* in leaf sheath, *OsCYP76N1P* in stem, *OsCYP81L2* in endosperm, *OsCYP74F1* in stem lemma and palea, and *OsCYP86B1* in leaf sheath lemma and palea. The vast majority of group III members were expressed at low levels, which might be involved in other functions like stress management.

### RNA-seq-based transcriptome profiling of *OsCYP*s under drought treatment

**Fig. 5:**
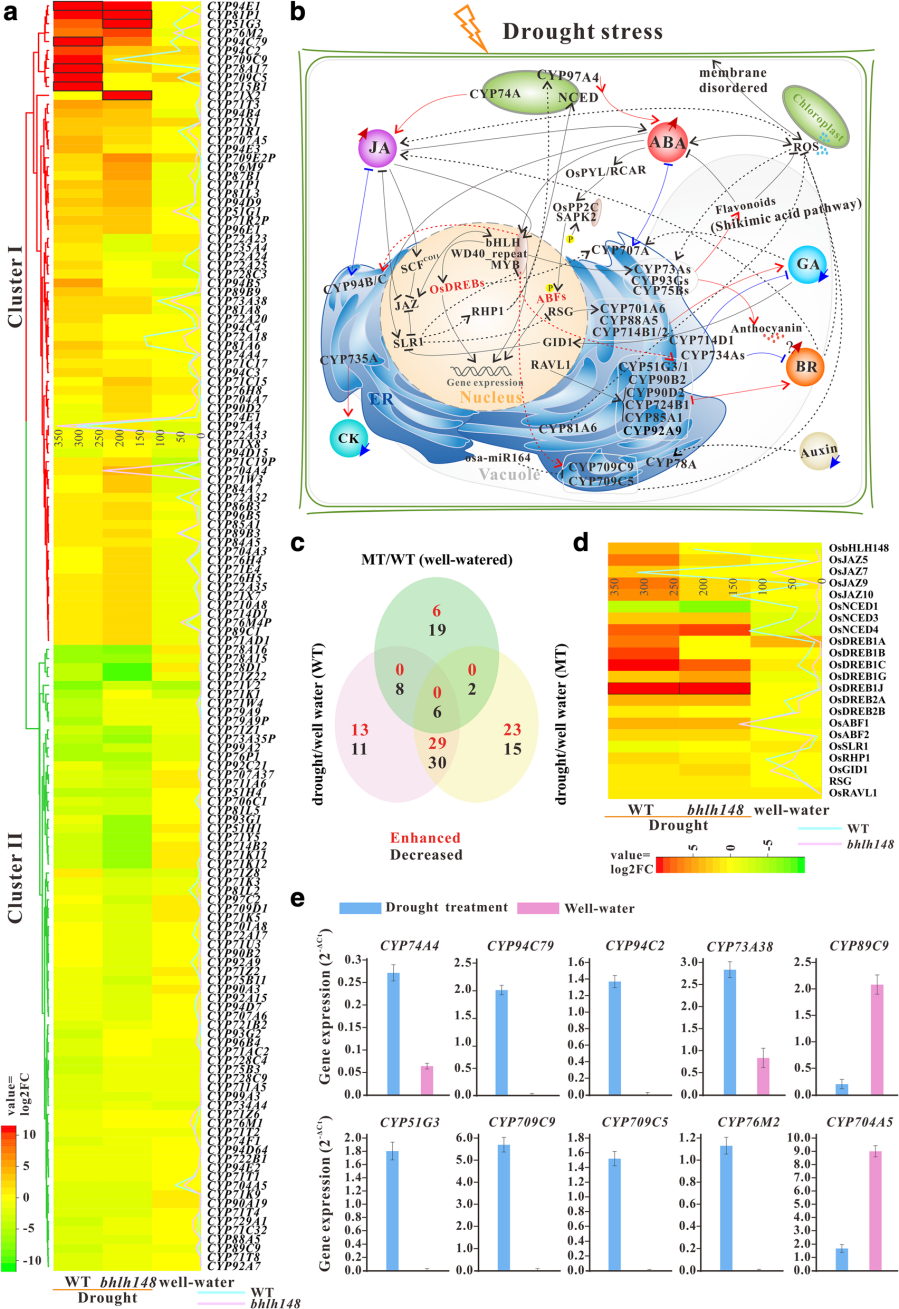
Differential expression of *OsCYP* genes and the drought-responsive network. **a** Expression profiles of 142 *OsCYP* genes in WT and *Osbhlh148* mutant under drought conditions. The line charts show the dynamic changes of gene expression levels in drought stress response. The genes with no expression values in well-watered condition are presented by black boxes. **b** Overveiw of drought-responsive network. The red solid line depict synthetic route; the blue solid line depict catabolic route. **c** Venn diagram showing the number of transcripts enhanced or decreased in response to drought stress. **d** Expression profiles of 22 candidate genes in drought stress signal pathway. **e** The expression levels of 10 representative drought-responsive *OsCYP*s were determined by qPCR

A total of 142 differentially expressed *OsCYP*s were identified in response to drought stress. They were grouped into two clusters according to the hierarchical clustering analysis of log2 FC values, as can be seen in Fig. 5a. Many cluster I genes were up-regulated, yet most genes in cluster II were down-regulated. Of the differentially expressed genes, 42 up-regulated and 55 down-regulated genes were responsive to drought stress in wild type (WT) rice. The mRNA levels of *OsCYP76M2*, *OsCYP51G3*, *OsCYP94C2*, *OsCYP709C9* and *OsCYP94C79* were increased by 246.84- up to 3222.80-fold. *OsCYP97A4*, *OsCYP78A16*, *OsCYP78D1*, *OsCYP71K12* and *OsCYP704A5* expressions were decreased by 1.73- to 113.10-fold. To confirm the expression patterns of *OsCYP*s, *Oryza sativa* L.ssp. *japonica* cv. Nipponbare was treated by drought-stress, and some results obtained from the RNA-seq data analysis were confirmed by qPCR analysis (Fig. 5e). The primers for qPCR were shown in Additional file 17: Table S11. Mutation of *Osbhlh148* resulted in significant enhancement of six genes expressions (*OsCYP735A4*, *OsCYP78A16*, *OsCYP707A37*, *OsCYP714B2*, *OsCYP97C2* and *OsCYP71K5*) and suppression of 35 genes transcripts (such as *OsCYP97A4*, *OsCYP704A4* and *OsCYP71W4*) in irrigation condition. Under drought stress, 53 and 52 genes were up-regulated and down-regulated in *Osbhlh148* mutant type (MT), respectively. The Venn diagram shows the overlap of up- or down-regulated DEGs under three different experimental conditions (Fig. 5c). Of the DEGs under drought treatment, only 13 upregulated and 19 downregulated genes are specific to WT, while 23 upregulated and 17 downregulated genes are MT-specific. The two genotypes have 29 induced genes (such as *OsCYP51G3*, *OsCYP76M2*, *OsCYP73A38*, *OsCYP709C9* and *OsCYP94C2*) and 36 repressed genes (e.g. *OsCYP78D1*, *OsCYP71K12*, *OsCYP707A37*, *OsCYP704A5* and *OsCYP89C9*) in common after water-deficit treatment. When compared with WT, the loss-of-function of *OsbHLH148* led to transcription decrease of some genes under drought stress, particularly, *OsCYP709C9*, *OsCYP709C5*, *OsCYP81P1*, *OsCYP51G3*, *OsCYP94C2*, *OsCYP94C79*, *OsCYP72A18* and *OsCYP81A6*. Interestingly, *OsCYP51G1* homologous to *OsCYP51G3* was expressed at higher levels in MT than in WT under drought stress.

## Discussion

### Substrate-dependent evolution of *CYP* genes

In this study, a comprehensive analysis of evolutionary relationship, regulation and expression of P450 genes in rice was conducted. The CYP450 superfamily is larger in rice (326 genes) than in Arabidopsis (272 genes). A total of 326 *OsCYP*s were classified into 45 families. In contrast, the Arabidopsis *CYP*s were assigned into 47 families, of which nine (CYP716, CYP720, CYP718, CYP708, CYP702, CYP705, CYP83, CYP712 and CYP82) were not found in rice. Seven families CYP728, CYP729, CYP727, CYP733, CYP99, CYP723 and CYP92 were not present in Arabidopsis. In this evolutionary analysis, *CYP*s were clustered into 10 clans and were further grouped into four clusters with distinct functions. *CYP*s fell into one clan likely diverged from a common ancestor gene. CYP74 clan which relies on neither molecular oxygen nor NADPH to catalyze internal oxygen transfer was likely to have occurred in early photosynthetic organisms when O_2_ pressure was still low on the Earth [24]. CYP51, CYP710 and CYP85 clans were clustered together on the tree, of which CYP51 is the oldest; the sector may have evolved from a sterol metabolizing CYP51 ancestor. CYP710s act after CYP51s in sterol biosynthesis, and the CYP85 clan members may participate in terpenoid-related pathway, brassinolide (BR) biosynthesis and ABA catabolism [25]. We speculated that the three clans CYP72, CYP97 and CYP86 may share a common ancestor to CYP727. Although CYP727 family does not exist in moss, moss-specific family CYP751 may be a member of CYP727 clan and a long diverging CYP727 ortholog [26]. CYP72 clan is associated with the metabolism of fatty acids, isoprenoids and hormones (BRs and GAs) and the biosynthesis of cytokinins (CKs) [27–30]. OsCYP97A4 catalyzes ring-hydroxylation of α- and β-carotenes to form essential components of the light-harvesting systems and zeaxanthin [31]. And some members of CYP86 clan are able to hydroxylate and epoxidate fatty acids, fatty alcohols or alkanes and their derivatives [32, 33]. CYP711 and CPY71 may have a common origin, and CYP711s catalyze different critical steps in the synthesis of carotenoid-derived hormones strigolactones (SLs) [34]. The largest clan CYP71 composed of 17 families represents a huge diversity of functions, involved in the metabolism of aromatic and aliphatic amino acid derivatives, small isoprenoids and some triterpenoid derivatives, alkaloids, fatty acids and precursors of hormones [35–37]. These results provide powerful evidence that the duplicates diverge to acquire new functions. Additionally, CYP99, CYP723 and CYP724 families respectively fell inside the CYP71, CYP89 and CYP90 families on tree, illustrating to the phenomenon of family creep that stretches the 40% boundary backwards. OsCYP724B1 and OsCYP90B2 have similar gene structure and amino acid patterns, but are not duplicates, which play important roles in BR biosynthesis [38]. Similarly, these characteristics can be found in CYP99s and CYP723s. Rapid evolution of P450s has conferred on plants the capacity to adapt to the external environments, and one of the driving forces behind the diversification of *CYP*s is the so-called chemical war between plants and other organizations. Reaction types are so varied and substrates so diverse and numerous, but most of substrates of CYPs remain unidentified.

### Roles of *OsCYP*s in specific organ and development stage

CYPs are involved in a variety of biochemical pathways participating in the developmental processes. We try to explore the functions of *OsCYP*s during the processes through microarray dataset. Some genes were highly expressed in particular reproductive organ, such as *OsCYP704B2*, *OsCYP703A3*, *OsCYP86E1* and *OsCYP87A4* in anther, *OsCYP710A5*, *OsCYP71X14* and *OsCYP86A11* in endosperm. Multiple lines of evidence suggest that AtCYP704B1, AtCYP703A2, AtCYP86C3, OsCYP704B2 and OsCYP703A3 catalyze the hydroxylation of mid-chain and long-chain fatty acids during pollen exine biosynthesis [39, 40]. OsCYP86E1 shares 50% amino-acid identity to AtCYP86C1, suggesting a close evolutionary and functional relationship between *OsCYP86Es* and *AtCYP86Cs*. *OsCYP87A6* (old name: *OsCYP87A3*), a homologue of *OsCYP87A4*, is a primary auxin response gene and in turn affects local auxin levels that is critical for pollen development [41, 42]. The expressions of *OsCYP704B2* and *OsCYP703A3* can be co-regulated by TDR (OsbHLH005) which recognized their E-box [3]. We noted that E-box element also existed in the promoter of anther-specific gene *OsCYP87A4.* Of the highly expressed genes in the endosperm, *OsCYP710A5* may be associated with the sterols accumulation in grain. OsCYP86A11 has 85% sequence identity to AtCYP86A8 that function as fatty acid ω-hydroxylase involved in cutin biosyntheis [43]. In grass seed, semipermeable layer consisting of cutin or suberin membrane restricts solute transport through the seed coat. OsCYP77A18 expressed in multiple organs and development stages is homologous with AtCYP77A6 and AtCYP77A4 which function downstream of AtCYP86As [44]. Recent study reported that *OsCYP714D1* (*EUI1*) overexpression resulted in GA-deficient phenotypes, with shortened internodes and poor panicle exsertion [45]. In our analysis, this gene with weak expression was detected in anther. Most *OsCYP* genes were highly expressed in vegetative organs, such as *OsCYP99A3*, *OsCYP78A16*, *OsCYP710A8* and *OsCYP73A40* in root, *OsCYP76N1P* and *OsCYP74F1* in stem, *OsCYP87B1* and *OsCYP71Y7* in leaf sheath, *OsCYP704A5*, *OsCYP706C1*, *OsCYP94D7*, *OsCYP93G2* and *OsCYP75B11* in leaf blade. *OsCYP99A3* and *OsCYP99A2* encoding multifunctional diterpene oxidases participated in the momilactone diterpenoids biosynthesis in root [46], which may contribute to maintain the stability of the rhizosphere soil microorganism community. *OsCYP78A16* is homologous to *OsCYP78A13* and *GmCYP78A10* that were related to the regulation of organ size and cell proliferation [47, 48]. *OsCYP74F1* (*OsHPL3*) was expressed exclusively in leaf after brown planthopper or striped stem borer infestation [49], while we found that is also expressed with higher levels in stem, lemma and palea.

Leaf development and morphogenesis are regulated by plant hormones, transcriptional regulators and mechanical properties of the tissue. To investigate the CYP roles in the processes, RNA-Seq data were used to quantify the expression levels along the leaf development gradient. *OsCYP87C2*, *OsCYP96B5*, *OsCYP96B8*, *OsCYP84A5*, *OsCYP86B3*, *OsCYP86A11*, *OsCYP77B2*, *OsCYP86A9* and *OsCYP92A14* were highly expressed at leaf base where cell division is active and cell-fate decisions are being made. *OsCYP87A6* was up-regulated in response to light and auxin signaling and in turn decrease local auxin accumulation in coleoptiles [41], but the functions of other CYP87 family members remain obscure. OsCYP87C2 shares 74.3% amino acid identity to AtCYP708A1 which function upstream of AtCYP705A5 and downstream of AtOSC in the biosynthesis of oxygenated triterpenoids [50]. We noted that CYP708 and CYP705 family are not found in rice, therefore a coexpression network with base-specific *OsCYP87C2* as a “guide gene” was constructed using RiceFREND (http://ricefrend.dna.affrc.go.jp/) with mutual rank value <7, hierarchy = 3. The network consists of 56 nodes and 84 weighted edges, of which eight green nodes distribute in the first hierarchy, 21 yellow in the second and 26 blue in the third (Fig. 4b). Co-expression relationships analysis is an effective way to identify the function associations between genes that may be co-regulated and involved in the same biological process. Sclerenchyma cells with thickened SCW were important for maintaining the proper morphology in rice leaves. Of the co-expressed genes (Additional file 18: Table S12), Os01g36460 and Os01g45730 encodes two proteins respectively sharing 78.6% and 92.2% sequence identities to AtMYB26 and AtC3H14 that can regulated MBS- and ARE-containing genes involved in secondary cell wall (SCW) biosynthesis [51, 52]. The two types of *cis*-regulatory elements are also present in *OsCYP87C2* promoter region. Another leaf base-specific expressed *OsCYP92A14* is homologous to pea *CYP92A6* that encodes a BR C-2 hydroxylase involved in the biosynthesis of BR which is associated with cell elongation, cell division, leaf bending, vascular differentiation, proton pump-mediated membrane polarization and sink/source regulation [53, 54]. In maturing zone (sections 5–9) where activities of Calvin Benson cycle enzymes and intercellular metabolite are increased, some *OsCYP*s were highly expressed, such as lineage II and lineage IV-1 genes. *OsCYP97C2*, a homologue of *AtCYP94C1* that encodes a carotenoid ε-ring hydroxylase catalyzing the formation of lutein which modulates light energy and serves as a non-photochemical quenching agents to deal with triplet chlorophyll [55, 56], was expressed at relatively high levels. OsCYP711A5 may catalyze the biosynthesis of SLs which are carotenoid-derived plant hormones and accelerate leaves maturity and senescence [57]. Several highly expressed *OsCYP*s are involved in phenylpropanoid pathway including lignin and flavonoid biosynthetic branches, as shown in Fig. 4c. Among them, OsCYP73A38 is a homologue of AtCYP73A5 which catalyze the hydroxylation of t-cinnamic acid, a key early step in the pathway [58]. Additionally, *OsCYP89B3* is homologous with *AtCYP89A9* which is implicated in the formation of major chlorophyll catabolites during leaf senescence [59].

### Functions of *OsCYP*s in response to drought stress

**Fig. 6:**
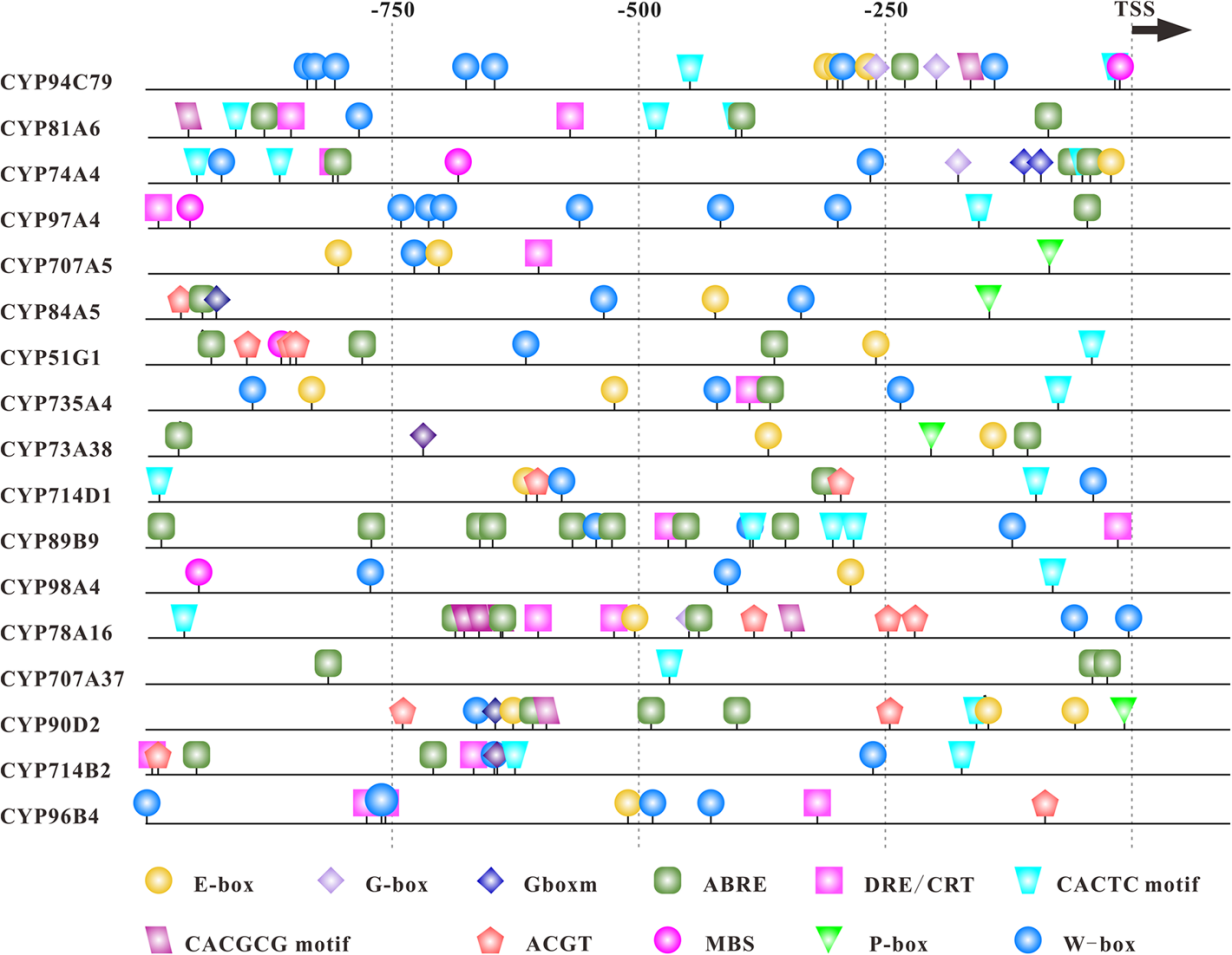
In silico promoter analyses of 17 *OsCYP* genes. Different regulatory elements are indicated by different geometric shape with different colour in their relative position in promoter regions. TSS: Transcriptional Start Site

*OsCYP96B4* and *OsABA8ox3* (*OsCYP707A37*) has been functionally characterized under drought stress [7, 8], whereas a genome-wide investigation of expression and regulation characteristics of *OsCYP*s has not been done. It has been reported that OsbHLH148 (Os03g53020) which was predicted to bind to the G-box acted on an initial response of jasmonate-regulated gene expression toward drought tolerance [60]. *CYP94B* and *CYP94C* can be induced by JA, drought, salinity and other stresses, and their encoded proteins catalyze oxidation of JA-Ile to 12OH-JA-Ile and 12COOH-JA-Ile for catabolic turnover [61]. The relationships between *OsbHLH148* and *CYP*s are worthy of further study. To gain deep insights into the functions of *OsCYP*s responding to drought stress and the regulatory relationships between *OsbHLH148* and *OsCYP*s, RNA-seq data from wild type Nipponbare (WT) and *Osbhlh148* mutant (MT) treated with drought stress were used to analyze the expression patterns of *OsCYP*s. A number of DEGs were identified, and some genes exhibited great variation in their expressions in WT and MT, which suggest that these genes may be regulated by OsbHLH148 directly or indirectly. Plant hormones, transcription factors and other regulatory proteins involved in *OsCYP*s expression regulation under drought stress are presented in Fig. 5b. Adapting to water deficit, the higher levels of endogenous JA and ABA were maintained with their catabolism and biosynthesis activated. RNA-seq data analysis showed that *OsCYP94B*s and *OsCYP94C*s were significantly up-regulated in both WT and MT, while *OsCYP94C79* (Os12g05440) and *OsCYP94C2* (Os11g05380) containing G-box motif in their promoters showed lower transcriptional activity in MT compared to WT. *CYP74As* encode allene oxide synthases (AOS) that catalyzes the JA biosynthesis. The fold change in the mRNA levels of *OsCYP74A4* was higher in WT than in MT, in whose promoter one G-box, one DRE/CRT and three ABREs are found (Fig. 6). In contrast, the transcription levels of *OsCYP97A4* encoding carotenoid β-ring hydroxylase involved in ABA and lutein biosynthesis were decreased in WT and increased in MT. It was reported that *AtCYP707A1*-*AtCYP707A4* encoding 8′-hydroxylases in ABA catabolism were up-regulated under varying degrees of drought stress [62]. Of rice homologues of *AtCYP707As*, only *OsCYP707A5* containing DRE/CRT motif was up-regulated, while *OsCYP707A37* having ABRE motif and *OsCYP707A6* were down-regulated. In this analysis, the ABA-responsive genes *DREB1A-C*, *−G* and *–J*, *OsDREB2A*, *OsDREB2B*, *OsABF1* and *OsABF2* were significantly up-regulated (Fig. 5d). CK level was decreased under dehydration stress, but the mechanisms of interaction between ABA and CK remain largely unknown. Unexpectedly, *OsCYP735A4* encoding cytokinin hydroxylase that catalyzes the biosynthesis of trans-zeatin was up-expressed. DELLA proteins function as an interface of JA, ABA and GA signaling, and activate anthocyanin biosynthesis contributing to stress tolerance through sequestering MYBL2 and JAZ suppressors of MYB/bHLH/WD40 complex in Arabidopsis [63]. XERICO, a zinc finger protein, is a transcriptional downstream target of DELLA proteins and regulates ABA biosynthesis and tolerance to drought in Arabidopsis [64]. Only one DELLA protein OsSLR1 is presented in rice genome, of which mRNA levels were slightly decreased. Overexpression of *OsRHP1,* a homologue of *XERICO*, enhanced drought tolerance through increased ABA level and activated ABA-mediated stress response [65]. It was significantly induced by drought treatment. *OsCYP701A6* encoding *ent*-kaurene oxidase (KO) that catalyzes GA biosynthesis can be trans-activated by REPRESSION OF SHOOT GROWTH (RSG) which is a candidate as a downstream factor of DELLA [66]. Surprisingly, although the transcription level of *RSG* (Os12g06520) was enhanced, the *OsCYP701A6* expression was decreased to very low level under drought condition in our analysis. What’s more, *OsCYP88A5* encoding *ent*-kaurenoic acid oxidase (KAO) and *OsCYP714Bs* encoding GA 13-oxidases, which function in the later GA biosynthetic steps were down-regulated, and *OsCYP714D1* encoding a GA-deactivating enzyme was up-expressed, suggesting that drought stress may result in the decreased levels of bioactive GAs in rice. BR primary signaling outputs can be regulated by ABA signaling, and BRs can induce oxo-phytodienoic reductases (OPRs) in JA biosynthesis [67]. For all we know, at least four CYP families (CYP51, CYP90, CYP724 and CYP85) participate in the BR biosynthesis, and the last three are subject to BR negative feedback [25]. Strikingly, mutation of *Osbhlh184* led to a drastic suppression of expression of the drought-inducible *OsCYP51G3* that contains G-box motif in promoter region. OsCYP85A1 functions downstream of OsCYP51G3, and its encoding gene was induced by dehydration stress. And *OsCYP734A*s that encode hydroxylases connected with inactivation of BRs were down-regulated, particularly *OsCYP734A4*. Dramatically, *OsRAVL1* (Os04g49230) was expressed at low levels, which encodes a key regulator of BR biosynthetic genes (*OsCYP90D2*, *OsCYP724B1* and *OsCYP85A1*) and BR signal transduction pathway [68]. Drought stress signaling can be triggered by the accumulation of reactive oxygen species (ROS) which can integrate with ABA, GA and other hormones signaling [69]. Wheat CYP709C1 is a sub-terminal hydroxylase of long chain fatty acid and can be positively regulated by MeJA signaling, participating in detoxifying process [27]. Two G-box containing genes *OsCYP709C9* and *OsCYP709C5*, homologues of wheat *CYP709C1*, were strongly induced by drought stress in WT, which are confirmed by qPCR, while their expressions were inhibited in MT. In addition, they may be targeted by osa-miR164 which was down-regulated by drought stress.

## Conclusions

We have identified 326 *OsCYP* genes in rice genome, which were classified into 10 clans including 45 families. Our evolutionary analysis of CYP superfamily indicated that *OsCYP* genes have been undergoing frequent duplication and function diversification. This is going to help us to understand the complex agronomic traits and evolution of rice. Meanwhile the species- or families-specific expansions of CYP superfamily are observed, which will contribute to explain speciation events. Our transcriptome analyses based on micrroarray and RAN-seq data revealed that some *OsCYP*s are tissue- and drought-specific genes involved in maintaining hormone homeostasis and the metabolism of fatty acids, steroids, phenylpropanoids and so on. It is useful for understanding a variety of developmental and physiological processes, stress response mechanisms. Our findings will facilitate the functional study of *CYP* genes and accelerate the pace of rice genetic improvement.

## Methods

### Sequence retrieval and identification of OsCYP450

All rice protein sequences were downloaded from MSU/TIGR Rice Genome Annotation Project Release 7 (RGAP, http://rice.plantbiology.msu.edu/). We identified OsCYPs according to the following steps: Firstly, the CYP protein sequences of Arabidopsis and wild rice were retrieved from TAIR (https://www.Arabidopsis.org/) and Cytochrome P450 Homepage, respectively. The full-length CYP sequences were separately aligned for each species with MEGA 6.0 software (http://megasoftware.net/), and the alignments were used to construct Hidden Markov Model based profiles (HMM-profiles) using the hmmbuild program in HMMER package [70]. Besides, we obtained another HMM profile (accession number: PF00067) from the Pfam(http://pfam.xfam.org/family/PF00067). Secondly, the three profiles were used to run searches against the local database with hmmsearch program. Thirdly, the non-redundant candidate protein sequences received a conserved domain check using PROSITE (http://prosite.expasy.org/), SMART (http://smart.embl-heidelberg.de/) and Pfam. Finally, those sequences without complete P450 domain were removed. To obtain official nomenclature for each *OsCYP* gene, we submitted all OsCYP protein sequences to a P450 nomenclature committee (David Nelson: dnelson@uthsc.edu). To predict orthology and paralogy of *OsCYP* genes, the locus IDs were individually searched against the Rice Genome Annotation Project database (http://rice.plantbiology.msu.edu/annotation_pseudo_apk.shtml). A public server TMHMM (http://www.cbs.dtu.dk/services/TMHMM/) was used to predict transmembrane helices in the protein sequences. Both SignalP 4.1 (http://www.cbs.dtu.dk/services/SignalP-4.1/) and LocTree3 (https://rostlab.org/services/loctree3/) were used to predict the protein subcellular localization. The isoelectric points (pIs) and molecular weights (MWs) of these proteins were estimated by ExPASy Server (http://web.expasy.org/compute_pi/). The conserved protein motifs were detected and visualized using MEME tool in Galaxy web-based platform (https://usegalaxy.org/).

### Homology based structural modelling of OsCYP51Gs and OsCYP74As

Homology based models of OsCYP51G1, OsCYP51G3, OsCYP74A4 and OsCYP74A5 were built using SWISS-MODEL program (https://swissmodel.expasy.org/). Each protein sequence was first analyzed by template search, followed by model building using best template structure. The models were evaluated by calculating stereochemical quality via the ERRAT by Structural Analysis and Verification Server (SAVES, http://services.mbi.ucla.edu/SAVES/). Resulting structures were visualized using UCSF Chimera (http://www.cgl.ucsf.edu/chimera/). Substrate recognition sites and regions were predicted and marked based on previous template structure analysis. Additionally, a structure-based sequence alignment of the four OsCYPs and templates was generated with ESPript 3.0 (http://espript.ibcp.fr/ESPript/ESPript/).

### Phylogenetic tree construction and gene structure analysis

To investigate the phylogenetic relationships among the *OsCYP*s, the full-length protein sequences were aligned using ClustalW, and a phylogenetic tree was constructed by Neighbor-Joining (NJ) method, with the robustness of the tree topology assessed by 1000 bootstrap replicates. Finally, the tree was visualized using Interactive Tree Of Life (iTOL) web-based tool (http://itol.embl.de/). The gene annotation file in GFF3 (Generic Feature Format version 3) format was available on RGAP website (http://rice.plantbiology.msu.edu/downloads_gad.shtml). Then the file was loaded into Gene Structure Display Server 2.0 (GSDS, http://gsds.cbi.pku.edu.cn/) for *OsCYP* gene structure visualization. Genomic sequences and corresponding coding sequences (CDS) in FASTA format were uploaded to GSDS to analyze the distribution of intron phases.

### Chromosomal localization and gene duplication events

To determine physical locations of *CYP*s on rice chromosomes, MapDraw, a Microsoft Excel Macro for drawing genetic linkage maps based on given genetic linkage data, was used to visualize the loci of *OsCYP*s. Two genes belonging to the same OsCYP family and separated by fewer than 10 genes on the same chromosome were considered to be tandemly duplicated [71]. And the GoGe Comparative Genomics platform (http://genomevolution.org/CoGe/SynMap.pl) was used to analyze the segmental duplication between two rice chromosomes.

### Selective pressure analyses

Except for CYP727 clan which contains one member, CDS and protein sequences were respectively divided into nine data sets based on cluster analysis. ClustalW was used to align the protein sequences. And the PAL2NAL program (http://www.bork.embl.de/pal2nal/) was used to convert multiple protein sequence alignment into a corresponding codon-based nucleotide alignment. The codon-based nucleotide alignments were used in conjunction with phylogenetic trees generated using the Maximun likelihood (ML) algorithm in MEGA 6 as input for the codeml program in Phylogenetic Analysis by Maximum Likelihood (PAML) to detect positive selection under site models [72]. We considered a site under positive selection if the posterior probability estimated by the Bayes empirical Bayes (BEB) method is greater than 0.95. Meanwhile, SLAC (single likelihood ancestor counting), FEL (fixed-effects likelihood), and REL (random-effects likelihood) implemented in Datamonkey server (http://www.datamonkey.org) were used to identify positively selected sites. Sites with *p*-value smaller than 0.05 (posterior probabilities >0.95) for SLAC and FEL, and Bayes factors larger than 50 for REL were considered as being under positive selection. To further reduce the chance of falsely accepting positively selected sites, we considered sites to be under positive selection if there was consensus between the four models. To illuminate the divergence after gene duplication, the ratio (ω) of non-synonymous to synonymous rates (dN/dS) for each paralogous gene pair was calculated using the codeml program in run mode − 2 to assay the strength and direction of selection [73]. Values of ω (dN/dS) < 1, = 1 and >1 indicate negative selection, neutral evolution and positive selection.

### Regulatory-motif analysis and miRNA-target prediction

The 1000 bp sequences upstream of the translation start codon in the *OsCYP* promoters were extracted from rice genome sequence using custom BioPerl script, and PATCH™ (http://www.gene-regulation.com/pub/programs.html) was used to find out putative *cis*-acting elements and predict potential transcription factor binding sites (TFBS). Furthermore, eleven different stress-responsive elements in seventeen selected genes were precisely illustrated using IBS software (http://ibs.biocuckoo.org/download.php). To predict the potential *OsCYP* mRNA target of miRNA, the rice mature miRNA sequences deposited in the miRBase database (http://www.mirbase.org/index.shtml) were downloaded, and then the miRNA and cDNA sequences were uploaded into Target-align with default settings (a score cut-off of ≥70 and mismatch ≤4 nucleotides) [74].

### Microarray-based gene expression profiling throughout the growth cycle

Microarray gene expression data were available at the Rice Expression Profile Database (RiceXPro, http://ricexpro.dna.affrc.go.jp/). Each gene expression data file consists of reporter identifiers and signal intensity values subjected to 75 percentile normalization and log2 transformation. We determined the gene expression profiles for 62 distinct tissues representing 12 major organ systems of *Oryza sativa* cv. Nipponbare using microarrays containing 43,734 probe sets corresponding to about 40,000 genes [75]. A total of 312 MSU/TIGR locus IDs of *OsCYP*s were converted into RAP IDs in advance through BioMart data mining tool in Ensembl Plants website (http://plants.ensembl.org/index.html) and ID converter tool in the Rice Annotation Project Database (RAP-DB, http://rapdb.dna.affrc.go.jp/). Then the arithmetic means were calculated over all replicated samples from the same tissue. Tissue selectivity of each transcript was assessed by computing the coefficient of variation of signal intensity (CV = SD/mean) in the 62 tissues. We defined tissue-specific genes as those with CV ≥1 and expression values ≥1 in any tissue. To visualize gene expression atlas in a concise way, the heatmap was generated using heatmap.2 function from the gplots package in R 3.2.3 (https://cran.r-project.org/web/packages/gplots/index.html).

To further understand the expression patterns and potential functions of *OsCYP*s undergoing leaf development trajectories (maturing from the tip to base), we utilized the gene expression data generated in a RNA-seq experiment conducted by Lin Wang, et al. [76]. The RNA-seq data sets were downloaded from NCBI GEO (https://www.ncbi.nlm.nih.gov/geo/index.cgi) with accession number GSE54274. The gene expression levels were quantified using FPKM values (Fragments Per Kilobase of exon model per Million mapped reads). In order to avoid negative values after the log2 transformation of FPKM values, the arithmetic mean of FPKM values over all samples from the same tissue were calculated and then the mean lower than 1 were replaced by 1 prior to log2 transformation. *OsCYP*s expressed in the eleven continuous leaf sections with FPKM CV ≥ 1 were considered as tissue-specific genes. Finally, the log2-transformed FPKM values were used to create hierarchical clustering heatmap using heatmap.2 function in the gplots package. *UBQ5, Eef-1α* (Os03g08010) and *14–3-3* (Os11g34450) were used as internal controls.

### Expression analysis of *OsCYP450s* under drought stress using RNA-seq data

The RNA-seq data from wild type (WT) Nipponbare and *Osbhlh148* mutant type (MT) under drought treatment were downloaded from NCBI GEO with accession number GSE65024 to analyze the expression patterns of *OsCYP*s. Differentially expressed genes (DEGs) were defined as those that satisfy the two criteria, the fold change (FC) cut-offs of >2 and significant q-values (FDR) of <0.05. The log2 (FC) values of DEGs were used for hierarchical cluster analysis by applying an adjusted heatmap.2 function. Additionally, the FPKM values of differentially expressed *OsCYP*s in WT and *Osbhlh148* mutant under 10-day drought stress were displayed in the form of a line charts superimposed onto the heatmap.

### Plant materials and qPCR validation

Rice (*Oryza satva* L.ssp. *japonica* cv. Nipponbare) plants were grown in soil mixtures under greenhouse conditions with 25 °C day /22 °C night temperature, 600 mmol/m2/s light intensity and 14 h light and 10 h dark cycles. All pots were placed in water-filled trays to simulate flooded condition and fertilized every week until 45-day old. To control drought stress, following gravimetric approach was applied on 45 day old plants. The soil water content was brought down to 40% field capacity over a period of 3–4 days and plants were maintained at that level for 10 days by weighing the pots daily at a fixed time of the day and replenishing the water lost through evapotranspiration. Well-watered plants were maintained at 100% field capacity. The fresh leaf samples were harvested from the well-watered and drought-stressed rice and frozen in liquid nitrogen immediately. Total RNA was isolated, and single-stranded cDNA was synthesized using PrimeScript™ RT reagent Kit (Perfect Real Time). The qPCR reaction was performed with SYBR® *Premix Ex Taq*™ II (Tli RNaseH Plus) using specific primers. For all experiments, three biological replicates of each sample were used. The relative genes expression levels were evaluated using 2^-ΔCt^ method, and the *UBQ5* (Os06g44080) was used as an internal control for normalization.

## Additional files

Additional file 1: Table S1. The identified *OsCYP*s and their related information. (PDF 131 kb)

Additional file 2: Figure S1. Conserved motifs analysis of OsCYPs. Each motif is represented by a colored box. Box length corresponds to motif length. (PDF 14130 kb)

Additional file 3: Table S2. Motif with best possible match and its number in each subfamily. (PDF 56 kb)

Additional file 4: Figure S2. ERRAT (v 4.0) values showed reasonable scores from SAVES. (PDF 657 kb)

Additional file 5: Figure S3. Multiple sequence alignment of HmCYP51, OsCYP51G1, OsCYP51G3, OsCYP74A5, OsCYP74A4 and AtCYP74A1. Assignment of secondary structure elements is based on HmCYP51 and AtCYP74A1structures. Black frames localize Gotoh’s SRS regions 1–6 identified based on sequence alignment with P450cam, and triangles point to residues constituting the substrate binding site of HmCYP51 (red) and AtCYP74A1 (blue). (PDF 903 kb)

Additional file 6: Figure S4. Phylogenetic relationships and gene structural features of *OsCYP*s. a A unrooted NJ tree of *OsCYP*s was constructed using the MEGA6 software and visualized by iTOL. Bootstrap values larger than 75% are shown. b Gene structures of *OsCYP*s. 0, 1, 2 stand for the types of intron phase. The length of the intron, exon, and UTR could be estimated based on the scale. (PDF 2230 kb)

Additional file 7: Figure S5. The distribution of CYP family sizes in whole genome for 10 species. a A-type CYP families. b Non-A type CYP families. The time tree generated using TIMETREE web resource (http://www.timetree.org/) revealed the clock-like speciation and diversification of the ten species. Sm: *Salvia miltiorrhiza*; Pa: *Populus alba*; Nn: *Nelumbo nucifera*; Vv: *Vitis vinifera*; Cp: *Carica papaya*; Os: *Oryza sativa*; Gm: *Glycine max*; Cc: *Citrus clementina*; At: *Arabidopsis thaliana*; Bd: *Brachypodium distachyon*. The four species, Nn, Sm, Pa and Cc, were instead by other species of the same genus on the time tree. (PDF 439 kb)

Additional file 8: Table S3. Log-likelihood values and parameters estimates for the CYP71 clan under site-specific models. (PDF 54 kb)

Additional file 9: Table S4. Log-likelihood values and parameters estimates for the CYP86 clan under site-specific models. (PDF 53 kb)

Additional file 10: Table S5. Log-likelihood values and parameters estimates for the CYP710 clan under site-specific models. (PDF 53 kb)

Additional file 11: Table S6. Log-likelihood values and parameters estimates for the CYP72 clan under site-specific models. (PDF 53 kb)

Additional file 12: Table S7. Log-likelihood values and parameters estimates for the CYP711 clan under site-specific models. (PDF 53 kb)

Additional file 13: Table S8. List of the positively selected sites detected by DATAMONKEY. SLAC: Single likelihood ancestor counting, FEL: fixed-effects likelihood, REL: random-effects likelihood (posterior probabilities more than 0.95; Bayes factors >50). (PDF 53 kb)

Additional file 14: Figure S6. Scatter plot of dN/dS ratio of 230 homologous gene pairs. The letters N, S, T on the right legend signify No duplication, Segmental duplication and Tandem duplication of the pairs, respectively. The different colors stand for different clans. (PDF 403 kb)

Additional file 15: Table S9. List of the number of potential *cis*-acting elements in 326 *OsCYP* promoter regions. (PDF 94 kb)

Additional file 16: Table S10. List of predicted miRNA-OsCYP pairs with target regions and assessing results. (PDF 137 kb)

Additional file 17: Table S11. List of primers for qPCR used in this study. (PDF 45 kb)

Additional file 18: Table S12. Annotation of co-expressed genes. (PDF 67 kb)

## Abbreviations

ABREs: ABA-responsive cis-acting elements
CV: Coefficient of variation
DEGs: Differentially expressed genes
DREBs: Dehydration responsive element binding proteins
ER: Endoplasmic reticulum
FC: Fold change
HMM: Hidden Markov Model
Mws: Molecular weights
P450: Cytochrome P450 monooxygenase
pIs: Isoelectric points
ROS: Reactive oxygen species
SCW: Secondary cell wall
SRSs: Substrate recognition sites
TFBS: Transcription factor binding sites

## References

[1] Frear DS, Swanson HR, Tanaka FS (1969). *N*-Demethylation of substituted 3-(phenyl)-1-Methylureas: isolation and characterization of a microsomal mixed function oxidase from cotton. Phytochemistry.

[2] Liu Z, Boachon B, Lugan R, Tavares R, Erhardt M, Mutterer J, Demais V, Pateyron S, Brunaud V, Ohnishi T (2015). A conserved cytochrome P450 evolved in seed plants regulates flower maturation. Mol Plant.

[3] Shi J, Cui M, Yang L, Kim YJ, Zhang D (2015). Genetic and biochemical mechanisms of Pollen Wall development. Trends Plant Sci.

[4] Ito T, Meyerowitz EM (2000). Overexpression of a gene encoding a cytochrome P450, *CYP78A9*, induces large and seedless fruit in Arabidopsis. Plant Cell.

[5] Chen Y, Liu L, Shen Y, Liu S, Huang J, Long Q, Wu W, Yang C, Chen H, Guo X (2015). Loss of function of the cytochrome P450 gene *CYP78B5* causes Giant embryos in Rice. Plant Mol Biol Report.

[6] Mizutani M, Ohta D (2010). Diversification of P450 genes during land plant evolution. Annu Rev Plant Biol.

[7] Cai S, Jiang G, Ye N, Chu Z, Xu X, Zhang J, Zhu G (2015). A key ABA catabolic gene, OsABA8ox3, is involved in drought stress resistance in Rice. PLoS One.

[8] Tamiru M, Undan JR, Takagi H, Abe A, Yoshida K, Undan JQ, Natsume S, Uemura A, Saitoh H, Matsumura H (2015). A cytochrome P450, OsDSS1, is involved in growth and drought stress responses in Rice (*Oryza sativa* L**.)**. Plant Mol Biol.

[9] Ramamoorthy R, Jiang SY, Ramachandran S (2011). *Oryza sativa* cytochrome P450 family member OsCYP96B4 reduces plant height in a transcript dosage dependent manner. PLoS One.

[10] Wang X, Cheng Z, Zhao Z, Gan L, Qin R, Zhou K, Ma W, Zhang B, Wang J, Zhai H, Wan J (2016). *BRITTLE SHEATH1* Encoding OsCYP96B4 is involved in Secondary cell wall formation in rice. Plant Cell Rep.

[11] Mao G, Timothy S, Denyse S, Yu O (2013). CYP709B3, a cytochrome P450 monooxygenase gene involved in salt tolerance in *Arabidopsis thaliana*. BMC Plant Biol.

[12] Siminszky B, Corbin FT, Ward ER, Fleischmann TJ, Dewey RE (1999). Expression of a soybean cytochrome P450 monooxygenase cDNA in yeast and tobacco enhances the metabolism of Phenylurea herbicides. Proc Natl Acad Sci U S A.

[13] Persans MW, Wang J, Schuler MA (2001). Characterization of maize cytochrome P450 monooxygenases induced in response to Safeners and bacterial pathogens. Plant Physiol.

[14] Williams PA, Cosme J, Sridhar V, Johnson EF, McRee DE (2000). Mammalian microsomal cytochrome P450 monooxygenase: structural adaptations for membrane binding and functional diversity. Mol Cell.

[15] Chehab EW, Raman G, Walley JW, Perea JV, Banu G, Theg S, Dehesh K (2006). Rice *HYDROPEROXIDE LYASES* with unique expression patterns generate distinct aldehyde signatures in Arabidopsis. Plant Physiol.

[16] Quinlan RF, Shumskaya M, Bradbury LM, Beltrán J, Ma C, Kennelly EJ, Wurtzel ET (2012). Synergistic interactions between carotene ring hydroxylases drive lutein formation in plant carotenoid biosynthesis. Plant Physiol.

[17] Hasemann CA, Kurumbail RG, Boddupalli SS, Peterson JA, Deisenhofer J (1995). Structure and function of cytochromes P450: a comparative analysis of three crystal structures. Structure.

[18] Nelson DR, Schuler MA, Paquette SM, Werck-Reichhart D, Bak S (2004). Comparative genomics of Rice and Arabidopsis. Analysis of 727 cytochrome P450 genes and pseudogenes from a monocot and a dicot. Plant Physiol.

[19] Ehlting J, Sauveplane V, Olry A, Ginglinger JF, Provart NJ, Werck-Reichhart D (2008). An extensive (co-)expression analysis tool for the cytochrome P450 superfamily in *Arabidopsis thaliana*. BMC Plant Biol.

[20] Lee DS, Nioche P, Hamberg M, Raman CS (2008). Structural insights into the evolutionary paths of Oxylipin biosynthetic enzymes. Nature.

[21] Thomas JH (2007). Rapid birth-death evolution specific to xenobiotic cytochrome P450 genes in vertebrates. PLoS Genet.

[22] Xia K, Ou X, Tang H, Wang R, Wu P, Jia Y, Wei X, Xu X, Kang SH, Kim SK, Zhang M (2015). Rice microRNA osa-miR1848 targets the Obtusifoliol 14α-demethylase gene OsCYP51G3 and mediates the biosynthesis of Phytosterols and Brassinosteroids during development and in response to stress. New Phytol.

[23] Sunkar R, Zhou X, Yun Z, Zhang W, Zhu JK (2008). Identification of novel and candidate miRNAs in Rice by high throughput sequencing. BMC Plant Biol.

[24] Stumpe M, Feussner I (2006). Formation of Oxylipins by CYP74 enzymes. Phytochem Rev.

[25] Vriet C, Russinova E, Reuzeau C (2013). From squalene to Brassinolide: the steroid metabolic and signaling pathways across the plant kingdom. Mol Plant.

[26] Nelson D, Werck-Reichhart D (2011). A P450-centric view of plant evolution. Plant J.

[27] Kandel S, Morant M, Benveniste I, Blee E, Werck-Reichhart D, Pinot F (2005). Cloning, functional expression, and characterization of CYP709C1, the first sub-terminal hydroxylase of long chain fatty acid in plants. INDUCTION BY CHEMICALS AND METHYL JASMONATE. J Biol Chem.

[28] Sakamoto T, Kawabe A, Tokida-Segawa A, Shimizu B, Takatsuto S, Shimada Y, Fujioka S, Mizutani M (2011). Rice CYP734As Function as Multisubstrate and Multifunctional Enzymes in Brassinosteroid Catabolism. Plant J.

[29] Magome H, Nomura T, Hanada A, Takeda-Kamiya N, Ohnishi T, Shinma Y, Katsumata T, Kawaide H, Kamiya Y, Yamaguchi S (2013). *CYP714B1* and *CYP714B2* encode gibberellin 13-oxidases that reduce gibberellin activity in Rice. Proc Natl Acad Sci U S A.

[30] Takei K, Yamaya T, Sakakibara H (2004). ***Arabidopsis CYP735A1*** and *CYP735A2* Encode Cytokinin Hydroxylases That Catalyze the Biosynthesis of *trans*-Zeatin. J Biol Chem.

[31] Lv MZ, Chao DY, Shan JX, Zhu MZ, Shi M, Gao JP, Lin HX (2012). Rice carotenoid β-ring hydroxylase CYP97A4 is involved in lutein biosynthesis. Plant Cell Physiol.

[32] Li H, Pinot F, Sauveplane V, Werck-Reichhart D, Diehl P, Schreiber L, Franke R, Zhang P, Chen L, Gao Y (2010). Cytochrome P450 family member CYP704B2 catalyzes the ω-hydroxylation of fatty acids and is required for anther Cutin biosynthesis and pollen Exine formation in Rice. Plant Cell.

[33] Heitz T, Widemann E, Lugan R, Miesch L, Ullmann P, Desaubry L, Holder E, Grausem B, Kandel S, Miesch M (2012). Cytochromes P450 CYP94C1 and CYP94B3 catalyze two successive oxidation steps of plant hormone Jasmonoyl-isoleucine for catabolic turnover. J Biol Chem.

[34] Zhang Y, van Dijk AD, Scaffidi A, Flematti GR, Hofmann M, Charnikhova T, Verstappen F, Hepworth J, van der Krol S, Leyser O (2014). Rice cytochrome P450 MAX1 homologs catalyze distinct steps in Strigolactone biosynthesis. Nat Chem Biol.

[35] Sauveplane V, Kandel S, Kastner PE, Ehlting J, Compagnon V, Werck-Reichhart D, Pinot F (2009). *Arabidopsis thaliana* CYP77A4 is the first cytochrome P450 able to catalyze the epoxidation of free fatty acids in plants. FEBS J.

[36] Wang Q, Hillwig ML, Wu Y, Peters RJ (2012). CYP701A8: A Rice ent-Kaurene Oxidase Paralog Diverted to More Specialized Diterpenoid Metabolism. Plant Physiol.

[37] Giddings LA, Liscombe DK, Hamilton JP, Childs KL, DellaPenna D, Buell CR, O'Connor SE (2011). A stereoselective hydroxylation step of alkaloid biosynthesis by a unique cytochrome P450 in *Catharanthus roseus*. J Biol Chem.

[38] Tanabe S, Ashikari M, Fujioka S, Takatsuto S, Yoshida S, Yano M, Yoshimura A, Kitano H, Matsuoka M, Fujisawa Y, Kato H, Iwasaki Y (2005). A novel cytochrome P450 is implicated in Brassinosteroid biosynthesis via the characterization of a Rice dwarf mutant, *dwarf11*, with reduced seed length. Plant Cell.

[39] Xu J, Ding Z, Vizcay-Barrena G, Shi J, Liang W, Yuan Z, Werck-Reichhart D, Schreiber L, Wilson ZA, Zhang D (2014). ABORTED MICROSPORES Acts as a master regulator of Pollen Wall formation in *Arabidopsis*. Plant Cell.

[40] Yang X, Wu D, Shi J, He Y, Pinot F, Grausem B, Yin C, Zhu L, Chen M, Luo Z, Liang W, Zhang D (2014). Rice CYP703A3, a Cytochrome P450 Hydroxylase, is essential for development of anther cuticle and pollen exine. J Integr Plant Biol.

[41] Chaban C, Waller F, Furuya M, Nick P (2003). Auxin responsiveness of a novel cytochrome P450 in Rice coleoptiles. Plant Physiol.

[42] Feng XL, Ni WM, Elge S, Mueller-Roeber B, ZH X, Xue HW (2006). Auxin flow in anther filaments is critical for pollen grain development through regulating pollen mitosis. Plant Mol Biol.

[43] Wellesen K, Durst F, Pinot F, Benveniste I, Nettesheim K, Wisman E, Steiner-Lange S, Saedler H, Yephremov A (2001). Functional analysis of the *LACERATA* gene of Arabidopsis provides evidence for different roles of fatty acid *ω*-hydroxylation in development. Proc Natl Acad Sci U S A.

[44] Fich EA, Segerson NA, Rose JK (2016). The plant polyester Cutin: biosynthesis, structure, and biological roles. Annu Rev Plant Biol.

[45] Luo A, Qian Q, Yin H, Liu X, Yin C, Lan Y, Tang J, Tang Z, Cao S, Wang X (2006). EUI1, encoding a putative cytochrome P450 monooxygenase, regulates internode elongation by modulating gibberellin responses in Rice. Plant Cell Physiol.

[46] Wang Q, Hillwig ML, Peters RJ (2011). CYP99A3: Functional identification of a diterpene Oxidase from the momilactone biosynthetic gene cluster in rice. Plant J.

[47] Nagasawa N, Hibara K, Heppard EP, Vander Velden KA, Luck S, Beatty M, Nagato Y, Sakai H (2013). *GIANT EMBRYO* Encodes CYP78A13, required for proper size balance between embryo and endosperm in Rice. Plant J.

[48] Wang X, Li Y, Zhang H, Sun G, Zhang W, Qiu L (2015). Evolution and association analysis of *GmCYP78A10* gene with seed size/weight and pod number in soybean. Mol Biol Rep.

[49] Wang B, Zhou G, Xin Z, Ji R, Lou Y (2015). (*Z*)-3-Hexenal, one of the green leaf volatiles, increases susceptibility of Rice to the white-backed Planthopper *Sogatella furcifera*. Plant Mol Biol Report.

[50] Field B, Osbourn AE (2008). Metabolic diversification--independent assembly of operon-like gene clusters in different plants. Science.

[51] Yang C, Xu Z, Song J, Conner K, Vizcay Barrena G, Wilson ZA (2007). *Arabidopsis MYB26/MALE STERILE35* Regulates Secondary Thickening in the Endothecium and Is Essential for Anther Dehiscence. Plant Cell.

[52] Chai G, Kong Y, Zhu M, Yu L, Qi G, Tang X, Wang Z, Cao Y, Yu C, Zhou G (2015). Arabidopsis C3H14 and C3H15 have overlapping roles in the regulation of secondary wall thickening and anther development. J Exp Bot.

[53] Kang JG, Yun J, Kim DH, Chung KS, Fujioka S, Kim JI, Dae HW, Yoshida S, Takatsuto S, Song PS (2001). Light and Brassinosteroid signals are integrated via a dark-induced small G protein in etiolated seedling growth. Cell.

[54] Nagata N, Asami T, Yoshida S (2001). Brassinazole, an inhibitor of Brassinosteroid biosynthesis, inhibits development of secondary xylem in cress plants (*Lepidium sativum*). Plant Cell Physiol.

[55] Ding F, Wang M, Liu B, Zhang S (2017). Exogenous melatonin mitigates Photoinhibition by accelerating non-photochemical quenching in tomato seedlings exposed to moderate light during chilling. Front Plant Sci.

[56] Tian L, Musetti V, Kim J, Magallanes-Lundback M, DellaPenna D (2004). The *Arabidopsis LUT1* locus encodes a member of the cytochrome p450 family that is required for carotenoid *ε*-ring hydroxylation activity. Proc Natl Acad Sci U S A.

[57] Ueda H, Kusaba M (2015). Strigolactone regulates leaf senescence in concert with ethylene in Arabidopsis. Plant Physiol.

[58] Pan Y, Michael TP, Hudson ME, Kay SA, Chory J, Schuler MA (2009). Cytochrome P450 monooxygenases as reporters for circadian-regulated pathways. Plant Physiol.

[59] Christ B, Süssenbacher I, Moser S, Bichsel N, Egert A, Müller T, Kräutler B, Hörtensteiner S (2013). Cytochrome P450 CYP89A9 is involved in the formation of major chlorophyll catabolites during leaf senescence in *Arabidopsis*. Plant Cell.

[60] Seo JS, Joo J, Kim MJ, Kim YK, Nahm BH, Song SI, Cheong JJ, Lee JS, Kim JK, Choi YD (2011). OsbHLH148, a basic helix-loop-helix protein, interacts with OsJAZ proteins in a Jasmonate signaling pathway leading to drought tolerance in Rice. Plant J.

[61] Koo AJ, Cooke TF, Howe GA (2011). Cytochrome P450 CYP94B3 mediates catabolism and inactivation of the plant hormone Jasmonoyl-L-isoleucine. Proc Natl Acad Sci U S A.

[62] Kushiro T, Okamoto M, Nakabayashi K, Yamagishi K, Kitamura S, Asami T, Hirai N, Koshiba T, Kamiya Y, Nambara E (2004). The *Arabidopsis* cytochrome P450 CYP707A encodes ABA 8′-hydroxylases: key enzymes in ABA catabolism. EMBO J.

[63] Xie Y, Tan H, Ma Z, Huang J (2016). DELLA Proteins Promote anthocyanin biosynthesis via sequestering MYBL2 and JAZ suppressors of the MYB/bHLH/WD40 complex in *Arabidopsis thaliana*. Mol Plant.

[64] Ariizumi T, Hauvermale AL, Nelson SK, Hanada A, Yamaguchi S, Steber CM, Lifting DELLA (2013). Repression of Arabidopsis seed germination by nonproteolytic gibberellin signaling. Plant Physiol.

[65] Zeng DE, Hou P, Xiao F, Liu Y (2014). Overexpressing a novel RING-H2 finger protein gene, *OsRHP1*, enhances drought and salt tolerance in Rice (*Oryza sativa* L.). J Plant Biol.

[66] Gomi K, Matsuoka M (2003). Gibberellin Signalling pathway. Curr Opin Plant Biol.

[67] Bostock RM, Pye MF, Roubtsova TV (2014). Predisposition in plant disease: exploiting the nexus in abiotic and biotic stress perception and response. Annu Rev Phytopathol.

[68] Je BI, Piao HL, Park SJ, Park SH, Kim CM, Xuan YH, Park SH, Huang J, Do Choi Y, An G (2010). *RAV-Like1* maintains Brassinosteroid homeostasis via the coordinated activation of *BRI1* and biosynthetic genes in Rice. Plant Cell.

[69] Golldack D, Li C, Mohan H, Probst N (2014). Tolerance to drought and salt stress in plants: unraveling the signaling networks. Front Plant Sci.

[70] Finn RD, Clements J, Arndt W, Miller BL, Wheeler TJ, Schreiber F, Bateman A, Eddy SR (2015). HMMER Web Server: 2015 Update. Nucleic Acids Res.

[71] Wei K, Pan S (2014). Maize protein phosphatase gene family: identification and molecular characterization. BMC Genomics.

[72] Yang Z (2007). PAML 4: Phylogenetic analysis by maximum likelihood. Mol Biol Evol.

[73] Harikrishnan SL, Pucholt P, Berlin S (2015). Sequence and gene expression evolution of paralogous genes in willows. Sci Rep.

[74] Xie F, Zhang B (2010). Target-align: a tool for plant microRNA target identification. Bioinformatics.

[75] Sato Y, Antonio B, Namiki N, Motoyama R, Sugimoto K, Takehisa H, Minami H, Kamatsuki K, Kusaba M, Hirochika H (2011). Field transcriptome revealed critical developmental and physiological transitions involved in the expression of growth potential in japonica Rice. BMC Plant Biol.

[76] Wang L, Czedik-Eysenberg A, Mertz RA, Si Y, Tohge T, Nunes-Nesi A, Arrivault S, Dedow LK, Bryant DW, Zhou W (2014). Comparative analyses of C4 and C3 photosynthesis in developing leaves of maize and rice. Nat Biotechnol.

