# Supplementary material for: Global identification, structural analysis and expression characterization of cytochrome P450 monooxygenase superfamily in rice
Source: BMC Genomics. 2018 Jan 10;19:35. doi: 10.1186/s12864-017-4425-8 (PMC5764023; doi:10.1186/s12864-017-4425-8)
Supplement: Supplementary file 2 — Conserved motifs analysis of OsCYPs. Each motif is represented by a colored box. Box length corresponds to motif length. (PDF 14130 kb) [file 12864_2017_4425_MOESM2_ESM.pdf]

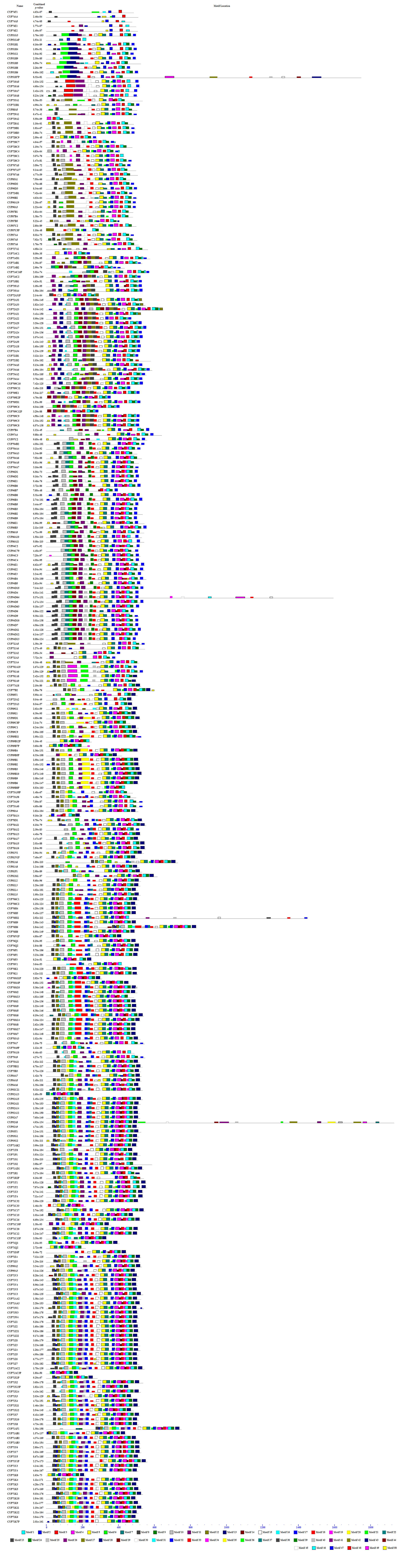

Figure S1. Conserved motifs analysis of OsCYPs. Each motif is represented by a colored box. Box length corresponds to motif length.
