# Supplementary figures and images for: Global identification, structural analysis and expression characterization of cytochrome P450 monooxygenase superfamily in rice
Source: BMC Genomics. 2018 Jan 10;19:35. doi: 10.1186/s12864-017-4425-8 (PMC5764023; doi:10.1186/s12864-017-4425-8)

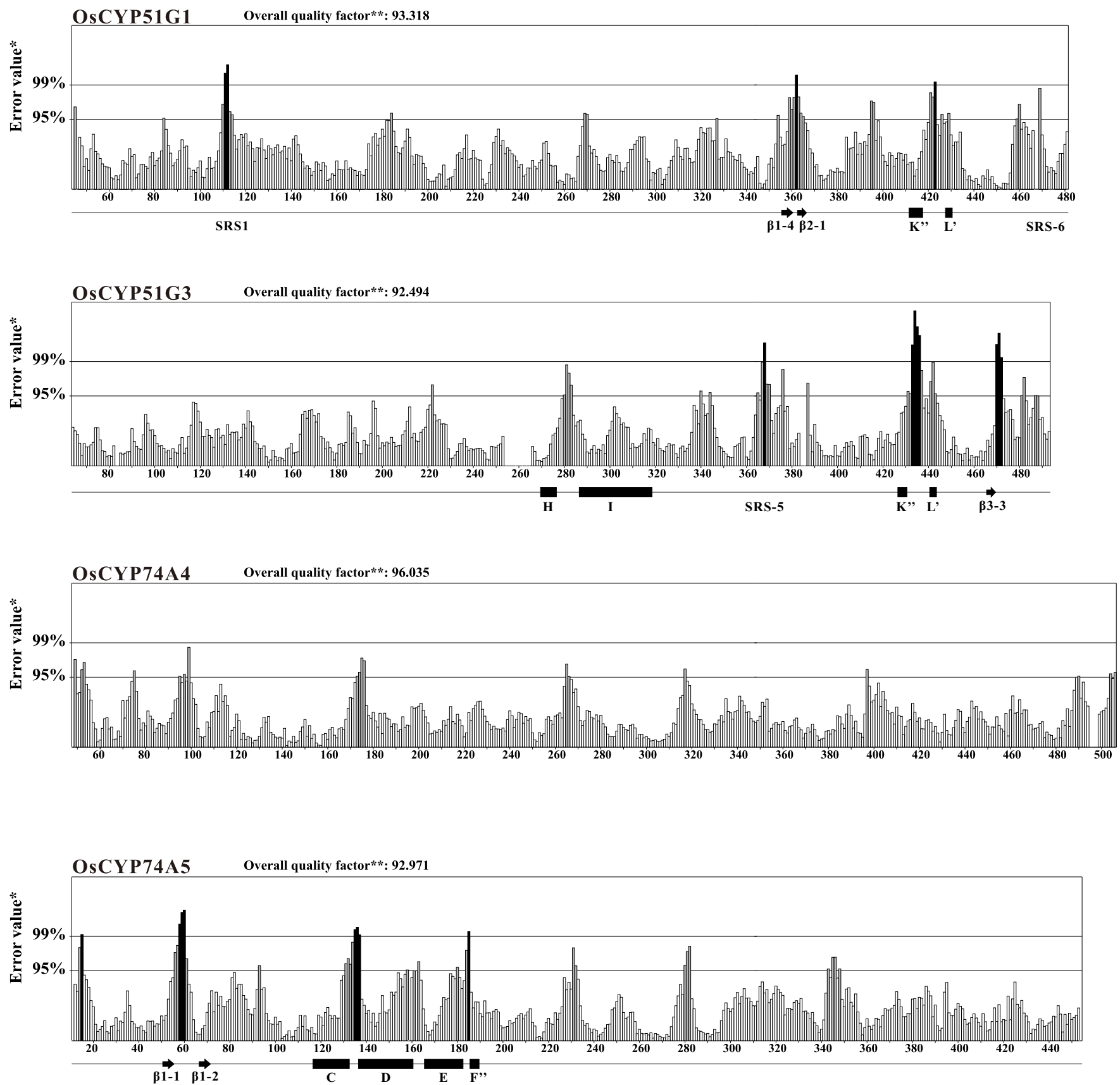

**Figure S2.** ERRAT (v 4.0) values showed reasonable scores from SAVES.

Supplement: Supplementary file 4 — ERRAT (v 4.0) values showed reasonable scores from SAVES. (PDF 657 kb) [file 12864_2017_4425_MOESM4_ESM.pdf]
