# Supplementary material for: Global identification, structural analysis and expression characterization of cytochrome P450 monooxygenase superfamily in rice
Source: BMC Genomics. 2018 Jan 10;19:35. doi: 10.1186/s12864-017-4425-8 (PMC5764023; doi:10.1186/s12864-017-4425-8)
Supplement: Supplementary file 6 — Phylogenetic relationships and gene structural features of OsCYPs. a A unrooted NJ tree of OsCYPs was constructed using the MEGA6 software and visualized by iTOL. Bootstrap values larger than 75% are shown. b Gene structures of OsCYPs. 0, 1, 2 stand for the types of intron phase. The length of the intron, exon, and UTR could be estimated based on the scale. (PDF 2230 kb) [file 12864_2017_4425_MOESM6_ESM.pdf]

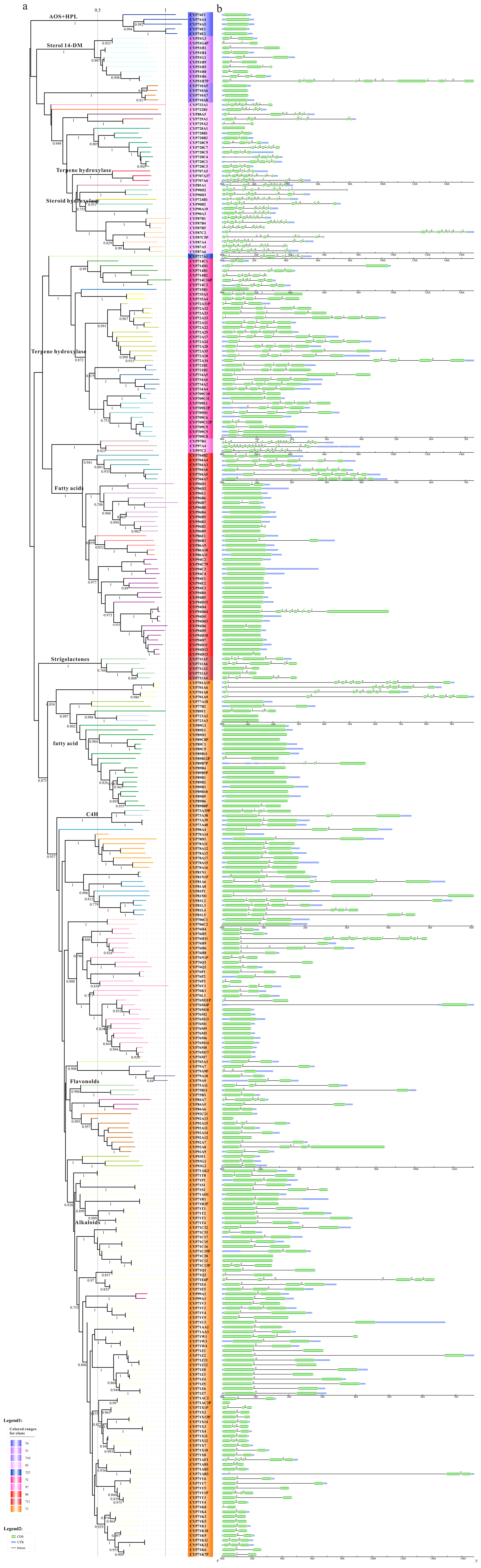

Figure S4. Phylogenetic relationships and gene structural features of *OsCYPs*. **a** A unrooted NJ tree of *OsCYPs* was constructed using the MEGA6 software and visualized by iTOL. Bootstrap values larger than 75% are shown. **b** Gene structures of *OsCYPs*. 0, 1, 2 stand for the types of intron phase. The length of the intron, exon, and UTR could be estimated based on the scale.
