# Supplementary material for: Global identification, structural analysis and expression characterization of cytochrome P450 monooxygenase superfamily in rice
Source: BMC Genomics. 2018 Jan 10;19:35. doi: 10.1186/s12864-017-4425-8 (PMC5764023; doi:10.1186/s12864-017-4425-8)
Supplement: Supplementary file 7 — The distribution of CYP family sizes in whole genome for 10 species. a A-type CYP families. b Non-A type CYP families. The time tree generated using TIMETREE web resource (http://www.timetree.org/) revealed the clock-like speciation and diversification of the ten species. Sm: Salvia miltiorrhiza; Pa: Populus alba; Nn: Nelumbo nucifera; Vv: Vitis vinifera; Cp: Carica papaya; Os: Oryza sativa; Gm: Glycine max; Cc: Citrus clementina; At: Arabidopsis thaliana; Bd: Brachypodium distachyon. The four species, Nn, Sm, Pa and Cc, were instead by other species of the same genus on the time tree. (PDF 439 kb) [file 12864_2017_4425_MOESM7_ESM.pdf]

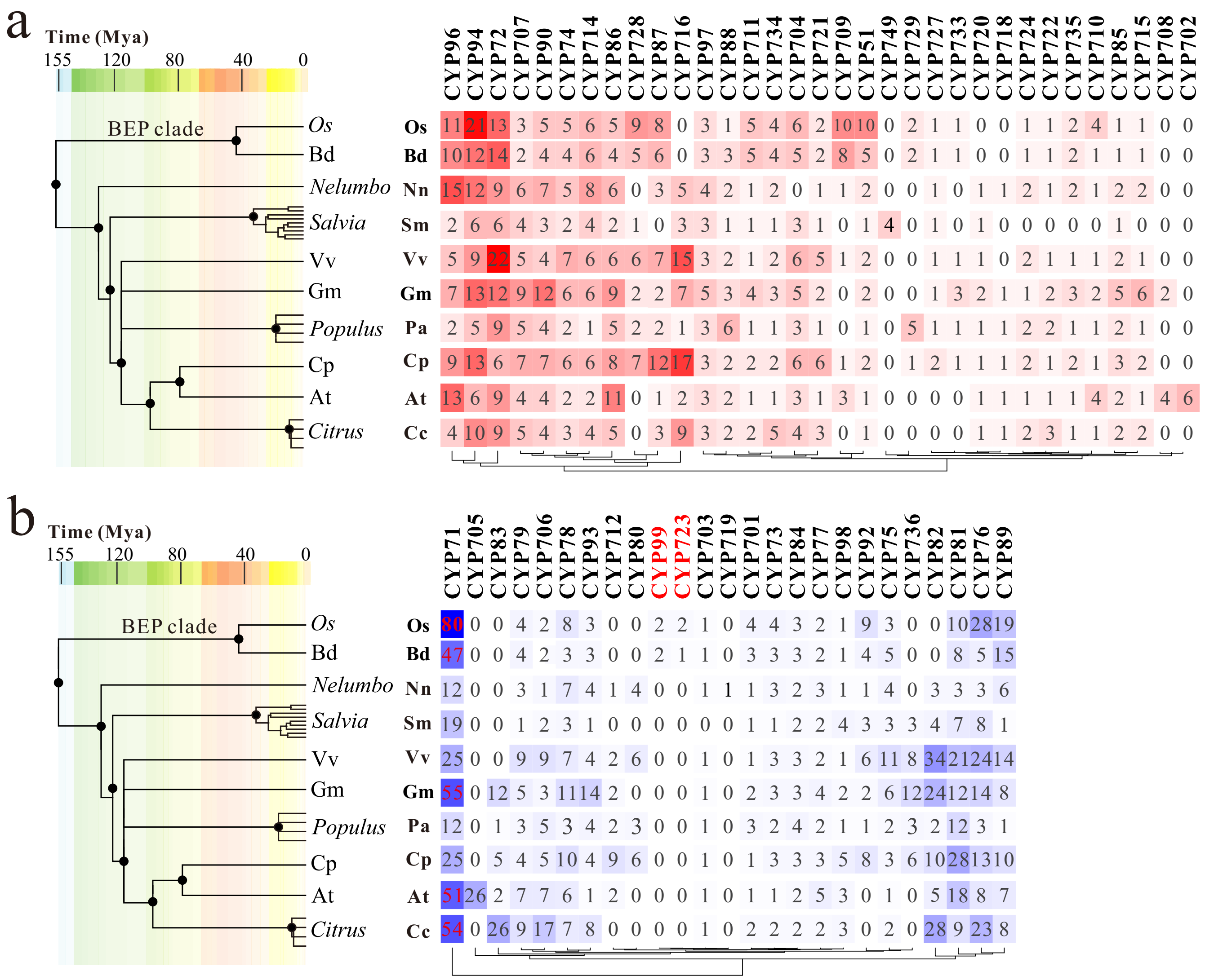

Figure S5. The distribution of CYP family sizes in whole genome for 10 species. **a** A-type CYP families. **b** Non-A type CYP families. The time tree generated using TIMETREE web resource (http://www.timetree.org/) revealed the clock-like speciation and diversification of the ten species. Sm: Salvia miltiorrhiza; Pa: Populus alba; Nn: Nelumbo nucifera; Vv: Vitis vinifera; Cp: Carica Papaya; Os: Oryza sativa; Gm: Glycine max; Cc: Citrus clementina; At: Arabidopsis thaliana; Bd: Brachypodium distachyon. The four species, Nn, Sm, Pa and Cc, were instead by other species of the same genus on the time tree.
