# Supplementary material for: Global identification, structural analysis and expression characterization of cytochrome P450 monooxygenase superfamily in rice
Source: BMC Genomics. 2018 Jan 10;19:35. doi: 10.1186/s12864-017-4425-8 (PMC5764023; doi:10.1186/s12864-017-4425-8)
Supplement: Supplementary file 8 — Log-likelihood values and parameters estimates for the CYP71 clan under site-specific models. (PDF 54 kb) [file 12864_2017_4425_MOESM8_ESM.pdf]

Table S3. Log-likelihood values and parameters estimates for the CYP71 clan under site-specific models.

Model	lnl	Estimates of parameters		df(Δnp)	LRTs	P-value	BEB Positive selection sites (*: P>95%; **: P>99%)
		Frequency	ω(dN/dS)				
M0	-229786.164778	p=1.000000	0.228830	4(M3 vs M0)	9715.17	0.00	Not allowed
M3	-224928.578423	p0=0.15193	0.057060				Not allowed
		p1=0.43118	0.203650				
		p2=0.41689	0.441810				
M1a	-227961.211837	p0=0.78574	0.240360	2(M2a vs M1a)	0.00	1.00	Not allowed
		p1=0.21426	1.000000				
M2a	-227961.211837	p0=0.78574	0.240360				**1583*,1597****
		p1=0.16322	1.000000				
		p2=0.05104	1.000000				
M7	-225009.370739	p0=0.33333	0.074400	2(M8 vs M7)			Not allowed
		p1=0.33333	0.234630				
		p2=0.33333	0.481490				
M8	-224721.958457	p0=0.29847	0.062910		574.82	0.00	**1583**,1585*,1590*,1597****
		p1=0.29847	0.188930				
		p2=0.29847	0.390070				
		p3=0.10459	1.000000				
