# Supplementary material for: Global identification, structural analysis and expression characterization of cytochrome P450 monooxygenase superfamily in rice
Source: BMC Genomics. 2018 Jan 10;19:35. doi: 10.1186/s12864-017-4425-8 (PMC5764023; doi:10.1186/s12864-017-4425-8)
Supplement: Supplementary file 9 — Log-likelihood values and parameters estimates for the CYP86 clan under site-specific models. (PDF 53 kb) [file 12864_2017_4425_MOESM9_ESM.pdf]

Table S4. Log-likelihood values and parameters estimates for the CYP86 clan under site-specific models.

**Model**	**lnl**	**Estimates of parameters**		**df(Δnp)**	**LRTs**	**P-value**	**BEB positive selection sites (*: P>95%; **: P>99%)**
		**Frequency**	**ω(dN/dS)**				
M0	-53538.505441	p=1.000000	0.132800	4(M3 vs M0)	2730.76	0.00	Not allowed
M3	-52173.125000	p0=0.23077	0.026650				Not allowed
		p1=0.38233	0.120890				
		p2=0.38689	0.314380				
M1a	-53121.122315	p0=0.80413	0.157070	2(M2a vs M1a)	36.34	0.00	Not allowed
		p1=0.19587	1.000000				
M2a	-53102.949929	p0=0.79315	0.160410				**656*,658**,659**,660***
		p1=0.20662	1.000000				
		p2=0.00023	51.301970				
M7	-52187.150018	p0=0.33333	0.029990	2(M8 vs M7)	83.16	0.00	Not allowed
		p1=0.33333	0.126570				
		p2=0.33333	0.315980				
M8	-52145.570038	p0=0.33254	0.030060				**656*,658**,659**,660***
		p1=0.33254	0.125320				
		p2=0.33254	0.311510				
		p3=0.00237	5.993960				
