# Supplementary material for: Global identification, structural analysis and expression characterization of cytochrome P450 monooxygenase superfamily in rice
Source: BMC Genomics. 2018 Jan 10;19:35. doi: 10.1186/s12864-017-4425-8 (PMC5764023; doi:10.1186/s12864-017-4425-8)
Supplement: Supplementary file 10 — Log-likelihood values and parameters estimates for the CYP710 clan under site-specific models. (PDF 53 kb) [file 12864_2017_4425_MOESM10_ESM.pdf]

Table S5. Log-likelihood values and parameters estimates for the CYP710 clan under site-specific models.

Model	lnl	Estimates of parameters		df(Δnp)	LRTs	P-value	BEB positive selection sites (*: P>95%; **: P>99%)
		Frequency	ω(dN/dS)				
M0	-3899.152855	p=1.000000	0.140280	4(M3 vs M0)	88.86	0.00	Not allowed
M3	-3854.721952	p0=0.75440	0.045630				Not allowed
		p1=0.23912	0.472500				
		p2=0.00647	6.013910				
M1a	-3858.805517	p0=0.88795	0.075870	2(M2a vs M1a)	0.00	1.00	Not allowed
		p1=0.11205	1.000000				
M2a	-3858.805517	p0=0.88795	0.075870				None
		p1=0.06790	1.000000				
		p2=0.04415	1.000000				
M7	-3862.579586	p0=0.33333	0.000530	8(M8 vs M7)	25.19	0.00	Not allowed
		p1=0.33333	0.051920				
		p2=0.33333	0.446330				
M8	-3849.984981	p0=0.32760	0.001560				**14*,15*,16***
		p1=0.32760	0.059310				
		p2=0.32760	0.367590				
		p3=0.01720	15.227510				
