# Supplementary material for: Global identification, structural analysis and expression characterization of cytochrome P450 monooxygenase superfamily in rice
Source: BMC Genomics. 2018 Jan 10;19:35. doi: 10.1186/s12864-017-4425-8 (PMC5764023; doi:10.1186/s12864-017-4425-8)
Supplement: Supplementary file 11 — Log-likelihood values and parameters estimates for the CYP72 clan under site-specific models. (PDF 53 kb) [file 12864_2017_4425_MOESM11_ESM.pdf]

Table S6. Log-likelihood values and parameters estimates for the CYP72 clan under site-specific models.

**Model**	**lnl**	**Estimatesofparameters**		**df(Δnp)**	**LRTs**	**P-value**	**BEB positive selection sites(*:P>95%;**:P>99%)**
		**Frequency**	**ω(dN/dS)**				
M0	-44886.736843	p=1.000000	0.179090	4(M3 vs M0)	1902.68	0.00	Notallowed
M3	-43935.397599	p0=0.257140	0.255730				Notallowed
		p1=0.257140	0.255730				
		p2=0.255730	0.468690				
M1a	-44511.435400	p0=0.820180	0.177330	2(M2 vs M1)	0.00	1.00	Notallowed
		p1=0.179820	1.000000				
M2a	-44511.435400	p0=0.820180	0.177330				**None**
		p1=0.128490	1.000000				
		p2=0.051330	1.000000				
M7	-43949.279306	p0=0.333330	0.048660	2(M8 vs M7)	26.99	0.00	Notallowed
		p1=0.333330	0.187720				
		p2=0.333330	0.431180				
M8	-43935.786125	p0=0.322400	0.046650				**124*,232***
		p1=0.322400	0.168450				
		p2=0.322400	0.381720				
		p3=0.032800	1.000000				
