# Supplementary material for: Global identification, structural analysis and expression characterization of cytochrome P450 monooxygenase superfamily in rice
Source: BMC Genomics. 2018 Jan 10;19:35. doi: 10.1186/s12864-017-4425-8 (PMC5764023; doi:10.1186/s12864-017-4425-8)
Supplement: Supplementary file 12 — Log-likelihood values and parameters estimates for the CYP711 clan under site-specific models. (PDF 53 kb) [file 12864_2017_4425_MOESM12_ESM.pdf]

Table S7 Log-likelihood values and parameters estimates for the CYP711 clan under site-specific models.

Model	lnl	Estimatesof parameters		df(Δnp)	LRTs	P-value	BEB positive selection sites(*:P>95%;**:P>99%)
		Frequency	ω(dN/dS)				
M0	-6257.115764	p=1.000000	0.123240	4(M3 vs M0)	136.53	0.00	Not allowed
M3	-6188.850115	p0=0.49668	0.034700				Not allowed
		p1=0.46775	0.237460				
		p2=0.03557	6.702490				
M1a	-6209.161261	p0=0.85680	0.088490	2(M2 vs M1)	0.00	1.00	Not allowed
		p1=0.14320	1.000000				
M2a	-6209.161261	p0=0.85680	0.088490				**None**
		p1=0.08688	1.000000				
		p2=0.05632	1.000000				
M7	-6199.369737	p0=0.33333	0.019600	2(M8 vs M7)			Not allowed
		p1=0.33333	0.115860				
		p2=0.33333	0.335430				
M8	-6188.980284	p0=0.32195	0.021090		20.78	0.00	**466***
		p1=0.32195	0.103430				
		p2=0.32195	0.280650				
		p3=0.03416	7.009780				
