# Supplementary material for: Global identification, structural analysis and expression characterization of cytochrome P450 monooxygenase superfamily in rice
Source: BMC Genomics. 2018 Jan 10;19:35. doi: 10.1186/s12864-017-4425-8 (PMC5764023; doi:10.1186/s12864-017-4425-8)
Supplement: Supplementary file 13 — List of the positively selected sites detected by DATAMONKEY. SLAC: Single likelihood ancestor counting, FEL: fixed-effects likelihood, REL: random-effects likelihood (posterior probabilities more than 0.95; Bayes factors >50). (PDF 53 kb) [file 12864_2017_4425_MOESM13_ESM.pdf]

Table S8. List of the positively selected sites detected by DATAMONKEY. SLAC: Single likelihood ancestor counting, FEL: fixed-effects likelihood, REL: random-effects likelihood (posterior probabilities more than 0.95; Bayes factors > 50).

Clan	SLAC	FEL	REL
**CYP51**	None	231,327	None
**CYP71**	None	142,236,243,273,385,400,403,404,406,422,430,487,490,509,539,548,603,645,1011,1145,1151,1204,**1597**,1654	Not allowed
**CYP72**	None	206,663	None
**CYP74**	None	None	None
**CYP85**	**196**	**196**,229,231,271,386	**196**,204,211,213,225,386
**CYP86**	624	69,71,97,**136**,152,347,361,522,541	**136**
**CYP710**	None	None	13,**14**,**15**,**16**,17,18,398
**CYP711**	None	None	224,352,461
**CYP97**	None	67,75,124,126,132	None

Bold font represent the codons identified by at least two methods under positive selection
