# Supplementary material for: Global identification, structural analysis and expression characterization of cytochrome P450 monooxygenase superfamily in rice
Source: BMC Genomics. 2018 Jan 10;19:35. doi: 10.1186/s12864-017-4425-8 (PMC5764023; doi:10.1186/s12864-017-4425-8)
Supplement: Supplementary file 14 — Scatter plot of dN/dS ratio of 230 homologous gene pairs. The letters N, S, T on the right legend signify No duplication, Segmental duplication and Tandem duplication of the pairs, respectively. The different colors stand for different clans. (PDF 403 kb) [file 12864_2017_4425_MOESM14_ESM.pdf]

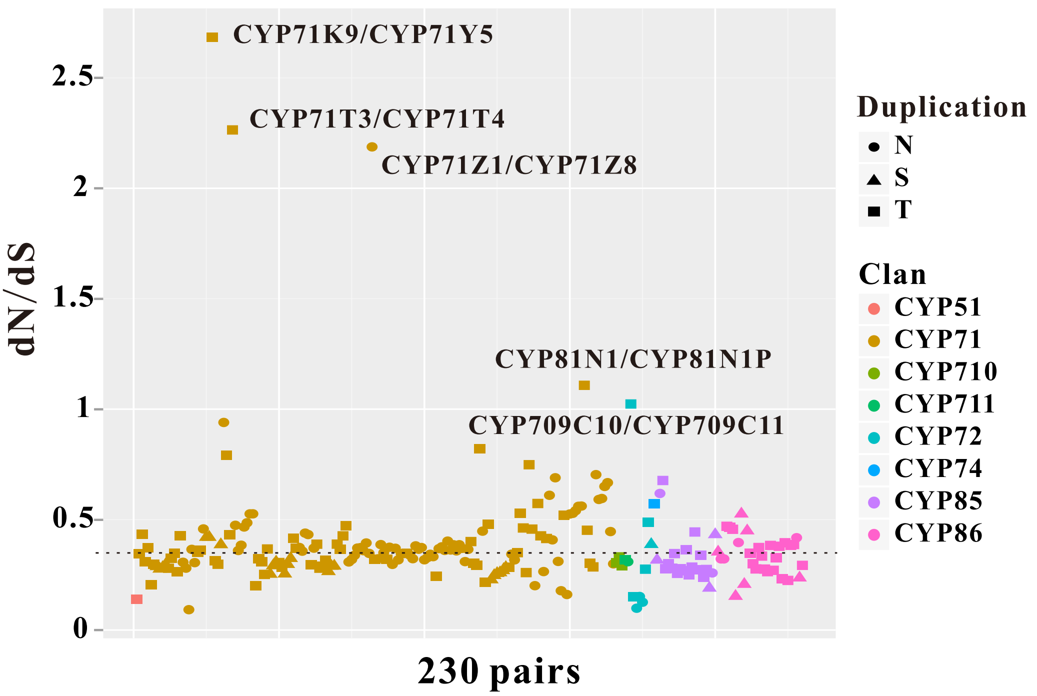


**Figure S6.** Scatter plot of dN/dS ratio of 230 homologous gene pairs. The letters N, S, T on the right legend signify No duplication, Segmental duplication and Tandem duplication of the pairs, respectively. The different colors stand for different clans.
