# Supplementary material for: Global identification, structural analysis and expression characterization of cytochrome P450 monooxygenase superfamily in rice
Source: BMC Genomics. 2018 Jan 10;19:35. doi: 10.1186/s12864-017-4425-8 (PMC5764023; doi:10.1186/s12864-017-4425-8)
Supplement: Supplementary file 16 — List of predicted miRNA-OsCYP pairs with target regions and assessing results. (PDF 137 kb) [file 12864_2017_4425_MOESM16_ESM.pdf]

Table S10. List of predicted miRNA-OsCYP pairs with target regions and assessing results.

miRNA	Target Gene	Score	Mismatches	Gap	G:U wobble	Target Region	Alignment
osa-miR5809 (20 nt)	CYP51G1 (2311 nt)	70	4	1	2	534 - 554	osa-miR5809: 3' CGACACCAG-CGGCCGCUGCU 5'<br>CYP51G1: 5' GCUGAGCUCCGCCGGCGGCGG 3'
osa-miR1848 (21 nt)	CYP51G3 (1719 nt)	86	3	0	2	1326 - 1345	osa-miR1848: 3' ACGUGCGCGCGCGGCCGCUCC 5'<br>CYP51G3: 5' GGCACGCGUGCGUGGGCGAGG 3'
osa-miR5075 (21 nt)	CYP51G3 (1719 nt)	78	3	1	2	773 - 792	osa-miR5075: 3' CGCCUGCCGCCGCUGCCUCUU 5'<br>CYP51G3: 5' GCGG-CGGCGGCGGCGGCGGA 3'
osa-miR5526 (21 nt)	CYP51G3 (1719 nt)	75	3.5	2	3	1593 - 1613	osa-miR5526: 3' GAGUAUGGACUG-AGAUGGAAA 5'<br>CYP51G3: 5' CUCAUAUCU-AUCUCUAUCUUU 3'
osa-miR1848 (21 nt)	CYP51G4P (663 nt)	86	3	0	2	630 - 649	osa-miR1848: 3' ACGUGCGCGCGCGGCCGCUCC 5'<br>CYP51G4P: 5' GGCACGCCUGCGUCGGCGAGG 3'
osa-miR2930 (22 nt)	CYP51G4P (663 nt)	73	3.5	2	1	562 - 581	osa-miR2930: 3' CCGGUGCGCGCUCUCUUCUCUU 5'<br>CYP51G4P: 5' GGCC-C-CGCGAGGGAAGAGUA 3'
osa-miR531b (20 nt)	CYP51G4P (663 nt)	73	3.5	1	1	628 - 648	osa-miR531b: 3' GCCGUGCGU-CGGGGCCGCUC 5'<br>CYP51G4P: 5' CGGCACGCCUGCGUCGGCGAG 3'
osa-miR2118b (22 nt)	CYP51H1 (1485 nt)	72	4	2	4	554 - 573	osa-miR2118b: 3' AUCCUUACCCUCCGUAGCCCUU 5'<br>CYP51H1: 5' UGGGAA-GGGAGGUG-CGGGAG 3'
osa-miR2118n (22 nt)	CYP51H1 (1485 nt)	72	4	2	4	554 - 573	osa-miR2118n: 3' AUCCUUACCCUCCGUAGCCCUU 5'<br>CYP51H1: 5' UGGGAA-GGGAGGUG-CGGGAG 3'
osa-miR1440b (21 nt)	CYP51H6 (1914 nt)	70	3.5	2	3	1740 - 1758	osa-miR1440b: 3' GAGUUUAUGGUGAGAGGAUUU 5'<br>CYP51H6: 5' CU-AGAUAU-ACUCUUCUAAA 3'
osa-miR2866 (20 nt)	CYP51H6 (1914 nt)	70	3.5	2	3	606 - 625	osa-miR2866: 3' CUACGACUU-GUGUUUGAUCU 5'<br>CYP51H6: 5' GAU-CUGAAACAUGAACUGGA 3'
osa-miR1440b (21 nt)	CYP51H7P (5253 nt)	77	4	0	4	4311 - 4331	osa-miR1440b: 3' GAGUUUAUGGUGAGAGGAUUU 5'<br>CYP51H7P: 5' CUCCAAUACCACGUUUUUGAA 3'
osa-miR5075 (21 nt)	CYP51H9 (1506 nt)	81	3	1	2	124 - 142	osa-miR5075: 3' CGCCUGCCGCCGCUGCCUCUU 5'<br>CYP51H9: 5' GCGG-CGGCGGCGGCGGAGGC 3'
osa-miR5075 (21 nt)	CYP51H9 (1506 nt)	78	3	1	2	121 - 140	osa-miR5075: 3' CGCCUGCCGCCGCUGCCUCUU 5'<br>CYP51H9: 5' GCGG-CGGCGGCGGCGGCGGA 3'
osa-miR531b (20 nt)	CYP51H9 (1506 nt)	81	3	1	2	1322 - 1341	osa-miR531b: 3' GCCGUGCGU-CGGGGCCGCUC 5'<br>CYP51H9: 5' AGGCACGCGUGCCUCGGCGAG 3'
osa-miR5075 (21 nt)	CYP701A6v1 (1961 nt)	72	4	2	4	126 - 146	osa-miR5075: 3' CGCCUGCCGCCGCUG-CCUCUU 5'<br>CYP701A6v1: 5' GCGG-CGGCGGCGGUCGGAGGG 3'
osa-miR5075 (21 nt)	CYP701A6v2 (1864 nt)	72	4	2	4	126 - 146	osa-miR5075: 3' CGCCUGCCGCCGCUG-CCUCUU 5'<br>CYP701A6v2: 5' GCGG-CGGCGGCGGUCGGAGGG 3'
osa-miR169r-3p (21 nt)	CYP701A8 (1845 nt)	83	3	1	2	1089 - 1110	osa-miR169r-3p: 3' CCAUCGGCUCCU-CUGAACGGU 5'<br>CYP701A8: 5' GGUGACCGAGGACGACUUGCCG 3'
osa-miR812a (22 nt)	CYP701A8 (1845 nt)	86	4	0	2	1761 - 1780	osa-miR812a: 3' CAGGUUGCAAAUUGGCAGGCAG 5'<br>CYP701A8: 5' CGCCAACGUUUGACCAUUCGUC 3'
osa-miR812b (22 nt)	CYP701A8 (1845 nt)	86	4	0	2	1761 - 1780	osa-miR812b: 3' CAGGUUGCAAAUUGGCAGGCAG 5'<br>CYP701A8: 5' CGCCAACGUUUGACCAUUCGUC 3'
osa-miR812c (22 nt)	CYP701A8 (1845 nt)	86	4	0	2	1761 - 1780	osa-miR812c: 3' CAGGUUGCAAAUUGGCAGGCAG 5'<br>CYP701A8: 5' CGCCAACGUUUGACCAUUCGUC 3'
osa-miR812d (22 nt)	CYP701A8 (1845 nt)	86	4	0	2	1761 - 1780	osa-miR812d: 3' CAGGUUGCAAAUUGGCAGGCAG 5'<br>CYP701A8: 5' CGCCAACGUUUGACCAUUCGUC 3'
osa-miR812e (22 nt)	CYP701A8 (1845 nt)	86	4	0	2	1761 - 1780	osa-miR812e: 3' CAGGUUGCAAAUUGGCAGGCAG 5'<br>CYP701A8: 5' CGCCAACGUUUGACCAUUCGUC 3'
osa-miR5075 (21 nt)	CYP701A9 (1803 nt)	75	4	1	2	1122 - 1143	osa-miR5075: 3' CGCC-UGCCGCCGCUGCCUCUU 5'<br>CYP701A9: 5' GCGGCAAGGCGGUGACCGAGGA 3'
osa-miR5542 (21 nt)	CYP701A9 (1803 nt)	71	4	0	2	259 - 276	osa-miR5542: 3' UAGAGUACUAUGGAAGAGUUU 5'<br>CYP701A9: 5' GCCUCAUCAUACCUUUACAAA 3'
osa-miR1436 (21 nt)	CYP704A5v1 (2506 nt)	96	1.5	0	3	151 - 171	osa-miR1436: 3' UGAGGGAGGCAGGGUAUUACA 5'<br>CYP704A5v1: 5' ACUCCCUCCGUUUUAUAAUGU 3'
osa-miR5075 (21 nt)	CYP706C1 (2025 nt)	77	4	1	4	1339 - 1360	osa-miR5075: 3' CGCCUGCC-GCCGCUGCCUCUU 5'<br>CYP706C1: 5' GCCGGCGGACGGCGGCGGAGGG 3'
osa-miR531a (24 nt)	CYP706C1 (2025 nt)	90	4	0	2	1193 - 1216	osa-miR531a: 3' UACCGCCGUGCGUCGGGGCCGCUC 5'<br>CYP706C1: 5' AUGGUGCCGCACUGCCCCGGCGAG 3'
osa-miR531c (24 nt)	CYP706C1 (2025 nt)	90	4	0	2	1193 - 1216	osa-miR531c: 3' UACCGCCGUGCGUCGGGGCCGCUC 5'<br>CYP706C1: 5' AUGGUGCCGCACUGCCCCGGCGAG 3'
osa-miR5788 (21 nt)	CYP706C1 (2025 nt)	70	4	2	2	1879 - 1899	osa-miR5788: 3' AU-GAUCUCAUACAGUGUAGGU 5'<br>CYP706C1: 5' UAGCUGGAGAAUGUC-CAUCUA 3'
osa-miR2864.1 (21 nt)	CYP706C2 (1917 nt)	73	4	1	2	970 - 989	osa-miR2864.1: 3' ACGUUUUGUUCC-CGUCGUUUU 5'<br>CYP706C2: 5' UUCUGAACAAGGUGCAGCAAGA 3'
osa-miR5144-5p (21 nt)	CYP706C2 (1917 nt)	80	3.5	2	3	313 - 335	osa-miR5144-5p: 3' CAGAG-AAGUCGUCGUGU-UCUU 5'<br>CYP706C2: 5' GUCUCCUUCGGCGGCGCACAGAA 3'
osa-miR2919 (19 nt)	CYP707A37 (1935 nt)	75	3.5	0	5	10 - 27	osa-miR2919: 3' AGAAAGGGGGGGGGGGGAA 5'<br>CYP707A37: 5' UUUUUCUCCUCCCUCUCUC 3'
osa-miR2919 (19 nt)	CYP707A5v1 (2345 nt)	72	4	0	6	37 - 54	osa-miR2919: 3' AGAAAGGGGGGGGGGGGAA 5'<br>CYP707A5v1: 5' CCUUUCUCUUCCUCUUCUU 3'
osa-miR2925 (19 nt)	CYP707A5v1 (2345 nt)	70	3.5	0	3	166 - 184	osa-miR2925: 3' UGCUUCGGGCGCCGGCGGU 5'<br>CYP707A5v1: 5' GCGCCGUCCGCGGCCGCCG 3'
osa-miR396d (21 nt)	CYP707A5v1 (2345 nt)	70	3.5	2	3	1162 - 1180	osa-miR396d: 3' GGCAAGUUCUUUCGGACACCU 5'<br>CYP707A5v1: 5' CC-UUCAGGGAGGCC-GUGGA 3'
osa-miR396g (21 nt)	CYP707A5v1 (2345 nt)	70	3.5	2	3	1162 - 1180	osa-miR396g: 3' GGCAAGUUCUUUCGGACACCU 5'<br>CYP707A5v1: 5' CC-UUCAGGGAGGCC-GUGGA 3'
osa-miR396h (21 nt)	CYP707A5v1 (2345 nt)	70	3.5	2	3	1162 - 1180	osa-miR396h: 3' GGCAAGUUCUUUCGGACACCU 5'<br>CYP707A5v1: 5' CC-UUCAGGGAGGCC-GUGGA 3'
osa-miR2919 (19 nt)	CYP707A5v2 (2500 nt)	75	3	1	4	1335 - 1354	osa-miR2919: 3' AGA-AAGGGGGGGGGGGGAA 5'<br>CYP707A5v2: 5' UUUGUUCCCUUCCCUCCCUU 3'
osa-miR2919 (19 nt)	CYP707A5v2 (2500 nt)	72	4	0	6	37 - 54	osa-miR2919: 3' AGAAAGGGGGGGGGGGGAA 5'<br>CYP707A5v2: 5' CCUUUCUCUUCCUCUUCUU 3'
osa-miR2925 (19 nt)	CYP707A5v2 (2500 nt)	70	3.5	0	3	166 - 184	osa-miR2925: 3' UGCUUCGGGCGCCGGCGGU 5'<br>CYP707A5v2: 5' GCGCCGUCCGCGGCCGCCG 3'
osa-miR396d (21 nt)	CYP707A5v2 (2500 nt)	70	3.5	2	3	1162 - 1180	osa-miR396d: 3' GGCAAGUUCUUUCGGACACCU 5'<br>CYP707A5v2: 5' CC-UUCAGGGAGGCC-GUGGA 3'
osa-miR396g (21 nt)	CYP707A5v2 (2500 nt)	70	3.5	2	3	1162 - 1180	osa-miR396g: 3' GGCAAGUUCUUUCGGACACCU 5'<br>CYP707A5v2: 5' CC-UUCAGGGAGGCC-GUGGA 3'
osa-miR396h (21 nt)	CYP707A5v2 (2500 nt)	70	3.5	2	3	1162 - 1180	osa-miR396h: 3' GGCAAGUUCUUUCGGACACCU 5'<br>CYP707A5v2: 5' CC-UUCAGGGAGGCC-GUGGA 3'
osa-miR2919 (19 nt)	CYP707A5v3 (1880 nt)	75	3	1	4	715 - 734	osa-miR2919: 3' AGA-AAGGGGGGGGGGGGAA 5'<br>CYP707A5v3: 5' UUUGUUCCCUUCCCUCCCUU 3'
osa-miR396d (21 nt)	CYP707A5v3 (1880 nt)	70	3.5	2	3	542 - 560	osa-miR396d: 3' GGCAAGUUCUUUCGGACACCU 5'<br>CYP707A5v3: 5' CC-UUCAGGGAGGCC-GUGGA 3'
osa-miR396g (21 nt)	CYP707A5v3 (1880 nt)	70	3.5	2	3	542 - 560	osa-miR396g: 3' GGCAAGUUCUUUCGGACACCU 5'<br>CYP707A5v3: 5' CC-UUCAGGGAGGCC-GUGGA 3'
osa-miR396h (21 nt)	CYP707A5v3 (1880 nt)	70	3.5	2	3	542 - 560	osa-miR396h: 3' GGCAAGUUCUUUCGGACACCU 5'<br>CYP707A5v3: 5' CC-UUCAGGGAGGCC-GUGGA 3'
osa-miR414 (21 nt)	CYP707A6 (2199 nt)	74	4	1	0	1994 - 2012	osa-miR414: 3' CCUGCUACUA-CUACUCCUACU 5'<br>CYP707A6: 5' GUUCGAUGAUCGAUGAUGA 3'
osa-miR529a (20 nt)	CYP707A6 (2199 nt)	75	3.5	0	3	117 - 136	osa-miR529a: 3' CUUCUUCUCUCUCCCAUGUC 5'<br>CYP707A6: 5' GAGAGAGAGAGAGGGCACGG 3'
osa-miR6247 (21 nt)	CYP707A6 (2199 nt)	75	3.5	2	3	2095 - 2115	osa-miR6247: 3' ACGGUAAUCCAGUUA-GUCGGU 5'<br>CYP707A6: 5' UGC-AUUGGGUUAAUACAGCUA 3'
osa-miR2102-5p (20 nt)	CYP709C10 (1614 nt)	73	3.5	1	1	211 - 231	osa-miR2102-5p: 3' CACCGC-CGCCGCCGAACGGG 5'<br>CYP709C10: 5' GCGGCGAGCGGCGGCGUCGUC 3'
osa-miR5075 (21 nt)	CYP709C10 (1614 nt)	81	2.5	1	1	943 - 962	osa-miR5075: 3' CGCCUGCCGCCGCUGCCUCUU 5'<br>CYP709C10: 5' GCGGCGGCGGCGGCGG-AGAA 3'
osa-miR5075 (21 nt)	CYP709C10 (1614 nt)	70	4	2	2	940 - 960	osa-miR5075: 3' CGCCUGCCG-CCGCUGCCUCUU 5'<br>CYP709C10: 5' GCGG-CGGCCGGCGGCGGCGAG 3'
osa-miR164d (21 nt)	CYP709C5 (2285 nt)	73	4	1	2	1531 - 1550	osa-miR164d: 3' UCGUGCA-CGGGACGAAGAGGU 5'<br>CYP709C5: 5' AACGCGUUGCCUCCUCUUCCA 3'
osa-miR5523 (22 nt)	CYP709C5 (2285 nt)	91	2.5	0	1	1298 - 1319	osa-miR5523: 3' GAUCAUUUAUACAAGGAGGAGU 5'<br>CYP709C5: 5' CUAGUCAACAUGUUCCUCCUCG 3'
osa-miR5525 (22 nt)	CYP709C5 (2285 nt)	75	4	2	2	62 - 83	osa-miR5525: 3' AAGUCUAGCGAGGGUUCCA-AGU 5'<br>CYP709C5: 5' UUCG-AUCUCUCUCAAGGUCUCA 3'
osa-miR164c (21 nt)	CYP709C6 (1898 nt)	73	4	1	2	1458 - 1476	osa-miR164c: 3' ACGUGCAUGGGACGAAGAGGU 5'<br>CYP709C6: 5' CGCAC-UACUCCUCCUCUCUA 3'
osa-miR419 (21 nt)	CYP709C6 (1898 nt)	79	4	0	6	148 - 168	osa-miR419: 3' GUUGUAGCAGUCGUAAGUAGU 5'<br>CYP709C6: 5' CGGCGUCGUGGGCGUCAUCG 3'
osa-miR5523 (22 nt)	CYP709C6 (1898 nt)	82	4	0	4	1224 - 1245	osa-miR5523: 3' GAUCAUUUAUACAAGGAGGAGU 5'<br>CYP709C6: 5' UUGGUCAACAUGUUCCUCCUCA 3'
osa-miR164c (21 nt)	CYP709C8 (1865 nt)	73	4	1	2	1517 - 1535	osa-miR164c: 3' ACGUGCAUGGGACGAAGAGGU 5'<br>CYP709C8: 5' CGCAC-UACUCUCCCUCUCUA 3'
osa-miR164c (21 nt)	CYP709C9 (2009 nt)	76	3.5	1	1	1471 - 1489	osa-miR164c: 3' ACGUGCAUGGGACGAAGAGGU 5'<br>CYP709C9: 5' CGCAC-UACUCUCCCUCUCCA 3'
osa-miR2925 (19 nt)	CYP709D1 (1807 nt)	70	3.5	0	3	212 - 230	osa-miR2925: 3' UGCUUCGGGCGCCGGCGGU 5'<br>CYP709D1: 5' AUGAAGGCGCCGCCGUCGCG 3'
osa-miR5543 (21 nt)	CYP709D1 (1807 nt)	76	3	1	2	790 - 808	osa-miR5543: 3' GUUCUUUAUAUGGUAAGUAU 5'<br>CYP709D1: 5' UAAGGAAAU-UACCAUCAUA 3'
osa-miR1439 (21 nt)	CYP710A5 (1809 nt)	83	3	0	2	1767 - 1787	osa-miR1439: 3' UUAUGAGUGAGGCAAGGUUUU 5'<br>CYP710A5: 5' AGUACUCCCUCCGUUUGAAAA 3'
osa-miR166c-5p (20 nt)	CYP711A2 (1236 nt)	70	4	1	4	94 - 111	osa-miR166c-5p: 3' GAGCCUGGUCUGUUGUAAGG 5'<br>CYP711A2: 5' CUCGGGC-AGGCAGCGUCG 3'
osa-miR166d-5p (21 nt)	CYP711A2 (1236 nt)	75	4	1	4	93 - 111	osa-miR166d-5p: 3' GGAGCUCGGUCUGUUGUAAGG 5'<br>CYP711A2: 5' CCUCGGGC-AGGCAGCGUCG 3'
osa-miR5144-5p (21 nt)	CYP711A2 (1236 nt)	73	4	0	2	449 - 467	osa-miR5144-5p: 3' CAGAGAAGUCGUCGUGUUCUU 5'<br>CYP711A2: 5' GGCGCUGCAGCAGCGCAAGGA 3'
osa-miR413 (21 nt)	CYP711A3 (1158 nt)	72	4	2	4	783 - 803	osa-miR413: 3' CUGCGCCUUGUCA-CUUGAUCG 5'<br>CYP711A3: 5' GUGCG-AACAGGUUGAAAUUG 3'
osa-miR166b-5p (21 nt)	CYP711A4 (1551 nt)	75	3.5	2	3	1334 - 1354	osa-miR166b-5p: 3' GGGGCU-CGGUCUGUUGUAAGG 5'<br>CYP711A4: 5' UCCCGGAGCCAGA-AAUAUCC 3'
osa-miR419 (21 nt)	CYP711A4 (1551 nt)	78	4	0	4	658 - 676	osa-miR419: 3' GUUGUAGCAGUCGUAAGUAGU 5'<br>CYP711A4: 5' AGGCAUCGUCGGAGUCAUCA 3'
osa-miR2925 (19 nt)	CYP711A5v1 (2029 nt)	72	3.5	0	5	1572 - 1590	osa-miR2925: 3' UGCUUCGGGCGCCGGCGGU 5'<br>CYP711A5v1: 5' GUGAAGCUCGCCGCCGUCG 3'
osa-miR5075 (21 nt)	CYP711A5v1 (2029 nt)	78	3	1	2	1470 - 1489	osa-miR5075: 3' CGCCUGCCGCCGCUGCCUCUU 5'<br>CYP711A5v1: 5' GCGG-CGGGGGCGGCGGAGGA 3'
osa-miR5075 (21 nt)	CYP711A5v1 (2029 nt)	75	3.5	1	3	1473 - 1492	osa-miR5075: 3' CGCCUGCCGCCGCUGCCUCUU 5'<br>CYP711A5v1: 5' GCGGG-GGCGGCGGAGGAGG 3'
osa-miR5075 (21 nt)	CYP711A5v1 (2029 nt)	71	4	1	2	988 - 1005	osa-miR5075: 3' CGCCUGCCGCCGCUGCCUCUU 5'<br>CYP711A5v1: 5' GCG-ACGCCGGCGACGGGGCG 3'
osa-miR2925 (19 nt)	CYP711A5v2 (1627 nt)	72	3.5	0	5	1170 - 1188	osa-miR2925: 3' UGCUUCGGGCGCCGGCGGU 5'<br>CYP711A5v2: 5' GUGAAGCUCGCCGCCGUCG 3'
osa-miR5075 (21 nt)	CYP711A5v2 (1627 nt)	78	3	1	2	1068 - 1087	osa-miR5075: 3' CGCCUGCCGCCGCUGCCUCUU 5'<br>CYP711A5v2: 5' GCGG-CGGGGGCGGCGGAGGA 3'
osa-miR5075 (21 nt)	CYP711A5v2 (1627 nt)	75	3.5	1	3	1071 - 1090	osa-miR5075: 3' CGCCUGCCGCCGCUGCCUCUU 5'<br>CYP711A5v2: 5' GCGGG-GGCGGCGGAGGAGG 3'
osa-miR5075 (21 nt)	CYP711A5v2 (1627 nt)	71	4	1	2	586 - 603	osa-miR5075: 3' CGCCUGCCGCCGCUGCCUCUU 5'<br>CYP711A5v2: 5' GCG-ACGCCGGCGACGGGGCG 3'
osa-miR1858a (21 nt)	CYP714B1 (1754 nt)	75	4	0	2	166 - 186	osa-miR1858a: 3' CGGGGUGAGGCAGGAGGAGAG 5'<br>CYP714B1: 5' GCCCAACCCGUCCUUCCCUU 3'
osa-miR1858b (21 nt)	CYP714B1 (1754 nt)	75	4	0	2	166 - 186	osa-miR1858b: 3' CGGGGUGAGGCAGGAGGAGAG 5'<br>CYP714B1: 5' GCCCAACCCGUCCUUCCCUU 3'
osa-miR5075 (21 nt)	CYP714B1 (1754 nt)	75	3.5	1	3	958 - 977	osa-miR5075: 3' CGCCUGCCGCCGCUGCCUCUU 5'<br>CYP714B1: 5' GCGG-CGGUGGCGGCGGCGAG 3'
osa-miR2105 (20 nt)	CYP714C2 (2264 nt)	70	4	1	2	348 - 368	osa-miR2105: 3' UACUUAG-UAAGUGUAGUGUU 5'<br>CYP714C2: 5' AUGGAGCUAUUCUCAUCACAG 3'
osa-miR815a (21 nt)	CYP714C2 (2264 nt)	75	4	1	4	17 - 35	osa-miR815a: 3' GGGUUAGAGGAGUUAGGGGAA 5'<br>CYP714C2: 5' GUCAGUUUCCUCA-UCCUCUU 3'
osa-miR815b (21 nt)	CYP714C2 (2264 nt)	75	4	1	4	17 - 35	osa-miR815b: 3' GGGUUAGAGGAGUUAGGGGAA 5'<br>CYP714C2: 5' GUCAGUUUCCUCA-UCCUCUU 3'
osa-miR815c (21 nt)	CYP714C2 (2264 nt)	75	4	1	4	17 - 35	osa-miR815c: 3' GGGUUAGAGGAGUUAGGGGAA 5'<br>CYP714C2: 5' GUCAGUUUCCUCA-UCCUCUU 3'
osa-miR5809 (20 nt)	CYP714D1 (2030 nt)	70	4	0	2	846 - 865	osa-miR5809: 3' CGACACCAGCGGCCGCUGCU 5'<br>CYP714D1: 5' GCGGAGGUCGUCGUCGACGG 3'
osa-miR5075 (21 nt)	CYP715B1 (1596 nt)	75	4	0	2	1228 - 1248	osa-miR5075: 3' CGCCUGCCGCCGCUGCCUCUU 5'<br>CYP715B1: 5' GUGGUCGCCGGCGACGGCGAG 3'
osa-miR5809 (20 nt)	CYP715B1 (1596 nt)	86	2	0	2	1225 - 1244	osa-miR5809: 3' CGACACCAGCGGCCGCUGCU 5'<br>CYP715B1: 5' GUGGUGGUCGCCGGCGACGG 3'
osa-miR395x (21 nt)	CYP71AB3 (2108 nt)	75	4	0	2	87 - 107	osa-miR395x: 3' CUCGAUGAGGUUUGUGAAGUG 5'<br>CYP71AB3: 5' GUGCUGCGCCAAAAGCUUCAC 3'
osa-miR2931 (20 nt)	CYP71AC3P (558 nt)	76	3.5	2	1	379 - 399	osa-miR2931: 3' AAAAC-UGUA-GUUGUUAUUUC 5'<br>CYP71AC3P: 5' AUUUGCACAUGCAACAAUAGAG 3'
osa-miR168a-5p (21 nt)	CYP71AD1 (1934 nt)	77	4	0	4	1527 - 1547	osa-miR168a-5p: 3' CAGGGCUAGACGUGGUUCGCU 5'<br>CYP71AD1: 5' GUCCCGUUCGGCGGCCGGGCGG 3'
osa-miR5075 (21 nt)	CYP71AD1 (1934 nt)	70	4	1	2	687 - 706	osa-miR5075: 3' CGCCUGCCGCCGCUGCCUCUU 5'<br>CYP71AD1: 5' GCGGCCG-CGGCGGCGGUGGA 3'
osa-miR1858a (21 nt)	CYP71AK2 (1850 nt)	75	4	0	2	47 - 67	osa-miR1858a: 3' CGGGGUGAGGCAGGAGGAGAG 5'<br>CYP71AK2: 5' GUACCACUACGUCUUCCUCGC 3'
osa-miR1858b (21 nt)	CYP71AK2 (1850 nt)	75	4	0	2	47 - 67	osa-miR1858b: 3' CGGGGUGAGGCAGGAGGAGAG 5'<br>CYP71AK2: 5' GUACCACUACGUCUUCCUCGC 3'
osa-miR166g-5p (21 nt)	CYP71C15 (1713 nt)	71	4	1	0	1627 - 1645	osa-miR166g-5p: 3' CUAGAACUAGACGGAGGUAA 5'<br>CYP71C15: 5' CAUGU-GGAUCACCCUCCAUU 3'
osa-miR1848 (21 nt)	CYP71C15 (1713 nt)	73	4	1	0	190 - 211	osa-miR1848: 3' ACGUGCG-CGCGCGGCCGCUCC 5'<br>CYP71C15: 5' UCCUCGCCGCGCGCCGCCGAGG 3'
osa-miR2104 (22 nt)	CYP71C15 (1713 nt)	80	4	1	2	177 - 199	osa-miR2104: 3' GUGCGAGCGU-AGGGGAGCGGCG 5'<br>CYP71C15: 5' CACCCUCGUCGUCUCCUCGCCGC 3'
osa-miR408-3p (21 nt)	CYP71C15 (1713 nt)	80	3.5	0	3	391 - 411	osa-miR408-3p: 3' CGGUCCCUUCGUCACGUC 5'<br>CYP71C15: 5' GCCCGGGAGGAGGAGGUGCGG 3'
osa-miR5496 (21 nt)	CYP71C15 (1713 nt)	75	4	1	2	715 - 736	osa-miR5496: 3' CUCUUGAUACGGUGG-CCGACC 5'<br>CYP71C15: 5' GAUGACCAUGCCACCAGGUUGG 3'
osa-miR5821 (21 nt)	CYP71C15 (1713 nt)	76	3.5	2	1	156 - 175	osa-miR5821: 3' GCGGGUGGUAGC-GAGGCAGGU 5'<br>CYP71C15: 5' CGUCCACC-UCGGCUCCGUCCC 3'
osa-miR2919 (19 nt)	CYP71C16 (1575 nt)	70	4	1	4	62 - 80	osa-miR2919: 3' AGAA-AGGGGGGGGGGGGAA 5'<br>CYP71C16: 5' UCUUCUUCCUCCUCUCCUCUC 3'
osa-miR408-3p (21 nt)	CYP71C16 (1575 nt)	77	4	0	4	508 - 528	osa-miR408-3p: 3' CGGUCCCUUCGUCACGUC 5'<br>CYP71C16: 5' GUCGGGAGGAGGAGGUGCGG 3'
osa-miR5075 (21 nt)	CYP71C16 (1575 nt)	78	3	1	2	559 - 578	osa-miR5075: 3' CGCCUGCCGCCGCUGCCUCUU 5'<br>CYP71C16: 5' GCGG-CGGCGGCGGCGGUGGA 3'
osa-miR5821 (21 nt)	CYP71C16 (1575 nt)	73	4	2	2	273 - 292	osa-miR5821: 3' GCGGGUGGUAGC-GAGGCAGGU 5'<br>CYP71C16: 5' CGUCCGCC-UCGGCUCCGUCCC 3'
osa-miR444f (21 nt)	CYP71C16 (1575 nt)	76	4	0	0	17 - 36	osa-miR444f: 3' UUCGAACUCCGUUGUUGACGU 5'<br>CYP71C16: 5' CAGCUAGAAGCAAAAACUGCA 3'
osa-miR2105 (20 nt)	CYP71C20 (1542 nt)	70	3.5	2	3	296 - 315	osa-miR2105: 3' UACU-UAGUAAGUGUAGUGUU 5'<br>CYP71C20: 5' AUGACAU-AUUCGCAUCACGG 3'
osa-miR396e-5p (21 nt)	CYP71C32 (1931 nt)	70	4	1	2	446 - 465	osa-miR396e-5p: 3' GUCAAGUUCUUUCGGACACCU 5'<br>CYP71C32: 5' CAGG-CAAGGAAGCUUGUGAA 3'
osa-miR2926 (20 nt)	CYP71E4 (1978 nt)	73	4	0	2	1115 - 1133	osa-miR2926: 3' UCGUGGUGCAGCUGCUGGA 5'<br>CYP71E4: 5' AGGGCCACCGUCGGCGACCG 3'
osa-miR5819 (21 nt)	CYP71E5 (1935 nt)	75	4	0	4	190 - 209	osa-miR5819: 3' GCGGCGGCAAGGGGAGCAGGA 5'<br>CYP71E5: 5' UGUCAUCGUUUCCCUCGUGCU 3'
osa-miR2925 (19 nt)	CYP71K1 (1831 nt)	72	3.5	0	5	1448 - 1466	osa-miR2925: 3' UGCUUCGGGCGCCGGCGGU 5'<br>CYP71K1: 5' AUGGAGCUCGCGCUGCCG 3'
osa-miR5077 (19 nt)	CYP71K1 (1831 nt)	71	3	1	2	607 - 623	osa-miR5077: 3' ACCACUUGGGCUGCGCUUG 5'<br>CYP71K1: 5' CGGUGAACCUGA-GCGAGC 3'
osa-miR2098-5p (20 nt)	CYP71K10 (1515 nt)	73	3	1	2	1103 - 1121	osa-miR2098-5p: 3' GUAGCCGACGGAGGUGCCCU 5'<br>CYP71K10: 5' CAUUG-CUGCUACCACGGGA 3'
osa-miR1846e (20 nt)	CYP71K11 (1861 nt)	74	3.5	0	1	342 - 358	osa-miR1846e: 3' ACCAGGGCCGGAGGAGCAAC 5'<br>CYP71K11: 5' UUAUCCUGGCCUCCUCGUUG 3'
osa-miR1846e (20 nt)	CYP71K12 (1913 nt)	74	3.5	0	1	347 - 363	osa-miR1846e: 3' ACCAGGGCCGGAGGAGCAAC 5'<br>CYP71K12: 5' UCAUCCUGGCCUCCUCGGUG 3'
osa-miR5075 (21 nt)	CYP71K4 (1728 nt)	78	3	1	2	101 - 120	osa-miR5075: 3' CGCCUGCCGCCGCUGCCUCUU 5'<br>CYP71K4: 5' GCGG-CGGCGGCGGCGGCGAG 3'
osa-miR5075 (21 nt)	CYP71K4 (1728 nt)	75	3.5	2	3	104 - 124	osa-miR5075: 3' CGCCUGCCGCCGCUGC-CUCUU 5'<br>CYP71K4: 5' GCGG-CGGCGGCGGCGAGGGGA 3'
osa-miR5079a (22 nt)	CYP71K4 (1728 nt)	82	4	0	4	1067 - 1088	osa-miR5079a: 3' UAUGGUUUUAUUGUCUAGGUUU 5'<br>CYP71K4: 5' AUGACAAGGUGACAGAGCCAAA 3'
osa-miR5079b (22 nt)	CYP71K4 (1728 nt)	82	4	0	4	1067 - 1088	osa-miR5079b: 3' UAUGGUUUUAUUGUCUAGGUUU 5'<br>CYP71K4: 5' AUGACAAGGUGACAGAGCCAAA 3'
osa-miR5528 (21 nt)	CYP71K4 (1728 nt)	76	3	2	0	918 - 938	osa-miR5528: 3' CCG-UUGUAGAUUUUGGCAGAA 5'<br>CYP71K4: 5' GGCUAACAUC-AAAACCGUCAU 3'
osa-miR5809 (20 nt)	CYP71K4 (1728 nt)	70	4	1	2	959 - 979	osa-miR5809: 3' CGACAC-CAGCGGCCGCUGCU 5'<br>CYP71K4: 5' GCAGCGAGUCGUCGGCGACGG 3'
osa-miR5809 (20 nt)	CYP71K6 (1515 nt)	70	3.5	1	3	508 - 526	osa-miR5809: 3' CGACACCAGCGGCCGCUGCU 5'<br>CYP71K6: 5' GCGGUGG-CGUCGGCGGCGG 3'
osa-miR1848 (21 nt)	CYP71K9 (1748 nt)	70	4	1	2	146 - 165	osa-miR1848: 3' ACGUGCGCGCGCGGCCGCUCC 5'<br>CYP71K9: 5' UGGGUG-GCGCGCGCCGCGG 3'
osa-miR168a-5p (21 nt)	CYP71P1 (2172 nt)	72	4	1	4	1417 - 1436	osa-miR168a-5p: 3' CAGGGCUAGACGUGGUUCGCU 5'<br>CYP71P1: 5' GUUCCGG-CUGCACCCGGCGG 3'
osa-miR1850.2 (21 nt)	CYP71P1 (2172 nt)	81	2.5	1	1	234 - 253	osa-miR1850.2: 3' GGUGCAAAUCAAGUGUGUGUU 5'<br>CYP71P1: 5' CCACGGUUAGUUCAUAC-CAA 3'
osa-miR5075 (21 nt)	CYP71P1 (2172 nt)	70	4	1	2	1593 - 1612	osa-miR5075: 3' CGCCUGCCGCCGCUGCCUCUU 5'<br>CYP71P1: 5' GCGC-CGGCGGCGGCGGCGAG 3'
osa-miR2871a-3p (21 nt)	CYP71Q2 (663 nt)	73	3.5	2	1	343 - 363	osa-miR2871a-3p: 3' CACUGGUA-UCUUUGAUUUUAU 5'<br>CYP71Q2: 5' GUG-CCAUCAGAAACAAGAAUA 3'
osa-miR2871b (21 nt)	CYP71Q2 (663 nt)	73	3.5	2	1	343 - 363	osa-miR2871b: 3' CACUGGUA-UCUUUGAUUUUAU 5'<br>CYP71Q2: 5' GUG-CCAUCAGAAACAAGAAUA 3'
osa-miR5075 (21 nt)	CYP71R1 (2396 nt)	81	2.5	1	1	855 - 874	osa-miR5075: 3' CGCCUGCCGCCGCUGCCUCUU 5'<br>CYP71R1: 5' GCGG-CGGCGGCGACGGCGAG 3'
osa-miR440 (24 nt)	CYP71S1 (1733 nt)	86	4	1	2	1130 - 1150	osa-miR440: 3' AACAGGGCUAGUAGU-CCUCUGUGA 5'<br>CYP71S1: 5' UUCGCCUGGUCAUCAAGGAGACACU 3'
osa-miR5542 (21 nt)	CYP71S1 (1733 nt)	85	3	0	4	1687 - 1707	osa-miR5542: 3' UAGAGUACUAUGGAAGAGUUU 5'<br>CYP71S1: 5' AUCUUGAGAUACUUUCUCAAG 3'
osa-miR5801 (24 nt)	CYP71S1 (1733 nt)	85	4	1	2	855 - 877	osa-miR5801: 3' AGGUUGCUAGCUUUUGCUAAACCA 5'<br>CYP71S1: 5' UCCGACGAA-GAAGACGACUUGGU 3'
osa-miR5818 (21 nt)	CYP71S1 (1733 nt)	70	4	1	2	1449 - 1468	osa-miR5818: 3' UUGGACCGGGAAGAUCAAGCU 5'<br>CYP71S1: 5' AACCUC-CUCUUCUGCUUCGA 3'
osa-miR5801 (24 nt)	CYP71S2 (1767 nt)	85	4	1	2	799 - 821	osa-miR5801: 3' AGGUUGCUAGCUUUUGCUAAACCA 5'<br>CYP71S2: 5' UCCGACGAG-GAAGACGACUUGGU 3'
osa-miR5818 (21 nt)	CYP71S2 (1767 nt)	70	4	1	2	1417 - 1436	osa-miR5818: 3' UUGGACCGGGAAGAUCAAGCU 5'<br>CYP71S2: 5' AACCUC-CUCUUCUGCUUCGA 3'
osa-miR160a-3p (21 nt)	CYP71T1 (2005 nt)	72	4	1	0	190 - 205	osa-miR160a-3p: 3' GUACGAACCGAGGAACGUGCG 5'<br>CYP71T1: 5' GCUUCUUGGCUCCUUGC-CGC 3'
osa-miR160b-3p (21 nt)	CYP71T1 (2005 nt)	72	4	1	0	190 - 205	osa-miR160b-3p: 3' GUACGAACCGAGGAACGUGCG 5'<br>CYP71T1: 5' GCUUCUUGGCUCCUUGC-CGC 3'
osa-miR5075 (21 nt)	CYP71T1 (2005 nt)	78	3.5	0	1	943 - 963	osa-miR5075: 3' CGCCUGCCGCCGCUGCCUCUU 5'<br>CYP71T1: 5' GAGGGCGCCGGCGCCGGAGAA 3'
osa-miR2866 (20 nt)	CYP71T2 (1890 nt)	74	4	0	6	921 - 940	osa-miR2866: 3' CUACGACUUGUGUUUGAUCU 5'<br>CYP71T2: 5' GAUGCUGGACGUGAGCGAGG 3'
osa-miR5075 (21 nt)	CYP71T2 (1890 nt)	75	4	0	2	952 - 972	osa-miR5075: 3' CGCCUGCCGCCGCUGCCUCUU 5'<br>CYP71T2: 5' GCCGGCGCCGGCGCCGGAGGA 3'
osa-miR5075 (21 nt)	CYP71T2 (1890 nt)	72	4	1	4	1418 - 1437	osa-miR5075: 3' CGCCUGCCGCCGCUGCCUCUU 5'<br>CYP71T2: 5' GCGG-CGGUGGCGGCGGUGGAG 3'
osa-miR5075 (21 nt)	CYP71T2 (1890 nt)	70	4	1	2	548 - 567	osa-miR5075: 3' CGCCUGCCGCCGCUGCCUCUU 5'<br>CYP71T2: 5' GCUG-CGGCGGCGGCGGCGAG 3'
osa-miR397a (21 nt)	CYP71T3 (1735 nt)	73	4	0	2	1214 - 1232	osa-miR397a: 3' GUAGUUGCGACGUGAGUUACU 5'<br>CYP71T3: 5' CCUCAAGGCUGUACUUAAGGA 3'
osa-miR5075 (21 nt)	CYP71T4 (1850 nt)	75	3.5	1	3	705 - 724	osa-miR5075: 3' CGCCUGCCGCCGCUGCCUCUU 5'<br>CYP71T4: 5' GCGG-CGGCGGCGACAGGGGG 3'
osa-miR1848 (21 nt)	CYP71T8 (1449 nt)	76	4	0	0	384 - 403	osa-miR1848: 3' ACGUGCGCGCGCGGCCGCUCC 5'<br>CYP71T8: 5' CGCCCGCCCGCGCGCCGCGAGG 3'
osa-miR444b.2 (21 nt)	CYP71U3 (3700 nt)	75	4	1	2	2143 - 2164	osa-miR444b.2: 3' UUCGAACUCU-GUUGUUGACGU 5'<br>CYP71U3: 5' AAGCUUCAGAACAAAGGCUGCA 3'
osa-miR444c.2 (21 nt)	CYP71U3 (3700 nt)	75	4	1	2	2143 - 2164	osa-miR444c.2: 3' UUCGAACUCU-GUUGUUGACGU 5'<br>CYP71U3: 5' AAGCUUCAGAACAAAGGCUGCA 3'
osa-miR1848 (21 nt)	CYP71V3 (1542 nt)	75	4	0	2	245 - 265	osa-miR1848: 3' ACGUGCGCGCGCGGCCGCUCC 5'<br>CYP71V3: 5' UGCACCUCCGCGUCGGCCAGG 3'
osa-miR1846c-3p (21 nt)	CYP71V4 (1762 nt)	75	4	1	2	670 - 691	osa-miR1846c-3p: 3' GGUCGCU-CGUCUGGCCCCAGU 5'<br>CYP71V4: 5' UCAGCUGUGAAGACCGGGGUCA 3'
osa-miR2930 (22 nt)	CYP71V4 (1762 nt)	77	4	1	4	1086 - 1106	osa-miR2930: 3' CCGGUGCGCGCUCUCUUCUCUU 5'<br>CYP71V4: 5' GGCAACG-GUGGGAGAGGAGGA 3'
osa-miR168b (21 nt)	CYP71W1 (1629 nt)	70	4	1	2	1415 - 1434	osa-miR168b: 3' AAGGGCUCGACGUGGUUCGGA 5'<br>CYP71W1: 5' UUCCCGA-CGGCAUCAAAUCU 3'
osa-miR7692-3p (24 nt)	CYP71W1 (1629 nt)	87	4	1	4	1140 - 1162	osa-miR7692-3p: 3' CACGGUGCAUUCGUACGGUUCAGU 5'<br>CYP71W1: 5' GUGCCGCGAGA-CGUGCCAGGUCA 3'
osa-miR1848 (21 nt)	CYP71W4 (1820 nt)	73	3.5	1	1	255 - 274	osa-miR1848: 3' ACGUGCGCGCGCGGCCGCUCC 5'<br>CYP71W4: 5' UGCGCGAGCUCGCCGG-GAGG 3'
osa-miR535-3p (21 nt)	CYP71W4 (1820 nt)	70	4	1	2	807 - 826	osa-miR535-3p: 3' UCACUGUUGCCCUCUUUCGUG 5'<br>CYP71W4: 5' AGUG-CAACCGGAGAGUGCGC 3'
osa-miR5809 (20 nt)	CYP71W4 (1820 nt)	81	2.5	2	1	132 - 153	osa-miR5809: 3' CGAC-ACCAGCGGCCGCUG-CU 5'<br>CYP71W4: 5' GCUGCUGGUCGCCGGCGGCAGA 3'
osa-miR5528 (21 nt)	CYP71X10 (1737 nt)	75	3.5	1	3	906 - 925	osa-miR5528: 3' CCGUUGUAGAUUUUGGCAGAA 5'<br>CYP71X10: 5' GGCAAUAUC-AAAGCCGUUAU 3'
osa-miR169r-3p (21 nt)	CYP71X11 (1658 nt)	75	4	2	2	1062 - 1084	osa-miR169r-3p: 3' CCAUCGGCUCCU-CUGAAC-GGU 5'<br>CYP71X11: 5' GGUGACCGAGGAAGACUUGACCG 3'
osa-miR5075 (21 nt)	CYP71X11 (1658 nt)	70	4	1	2	802 - 821	osa-miR5075: 3' CGCCUGCCGCCGCUGCCUCUU 5'<br>CYP71X11: 5' GCGG-CGGCAGCGGCGGCGAG 3'
osa-miR2925 (19 nt)	CYP71X14 (1729 nt)	73	3.5	0	3	756 - 773	[illegible]
osa-miR5528 (21 nt)	CYP71X14 (1729 nt)	78	3	1	2	974 - 993	[illegible]
osa-miR5075 (21 nt)	CYP71X1P (861 nt)	79	4	0	2	83 - 100	[illegible]
osa-miR426 (21 nt)	CYP71X2 (1620 nt)	80	3.5	0	3	1563 - 1583	[illegible]
osa-miR5819 (21 nt)	CYP71X3 (1437 nt)	75	3.5	2	3	33 - 53	[illegible]
osa-miR1436 (21 nt)	CYP71X4 (1720 nt)	70	4	1	4	1578 - 1596	[illegible]
osa-miR531a (24 nt)	CYP71X4 (1720 nt)	88	4	3	0	732 - 758	[illegible]
osa-miR531b (20 nt)	CYP71X4 (1720 nt)	76	3	2	0	737 - 758	[illegible]
osa-miR531c (24 nt)	CYP71X4 (1720 nt)	88	4	3	0	732 - 758	[illegible]
osa-miR164a (21 nt)	CYP71X7 (1818 nt)	73	4	2	2	1201 - 1220	[illegible]
osa-miR164b (21 nt)	CYP71X7 (1818 nt)	73	4	2	2	1201 - 1220	[illegible]
osa-miR164f (21 nt)	CYP71X7 (1818 nt)	73	4	2	2	1201 - 1220	[illegible]
osa-miR5528 (21 nt)	CYP71X8 (1684 nt)	78	3	1	2	900 - 919	[illegible]
osa-miR5795 (22 nt)	CYP71Y1P (1689 nt)	82	4	0	4	1292 - 1313	[illegible]
osa-miR5795 (22 nt)	CYP71Y3 (1563 nt)	82	4	0	4	1160 - 1181	[illegible]
osa-miR5809 (20 nt)	CYP71Y3 (1563 nt)	73	3	1	2	794 - 812	[illegible]
osa-miR5795 (22 nt)	CYP71Y4 (1627 nt)	82	4	0	4	1142 - 1163	[illegible]
osa-miR5809 (20 nt)	CYP71Y4 (1627 nt)	73	3.5	1	1	908 - 928	[illegible]
osa-miR394 (20 nt)	CYP71Y5 (1586 nt)	70	4	1	2	1463 - 1483	[illegible]
osa-miR2925 (19 nt)	CYP71Y7 (1858 nt)	73	3	0	2	542 - 560	[illegible]
osa-miR531b (20 nt)	CYP71Z2 (1850 nt)	72	4	0	4	224 - 243	[illegible]
osa-miR5075 (21 nt)	CYP71Z21 (2068 nt)	80	3	1	4	881 - 900	[illegible]
osa-miR5075 (21 nt)	CYP71Z22 (1548 nt)	76	4	1	2	813 - 830	[illegible]
osa-miR5075 (21 nt)	CYP71Z3 (1569 nt)	73	4	1	2	269 - 287	[illegible]
osa-miR1848 (21 nt)	CYP71Z4 (1676 nt)	70	4	1	2	431 - 450	[illegible]
osa-miR5075 (21 nt)	CYP71Z4 (1676 nt)	73	3.5	1	1	809 - 828	[illegible]
osa-miR5075 (21 nt)	CYP71Z6 (1766 nt)	77	4	0	4	266 - 286	[illegible]
osa-miR1858a (21 nt)	CYP71Z7 (1890 nt)	72	4	1	4	109 - 128	[illegible]
osa-miR1858b (21 nt)	CYP71Z7 (1890 nt)	72	4	1	4	109 - 128	[illegible]
osa-miR5075 (21 nt)	CYP71Z7 (1890 nt)	77	4	0	4	321 - 341	[illegible]
osa-miR1861g (22 nt)	CYP721B2 (1816 nt)	83	4	2	2	1558 - 1580	[illegible]
osa-miR5809 (20 nt)	CYP722B1 (2050 nt)	78	3	1	2	865 - 885	[illegible]
osa-miR2104 (22 nt)	CYP723A2 (1443 nt)	80	4	1	2	1026 - 1048	[illegible]
osa-miR2926 (20 nt)	CYP723A3 (1455 nt)	73	4	0	2	341 - 359	[illegible]
osa-miR5800 (21 nt)	CYP723A3 (1455 nt)	70	4	1	2	1075 - 1094	[illegible]
osa-miR2866 (20 nt)	CYP724B1 (2065 nt)	78	3.5	1	3	38 - 57	[illegible]
osa-miR398b (21 nt)	CYP728A1 (1464 nt)	77	4	0	4	768 - 788	[illegible]
osa-miR5809 (20 nt)	CYP728A1 (1464 nt)	71	3	1	0	350 - 368	[illegible]
osa-miR1848 (21 nt)	CYP728B1 (1841 nt)	73	3.5	1	1	971 - 990	[illegible]
osa-miR439a (21 nt)	CYP728B1 (1841 nt)	80	3.5	0	3	1481 - 1501	[illegible]
osa-miR439b (21 nt)	CYP728B1 (1841 nt)	80	3.5	0	3	1481 - 1501	[illegible]
osa-miR439c (21 nt)	CYP728B1 (1841 nt)	80	3.5	0	3	1481 - 1501	[illegible]
osa-miR439d (21 nt)	CYP728B1 (1841 nt)	80	3.5	0	3	1481 - 1501	[illegible]
osa-miR439e (21 nt)	CYP728B1 (1841 nt)	80	3.5	0	3	1481 - 1501	[illegible]
osa-miR439f (21 nt)	CYP728B1 (1841 nt)	80	3.5	0	3	1481 - 1501	[illegible]
osa-miR439g (21 nt)	CYP728B1 (1841 nt)	80	3.5	0	3	1481 - 1501	[illegible]
osa-miR439h (21 nt)	CYP728B1 (1841 nt)	80	3.5	0	3	1481 - 1501	[illegible]
osa-miR439i (21 nt)	CYP728B1 (1841 nt)	80	3.5	0	3	1481 - 1501	[illegible]
osa-miR5075 (21 nt)	CYP728B1 (1841 nt)	73	3.5	1	1	976 - 995	[illegible]
osa-miR5837.2 (21 nt)	CYP728B1 (1841 nt)	75	3.5	1	3	797 - 816	[illegible]
osa-miR2096-5p (21 nt)	CYP728B3 (1873 nt)	75	4	0	4	921 - 940	[illegible]
osa-miR2118f (22 nt)	CYP728C7 (1416 nt)	75	4	1	2	783 - 803	[illegible]
osa-miR2118j (22 nt)	CYP728C7 (1416 nt)	75	4	1	2	783 - 803	[illegible]
osa-miR2118m (22 nt)	CYP728C7 (1416 nt)	75	4	1	2	783 - 803	[illegible]
osa-miR2919 (19 nt)	CYP728C7 (1416 nt)	70	4	0	4	48 - 65	[illegible]
osa-miR5819 (21 nt)	CYP728C7 (1416 nt)	73	4	1	4	44 - 62	[illegible]
osa-miR5071 (21 nt)	CYP728C9v1 (1931 nt)	77	4	1	4	263 - 284	[illegible]
osa-miR5071 (21 nt)	CYP728C9v2 (2440 nt)	77	4	1	4	263 - 284	[illegible]
osa-miR2876-5p (21 nt)	CYP72A17v1 (2115 nt)	70	4	3	2	705 - 726	[illegible]
osa-miR2876-5p (21 nt)	CYP72A17v2 (2212 nt)	70	4	3	2	802 - 823	[illegible]
osa-miR395u (21 nt)	CYP72A17v2 (2212 nt)	79	3.5	1	1	75 - 92	[illegible]
osa-miR529b (21 nt)	CYP72A18 (2447 nt)	77	4	1	4	1764 - 1785	[illegible]
osa-miR5542 (21 nt)	CYP72A18 (2447 nt)	70	4	1	2	2013 - 2032	[illegible]
osa-miR5788 (21 nt)	CYP72A18 (2447 nt)	70	4	1	2	776 - 795	[illegible]
osa-miR5075 (21 nt)	CYP72A23 (2130 nt)	83	2.5	1	3	295 - 314	[illegible]
osa-miR5075 (21 nt)	CYP72A23 (2130 nt)	75	3.5	1	3	292 - 311	[illegible]
osa-miR816 (20 nt)	CYP72A24 (2060 nt)	71	3.5	1	1	1967 - 1984	[illegible]
osa-miR5075 (21 nt)	CYP72A32 (1608 nt)	73	3.5	2	1	129 - 149	[illegible]
osa-miR5075 (21 nt)	CYP72A33 (1659 nt)	73	3.5	2	1	102 - 122	[illegible]
osa-miR820a (21 nt)	CYP72A33 (1659 nt)	73	3.5	1	1	445 - 464	[illegible]
osa-miR820b (21 nt)	CYP72A33 (1659 nt)	73	3.5	1	1	445 - 464	[illegible]
osa-miR820c (21 nt)	CYP72A33 (1659 nt)	73	3.5	1	1	445 - 464	[illegible]
osa-miR2907a (22 nt)	CYP72A34 (1870 nt)	73	4	4	0	263 - 286	[illegible]
osa-miR2907b (22 nt)	CYP72A34 (1870 nt)	73	4	4	0	263 - 286	[illegible]
osa-miR2907c (22 nt)	CYP72A34 (1870 nt)	73	4	4	0	263 - 286	[illegible]
osa-miR2907d (22 nt)	CYP72A34 (1870 nt)	73	4	4	0	263 - 286	[illegible]
osa-miR6248 (21 nt)	CYP72A35 (2102 nt)	89	2	0	0	1978 - 1998	[illegible]
osa-miR812f (24 nt)	CYP72A35 (2102 nt)	97	3.5	0	5	2017 - 2040	[illegible]
osa-miR3979-5p (20 nt)	CYP733A1 (1780 nt)	73	3.5	1	1	241 - 261	[illegible]
osa-miR5809 (20 nt)	CYP733A1 (1780 nt)	76	3	1	0	1063 - 1083	[illegible]
osa-miR5075 (21 nt)	CYP734A4 (2151 nt)	70	4	1	2	939 - 958	[illegible]
osa-miR1848 (21 nt)	CYP734A5 (1614 nt)	86	3	0	2	597 - 616	[illegible]
osa-miR1848 (21 nt)	CYP734A5 (1614 nt)	81	2.5	1	1	83 - 102	[illegible]
osa-miR167a-5p (21 nt)	CYP734A6 (2143 nt)	70	4	1	2	398 - 417	[illegible]
osa-miR167b (21 nt)	CYP734A6 (2143 nt)	70	4	1	2	398 - 417	[illegible]
osa-miR167c-5p (21 nt)	CYP734A6 (2143 nt)	70	4	1	2	398 - 417	[illegible]
osa-miR5832 (21 nt)	CYP734A6 (2143 nt)	70	4	2	2	1207 - 1227	[illegible]
osa-miR1846b-5p (21 nt)	CYP735A3 (1605 nt)	78	4	0	2	430 - 449	[illegible]
osa-miR1846c-5p (21 nt)	CYP735A3 (1605 nt)	78	4	0	2	430 - 449	[illegible]
osa-miR531b (20 nt)	CYP735A3 (1605 nt)	73	3	2	2	1375 - 1394	[illegible]
osa-miR5075 (21 nt)	CYP73A35P (1446 nt)	70	4	2	2	1152 - 1172	[illegible]
osa-miR156k (21 nt)	CYP73A39 (1993 nt)	78	3.5	1	1	1929 - 1950	[illegible]
osa-miR2102-3p (22 nt)	CYP73A39 (1993 nt)	87	3.5	1	5	285 - 307	[illegible]
osa-miR2927 (23 nt)	CYP73A39 (1993 nt)	80	4	1	2	1096 - 1117	[illegible]
osa-miR2102-3p (22 nt)	CYP73A40 (1914 nt)	87	3.5	1	5	263 - 285	[illegible]
osa-miR2927 (23 nt)	CYP73A40 (1914 nt)	80	4	1	2	1074 - 1095	[illegible]
osa-miR2930 (22 nt)	CYP74E1 (1721 nt)	71	4	2	0	115 - 133	[illegible]
osa-miR2926 (20 nt)	CYP74F1 (1808 nt)	73	4	0	2	571 - 589	[illegible]
osa-miR5809 (20 nt)	CYP74F1 (1808 nt)	76	3.5	1	2	1241 - 1260	[illegible]
osa-miR2925 (19 nt)	CYP75A11 (1996 nt)	75	3	1	4	532 - 551	[illegible]
osa-miR394 (20 nt)	CYP75B11 (2124 nt)	74	4	0	6	798 - 817	[illegible]
osa-miR2099-3p (21 nt)	CYP76H4 (1787 nt)	70	4	1	2	923 - 943	[illegible]
osa-miR5074 (21 nt)	CYP76H6 (2968 nt)	70	4	2	2	261 - 281	[illegible]
osa-miR5074 (21 nt)	CYP76H6 (2968 nt)	70	4	2	2	1272 - 1292	[illegible]
osa-miR1848 (21 nt)	CYP76L1 (2036 nt)	78	3	1	2	207 - 226	[illegible]
osa-miR1848 (21 nt)	CYP76L1 (2036 nt)	76	4	3	0	538 - 560	[illegible]
osa-miR1858a (21 nt)	CYP76L1 (2036 nt)	73	4	2	0	156 - 178	[illegible]
osa-miR1858b (21 nt)	CYP76L1 (2036 nt)	73	4	2	0	156 - 178	[illegible]
osa-miR5075 (21 nt)	CYP76L1 (2036 nt)	70	4	1	2	545 - 564	[illegible]
osa-miR1848 (21 nt)	CYP76M1 (1710 nt)	75	4	0	2	510 - 530	[illegible]
osa-miR160e-3p (21 nt)	CYP76M10 (1668 nt)	78	4	1	2	201 - 221	[illegible]
osa-miR531b (20 nt)	CYP76M13 (2243 nt)	78	3	0	2	774 - 793	[illegible]
osa-miR531b (20 nt)	CYP76M13 (2243 nt)	73	3	2	2	792 - 811	[illegible]
osa-miR1858a (21 nt)	CYP76M14 (1938 nt)	73	4	1	2	160 - 178	[illegible]
osa-miR1858b (21 nt)	CYP76M14 (1938 nt)	73	4	1	2	160 - 178	[illegible]
osa-miR1848 (21 nt)	CYP76M17 (1727 nt)	86	2.5	0	1	527 - 547	[illegible]
osa-miR1848 (21 nt)	CYP76M4Pv1 (2143 nt)	71	4	3	0	1092 - 1112	[illegible]
osa-miR5832 (21 nt)	CYP76M4Pv1 (2143 nt)	70	3.5	2	3	1241 - 1259	[illegible]
osa-miR1848 (21 nt)	CYP76M4Pv2 (2787 nt)	71	4	3	0	1736 - 1756	[illegible]
osa-miR5832 (21 nt)	CYP76M4Pv2 (2787 nt)	70	3.5	2	3	1885 - 1903	[illegible]
osa-miR1848 (21 nt)	CYP76M4Pv3 (2061 nt)	71	4	3	0	1092 - 1112	[illegible]
osa-miR5832 (21 nt)	CYP76M4Pv3 (2061 nt)	70	3.5	2	3	1241 - 1259	[illegible]
osa-miR437 (21 nt)	CYP76M5 (1716 nt)	85	3	0	4	1679 - 1699	[illegible]
osa-miR1848 (21 nt)	CYP76M7 (1742 nt)	86	2.5	0	1	547 - 567	[illegible]
osa-miR2102-3p (22 nt)	CYP76M7 (1742 nt)	84	4	0	6	953 - 974	[illegible]
osa-miR1848 (21 nt)	CYP76M8 (1809 nt)	78	3.5	0	1	590 - 610	[illegible]
osa-miR2102-3p (22 nt)	CYP76M8 (1809 nt)	84	4	0	6	996 - 1017	[illegible]
osa-miR7693-3p (22 nt)	CYP76P2 (2293 nt)	75	4	1	2	1334 - 1354	[illegible]
osa-miR5082 (24 nt)	CYP76P3 (762 nt)	87	4	2	4	419 - 442	[illegible]
osa-miR5075 (21 nt)	CYP76Q2 (1878 nt)	86	2	1	2	396 - 415	[illegible]
osa-miR5075 (21 nt)	CYP76Q2 (1878 nt)	77	4	0	4	392 - 412	[illegible]
osa-miR2924 (21 nt)	CYP76V1 (1703 nt)	78	3.5	0	1	135 - 155	[illegible]
osa-miR444d.3 (21 nt)	CYP76V1 (1703 nt)	79	4	0	6	1100 - 1120	[illegible]
osa-miR1427 (21 nt)	CYP77B2 (2372 nt)	70	4	1	2	1759 - 1778	[illegible]
osa-miR5809 (20 nt)	CYP77B2 (2372 nt)	70	3.5	2	3	499 - 518	[illegible]
osa-miR2104 (22 nt)	CYP78A11 (1668 nt)	78	3.5	1	1	532 - 552	[illegible]
osa-miR531b (20 nt)	CYP78A12 (1771 nt)	72	4	1	4	1242 - 1262	[illegible]
osa-miR395f (21 nt)	CYP78A13 (1945 nt)	75	3.5	1	3	1827 - 1846	[illegible]
osa-miR5819 (21 nt)	CYP78A13 (1945 nt)	75	4	0	2	952 - 972	[illegible]
osa-miR2925 (19 nt)	CYP78A15 (2233 nt)	75	3	0	4	708 - 726	[illegible]
osa-miR171e-5p (21 nt)	CYP78A16 (1725 nt)	70	4	1	2	38 - 57	[illegible]
osa-miR2926 (20 nt)	CYP78A17 (1548 nt)	74	4	0	2	1176 - 1192	[illegible]
osa-miR1848 (21 nt)	CYP79A7 (1904 nt)	73	3.5	2	1	268 - 288	[illegible]
osa-miR5492 (21 nt)	CYP79A9 (3346 nt)	75	4	1	2	3009 - 3030	[illegible]
osa-miR812o-5p (21 nt)	CYP79A9 (3346 nt)	91	2	0	2	731 - 751	[illegible]
osa-miR5492 (21 nt)	CYP79A9P (1642 nt)	75	4	1	2	792 - 813	[illegible]
osa-miR5075 (21 nt)	CYP81A6 (2545 nt)	70	4	2	2	1470 - 1490	[illegible]
osa-miR5075 (21 nt)	CYP81L3 (1925 nt)	83	2.5	1	3	148 - 167	[illegible]
osa-miR5075 (21 nt)	CYP81L5 (1578 nt)	75	4	0	2	858 - 878	[illegible]
osa-miR5509 (21 nt)	CYP81L5 (1578 nt)	73	4	1	2	1509 - 1527	[illegible]
osa-miR2925 (19 nt)	CYP81M1 (1857 nt)	70	3.5	0	3	529 - 547	[illegible]
osa-miR5821 (21 nt)	CYP81M1 (1857 nt)	72	4	2	4	443 - 463	[illegible]
osa-miR5809 (20 nt)	CYP81N1P (1802 nt)	70	4	0	2	116 - 135	[illegible]
osa-miR1858a (21 nt)	CYP81P1 (1764 nt)	78	3	2	2	544 - 564	[illegible]
osa-miR1858b (21 nt)	CYP81P1 (1764 nt)	78	3	2	2	544 - 564	[illegible]
osa-miR2919 (19 nt)	CYP81P1 (1764 nt)	70	4	0	4	70 - 87	[illegible]
osa-miR5819 (21 nt)	CYP81P1 (1764 nt)	75	4	0	2	64 - 84	[illegible]
osa-miR6249a (21 nt)	CYP81P1 (1764 nt)	86	2.5	0	1	320 - 340	[illegible]
osa-miR6249b (21 nt)	CYP81P1 (1764 nt)	86	2.5	0	1	320 - 340	[illegible]
osa-miR531b (20 nt)	CYP84A6 (1717 nt)	73	3	1	2	597 - 615	[illegible]
osa-miR168b (21 nt)	CYP84A7 (1721 nt)	83	3.5	0	3	629 - 648	[illegible]
osa-miR5075 (21 nt)	CYP85A1 (2005 nt)	72	4	1	4	251 - 270	[illegible]
osa-miR1848 (21 nt)	CYP86A11 (1843 nt)	83	3.5	0	3	1535 - 1554	[illegible]
osa-miR5075 (21 nt)	CYP86B3 (2420 nt)	80	3	1	4	1014 - 1033	[illegible]
osa-miR5075 (21 nt)	CYP86B3 (2420 nt)	76	3.5	1	3	164 - 181	[illegible]
osa-miR5075 (21 nt)	CYP86B3 (2420 nt)	75	3.5	1	3	1011 - 1030	[illegible]
osa-miR1848 (21 nt)	CYP86E1v1 (2207 nt)	70	4	2	2	221 - 241	[illegible]
osa-miR1848 (21 nt)	CYP86E1v2 (2044 nt)	70	4	2	2	202 - 222	[illegible]
osa-miR530-3p (21 nt)	CYP87A4v1 (1937 nt)	73	4	1	0	549 - 570	[illegible]
osa-miR530-3p (21 nt)	CYP87A4v2 (2026 nt)	73	4	1	0	549 - 570	[illegible]
osa-miR1858a (21 nt)	CYP87A5 (1521 nt)	80	4	1	4	834 - 854	[illegible]
osa-miR1858b (21 nt)	CYP87A5 (1521 nt)	80	4	1	4	834 - 854	[illegible]
osa-miR2104 (22 nt)	CYP87A5 (1521 nt)	80	4	2	2	885 - 908	[illegible]
osa-miR1858a (21 nt)	CYP87A6 (2185 nt)	80	4	1	4	1049 - 1069	[illegible]
osa-miR1858b (21 nt)	CYP87A6 (2185 nt)	80	4	1	4	1049 - 1069	[illegible]
osa-miR6248 (21 nt)	CYP87B4 (1845 nt)	85	3	0	4	1745 - 1765	[illegible]
osa-miR171b (21 nt)	CYP87C2v1 (2001 nt)	71	4	1	0	72 - 90	[illegible]
osa-miR171c-3p (21 nt)	CYP87C2v1 (2001 nt)	71	4	1	0	72 - 90	[illegible]
osa-miR171d-3p (21 nt)	CYP87C2v1 (2001 nt)	71	4	1	0	72 - 90	[illegible]
osa-miR171e-3p (21 nt)	CYP87C2v1 (2001 nt)	71	4	1	0	72 - 90	[illegible]
osa-miR171f-3p (21 nt)	CYP87C2v1 (2001 nt)	71	4	1	0	72 - 90	[illegible]
osa-miR5491 (21 nt)	CYP87C2v1 (2001 nt)	78	4	1	2	1977 - 1997	[illegible]
osa-miR171b (21 nt)	CYP87C2v2 (2019 nt)	71	4	1	0	72 - 90	[illegible]
osa-miR171c-3p (21 nt)	CYP87C2v2 (2019 nt)	71	4	1	0	72 - 90	[illegible]
osa-miR171d-3p (21 nt)	CYP87C2v2 (2019 nt)	71	4	1	0	72 - 90	[illegible]
osa-miR171e-3p (21 nt)	CYP87C2v2 (2019 nt)	71	4	1	0	72 - 90	[illegible]
osa-miR171f-3p (21 nt)	CYP87C2v2 (2019 nt)	71	4	1	0	72 - 90	[illegible]
osa-miR5491 (21 nt)	CYP87C2v2 (2019 nt)	78	4	1	2	1995 - 2015	[illegible]
osa-miR5833 (21 nt)	CYP87C5P (954 nt)	78	4	0	2	379 - 398	[illegible]
osa-miR5496 (21 nt)	CYP88A5 (1937 nt)	75	3.5	2	3	437 - 457	[illegible]
osa-miR5809 (20 nt)	CYP88A5 (1937 nt)	75	4	0	4	119 - 137	[illegible]
osa-miR5075 (21 nt)	CYP89B10 (1602 nt)	88	2.5	0	3	1348 - 1368	[illegible]
osa-miR531b (20 nt)	CYP89B10 (1602 nt)	70	3.5	1	3	284 - 302	[illegible]
osa-miR1848 (21 nt)	CYP89B11 (1856 nt)	76	3.5	1	1	512 - 530	[illegible]
osa-miR2102-3p (22 nt)	CYP89B11 (1856 nt)	85	3.5	1	5	173 - 194	[illegible]
osa-miR2102-5p (20 nt)	CYP89B11 (1856 nt)	76	3	2	0	321 - 342	[illegible]
osa-miR414 (21 nt)	CYP89B11 (1856 nt)	78	3.5	0	1	1423 - 1443	[illegible]
osa-miR5075 (21 nt)	CYP89B11 (1856 nt)	78	4	0	2	1387 - 1406	[illegible]
osa-miR5809 (20 nt)	CYP89B11 (1856 nt)	70	4	0	2	1560 - 1579	[illegible]
osa-miR1439 (21 nt)	CYP89B5P (1269 nt)	81	4	0	2	1222 - 1240	[illegible]
osa-miR5819 (21 nt)	CYP89B5P (1269 nt)	70	4	2	2	183 - 203	[illegible]
osa-miR5075 (21 nt)	CYP89B6 (1589 nt)	78	3.5	2	1	831 - 853	[illegible]
osa-miR2864.1 (21 nt)	CYP89B7P (1179 nt)	75	4	1	2	421 - 442	[illegible]
osa-miR156f-3p (22 nt)	CYP89B8P (1320 nt)	82	3.5	1	5	80 - 100	[illegible]
osa-miR156h-3p (22 nt)	CYP89B8P (1320 nt)	82	3.5	1	5	80 - 100	[illegible]
osa-miR156l-3p (22 nt)	CYP89B8P (1320 nt)	82	3.5	1	5	80 - 100	[illegible]
osa-miR531b (20 nt)	CYP89B9 (1896 nt)	70	4	1	4	352 - 369	[illegible]
osa-miR5075 (21 nt)	CYP89C1 (1810 nt)	70	4	2	2	769 - 789	[illegible]
osa-miR5809 (20 nt)	CYP89C1 (1810 nt)	79	2	1	0	1443 - 1461	[illegible]
osa-miR5075 (21 nt)	CYP89C8P (1404 nt)	70	4	2	2	648 - 668	[illegible]
osa-miR5809 (20 nt)	CYP89C8P (1404 nt)	79	2	1	0	1325 - 1343	[illegible]
osa-miR1436 (21 nt)	CYP89C9 (1955 nt)	85	3	0	4	1669 - 1689	[illegible]
osa-miR5075 (21 nt)	CYP89C9 (1955 nt)	70	4	2	2	753 - 773	[illegible]
osa-miR5809 (20 nt)	CYP89C9 (1955 nt)	76	2.5	1	1	1430 - 1448	[illegible]
osa-miR5821 (21 nt)	CYP89D1 (1575 nt)	80	3.5	0	3	326 - 346	[illegible]
osa-miR5832 (21 nt)	CYP89F1 (1524 nt)	83	3.5	0	3	610 - 629	[illegible]
osa-miR397a (21 nt)	CYP89G1 (1605 nt)	72	4	1	4	614 - 633	[illegible]
osa-miR2104 (22 nt)	CYP90A19 (1987 nt)	70	4	3	2	96 - 116	[illegible]
osa-miR2104 (22 nt)	CYP90A3 (1825 nt)	70	4	3	2	75 - 95	[illegible]
osa-miR5075 (21 nt)	CYP90D2v1 (1473 nt)	75	3.5	1	3	292 - 311	[illegible]
osa-miR5809 (20 nt)	CYP90D3 (1807 nt)	72	4	0	4	267 - 286	[illegible]
osa-miR1437b-3p (24 nt)	CYP92A11 (1866 nt)	85	4	1	2	469 - 491	[illegible]
osa-miR156j-3p (22 nt)	CYP92A12 (1563 nt)	75	4	2	2	1449 - 1470	[illegible]
osa-miR5075 (21 nt)	CYP92A12 (1563 nt)	77	4	1	4	806 - 827	[illegible]
osa-miR6249a (21 nt)	CYP92A14 (1910 nt)	76	3	2	0	431 - 451	[illegible]
osa-miR6249b (21 nt)	CYP92A14 (1910 nt)	76	3	2	0	431 - 451	[illegible]
osa-miR167e-3p (21 nt)	CYP92A15 (1851 nt)	70	4	1	2	637 - 656	[illegible]
osa-miR167i-3p (21 nt)	CYP92A15 (1851 nt)	70	4	1	2	637 - 656	[illegible]
osa-miR5075 (21 nt)	CYP92A15 (1851 nt)	73	3	2	2	1566 - 1584	[illegible]
osa-miR160e-3p (21 nt)	CYP92A8 (5802 nt)	75	3.5	1	3	1134 - 1153	[illegible]
osa-miR1848 (21 nt)	CYP92A9 (1988 nt)	73	3.5	2	1	696 - 716	[illegible]
osa-miR2925 (19 nt)	CYP92C21 (1835 nt)	70	3.5	1	3	184 - 203	[illegible]
osa-miR5809 (20 nt)	CYP92C21 (1835 nt)	76	3.5	0	1	1196 - 1214	[illegible]
osa-miR6249a (21 nt)	CYP92C21 (1835 nt)	94	1.5	0	1	322 - 342	[illegible]
osa-miR6249b (21 nt)	CYP92C21 (1835 nt)	94	1.5	0	1	322 - 342	[illegible]
osa-miR397a (21 nt)	CYP93F1 (1898 nt)	72	4	1	4	600 - 619	[illegible]
osa-miR2102-5p (20 nt)	CYP93G1v1 (1928 nt)	79	3	1	0	135 - 154	[illegible]
osa-miR2924 (21 nt)	CYP93G1v1 (1928 nt)	72	4	1	4	664 - 683	[illegible]
osa-miR5493 (21 nt)	CYP93G1v1 (1928 nt)	71	3.5	1	1	492 - 509	[illegible]
osa-miR2102-5p (20 nt)	CYP93G1v2 (1442 nt)	79	3	1	0	135 - 154	[illegible]
osa-miR2924 (21 nt)	CYP93G1v2 (1442 nt)	72	4	1	4	664 - 683	[illegible]
osa-miR5493 (21 nt)	CYP93G1v2 (1442 nt)	71	3.5	1	1	492 - 509	[illegible]
osa-miR5075 (21 nt)	CYP93G2 (2205 nt)	70	4	1	2	592 - 611	[illegible]
osa-miR5526 (21 nt)	CYP93G2 (2205 nt)	72	4	1	4	1885 - 1904	[illegible]
osa-miR2925 (19 nt)	CYP94B4 (1671 nt)	75	3	0	4	685 - 703	[illegible]
osa-miR5075 (21 nt)	CYP94B4 (1671 nt)	83	3	0	2	252 - 272	[illegible]
osa-miR5075 (21 nt)	CYP94B4 (1671 nt)	78	3	2	2	848 - 868	[illegible]
osa-miR1846d-3p (20 nt)	CYP94B5 (1808 nt)	71	4	0	2	1517 - 1534	[illegible]
osa-miR395x (21 nt)	CYP94B5 (1808 nt)	72	4	1	4	618 - 637	[illegible]
osa-miR5075 (21 nt)	CYP94B5 (1808 nt)	80	3.5	0	3	941 - 961	[illegible]
osa-miR5075 (21 nt)	CYP94B5 (1808 nt)	73	4	1	2	665 - 683	[illegible]
osa-miR5075 (21 nt)	CYP94B5 (1808 nt)	72	4	1	4	1377 - 1396	[illegible]
osa-miR2926 (20 nt)	CYP94C2 (1921 nt)	73	4	0	2	439 - 457	[illegible]
osa-miR2927 (23 nt)	CYP94C2 (1921 nt)	85	4	0	2	918 - 940	[illegible]
osa-miR1431 (21 nt)	CYP94C3v1 (3781 nt)	78	4	1	2	3180 - 3200	[illegible]
osa-miR2097-3p (22 nt)	CYP94C3v1 (3781 nt)	70	4	3	2	2061 - 2081	[illegible]
osa-miR2927 (23 nt)	CYP94C3v1 (3781 nt)	115	0	0	0	1033 - 1055	[illegible]
osa-miR399k (21 nt)	CYP94C3v1 (3781 nt)	75	4	1	2	2550 - 2571	[illegible]
osa-miR1431 (21 nt)	CYP94C3v2 (2846 nt)	78	4	1	2	2245 - 2265	[illegible]
osa-miR2927 (23 nt)	CYP94C3v2 (2846 nt)	115	0	0	0	1033 - 1055	[illegible]
osa-miR1438 (22 nt)	CYP94C4 (2451 nt)	88	3	0	2	2180 - 2201	[illegible]
osa-miR1848 (21 nt)	CYP94C4 (2451 nt)	73	3.5	1	1	974 - 993	[illegible]
osa-miR5542 (21 nt)	CYP94C4 (2451 nt)	77	4	0	4	2164 - 2184	[illegible]
osa-miR439a (21 nt)	CYP94D10 (1536 nt)	72	4	2	4	551 - 571	[illegible]
osa-miR439b (21 nt)	CYP94D10 (1536 nt)	72	4	2	4	551 - 571	[illegible]
osa-miR439c (21 nt)	CYP94D10 (1536 nt)	72	4	2	4	551 - 571	[illegible]
osa-miR439d (21 nt)	CYP94D10 (1536 nt)	72	4	2	4	551 - 571	[illegible]
osa-miR439e (21 nt)	CYP94D10 (1536 nt)	72	4	2	4	551 - 571	[illegible]
osa-miR439f (21 nt)	CYP94D10 (1536 nt)	72	4	2	4	551 - 571	[illegible]
osa-miR439g (21 nt)	CYP94D10 (1536 nt)	72	4	2	4	551 - 571	[illegible]
osa-miR439h (21 nt)	CYP94D10 (1536 nt)	72	4	2	4	551 - 571	[illegible]
osa-miR439i (21 nt)	CYP94D10 (1536 nt)	72	4	2	4	551 - 571	[illegible]
osa-miR5813 (22 nt)	CYP94D11 (1941 nt)	77	4	1	4	1633 - 1653	[illegible]
osa-miR2103 (22 nt)	CYP94D13 (1494 nt)	80	4	1	2	802 - 824	[illegible]
osa-miR2926 (20 nt)	CYP94D15 (2021 nt)	70	4	0	2	865 - 884	[illegible]
osa-miR5534b (21 nt)	CYP94D4 (1548 nt)	82	3.5	0	3	964 - 980	[illegible]
osa-miR5338 (21 nt)	CYP94D5 (2324 nt)	74	4	1	6	1674 - 1693	[illegible]
osa-miR426 (21 nt)	CYP94D6 (1473 nt)	73	3.5	2	1	300 - 320	[illegible]
osa-miR5534b (21 nt)	CYP94D64 (4788 nt)	82	3.5	0	3	964 - 980	[illegible]
osa-miR5819 (21 nt)	CYP94D64 (4788 nt)	78	3	1	2	2550 - 2569	[illegible]
osa-miR2925 (19 nt)	CYP94E1 (1650 nt)	70	3.5	0	3	1477 - 1495	[illegible]
osa-miR5075 (21 nt)	CYP94E1 (1650 nt)	85	3	0	4	1221 - 1241	[illegible]
osa-miR5075 (21 nt)	CYP94E1 (1650 nt)	75	3.5	1	3	1370 - 1389	[illegible]
osa-miR5075 (21 nt)	CYP94E1 (1650 nt)	72	4	1	4	1373 - 1392	[illegible]
osa-miR1428a-5p (21 nt)	CYP94E2 (1828 nt)	72	4	1	4	583 - 602	[illegible]
osa-miR2925 (19 nt)	CYP94E2 (1828 nt)	70	3.5	0	3	1517 - 1535	[illegible]
osa-miR5506 (21 nt)	CYP94E2 (1828 nt)	75	3.5	1	3	1226 - 1245	[illegible]
osa-miR3980a-5p (21 nt)	CYP94E2 (1828 nt)	78	3	1	2	574 - 593	[illegible]
osa-miR3980b-5p (21 nt)	CYP94E2 (1828 nt)	78	3	1	2	574 - 593	[illegible]
osa-miR319a-3p.2-3p (20 nt)	CYP96B2 (1611 nt)	75	4	0	4	1518 - 1536	[illegible]
osa-miR319b (20 nt)	CYP96B2 (1611 nt)	75	4	0	4	1518 - 1536	[illegible]
osa-miR535-3p (21 nt)	CYP96B2 (1611 nt)	78	3.5	1	1	454 - 475	[illegible]
osa-miR531b (20 nt)	CYP96B3 (1879 nt)	73	3.5	1	1	1123 - 1143	[illegible]
osa-miR5077 (19 nt)	CYP96B4 (2104 nt)	70	3.5	1	3	1403 - 1422	[illegible]
osa-miR1427 (21 nt)	CYP96B5 (2135 nt)	76	4	0	2	445 - 462	[illegible]
osa-miR5077 (19 nt)	CYP96B5 (2135 nt)	70	3.5	1	3	1099 - 1118	[illegible]
osa-miR1429-3p (21 nt)	CYP96B6 (1932 nt)	75	4	1	2	225 - 246	[illegible]
osa-miR2925 (19 nt)	CYP96B6 (1932 nt)	76	3	1	2	285 - 303	[illegible]
osa-miR5143a (24 nt)	CYP96B6 (1932 nt)	83	3.5	3	1	213 - 235	[illegible]
osa-miR5143b (24 nt)	CYP96B6 (1932 nt)	83	3.5	3	1	213 - 235	[illegible]
osa-miR528-3p (21 nt)	CYP96B6 (1932 nt)	70	4	2	2	1357 - 1377	[illegible]
osa-miR5795 (22 nt)	CYP96B6 (1932 nt)	80	4	0	2	1516 - 1536	[illegible]
osa-miR166b-5p (21 nt)	CYP96B9 (1524 nt)	70	4	2	2	248 - 268	[illegible]
osa-miR1846c-3p (21 nt)	CYP96D2 (2617 nt)	77	4	1	2	124 - 145	[illegible]
osa-miR1848 (21 nt)	CYP96D2 (2617 nt)	73	4	1	2	684 - 702	[illegible]
osa-miR2868 (20 nt)	CYP96D2 (2617 nt)	72	4	1	4	2274 - 2294	[illegible]
osa-miR531b (20 nt)	CYP96D2 (2617 nt)	70	4	0	2	682 - 701	[illegible]
osa-miR5514 (21 nt)	CYP96E1 (1790 nt)	76	4	1	2	801 - 818	[illegible]
osa-miR1860-5p (21 nt)	CYP97A4v1 (2234 nt)	78	3.5	1	1	672 - 693	[illegible]
osa-miR1860-5p (21 nt)	CYP97A4v2 (2558 nt)	78	3.5	1	1	996 - 1017	[illegible]
osa-miR1860-5p (21 nt)	CYP97A4v3 (2164 nt)	78	3.5	1	1	602 - 623	[illegible]
osa-miR1860-5p (21 nt)	CYP97A4v4 (2473 nt)	78	3.5	1	1	911 - 932	[illegible]
osa-miR1860-5p (21 nt)	CYP97A4v5 (2361 nt)	78	3.5	1	1	996 - 1017	[illegible]
osa-miR1847.1 (21 nt)	CYP98A4v2 (1812 nt)	75	3.5	1	3	204 - 223	[illegible]
osa-miR2275a (22 nt)	CYP99A3 (1818 nt)	75	4	2	2	1216 - 1237	[illegible]
osa-miR2275b (22 nt)	CYP99A3 (1818 nt)	75	4	2	2	1216 - 1237	[illegible]
