# Supplementary material for: Global identification, structural analysis and expression characterization of cytochrome P450 monooxygenase superfamily in rice
Source: BMC Genomics. 2018 Jan 10;19:35. doi: 10.1186/s12864-017-4425-8 (PMC5764023; doi:10.1186/s12864-017-4425-8)
Supplement: Supplementary file 17 — List of primers for qPCR used in this study. (PDF 45 kb) [file 12864_2017_4425_MOESM17_ESM.pdf]

Table S11. List of primers for qPCR used in this study.

CYP name	Locus	sense	anti-sense
CYP74A4	LOC_Os03g55800	5'-CTCTTCACCGGCACCTTCAT-3'	5'-CGGTGGGAGAGGAGGTAGAA-3'
CYP94C79	LOC_Os12g05440	5'-CGCTCACCACTCTGTTCATGAT-3'	5'-TGTAGTTCAGCCGCTTCAGGT-3'
CYP94C2	LOC_Os11g05380	5'-TCAGGAAGGCCATCAAGGTC-3'	5'-GAGGAGGAAGCTGACGACGAT-3'
CYP73A38	LOC_Os05g25640	5'-TCGAGTACAACTACGGTGACTTCAT-3'	5'-ATCTCACCAGTCTGTTCCATCACT-3'
CYP51G3	LOC_Os05g12040	5'-CCGCCACCATTATGAGAAGAT-3'	5'-CCTCTGACCCAACCAAGAAAGT-3'
CYP709C9	LOC_Os07g23570	5'-CTGAGCACGCACCCAGATT-3'	5'-ATAGTGCACCCTCAGGCATCT-3'
CYP709C5	LOC_Os07g44140	5'-CCACCTTCCTTAGCATCCAAAT-3'	5'-GTTCCCGTATCCTGCGACAT-3'
CYP76M2	LOC_Os08g39730	5'-CCGGTGGCACCGATACTACT-3'	5'-CTCGGAAATCTACCTCTGCTCTT-3'
CYP89C9	LOC_Os08g05620	5'-GTTCTGCCTCCTGGTGTACATGT-3'	5'-AAGACCTGGAAGCTGAGGAAGTT-3'
CYP704A5	LOC_Os10g38110	5'-CAAGGAGAGGATCAAGGTCGTC-3'	5'-CGTTGCCTGGATGAATCTTGT-3'
UBQ5	LOC_Os06g44080	5'-ACATTGAAGGTGGCTCAGTGCT-3'	5'-GCCAATCGCATAGGACTTCAAT-3'
