# Supplementary material for: Global identification, structural analysis and expression characterization of cytochrome P450 monooxygenase superfamily in rice
Source: BMC Genomics. 2018 Jan 10;19:35. doi: 10.1186/s12864-017-4425-8 (PMC5764023; doi:10.1186/s12864-017-4425-8)
Supplement: Supplementary file 18 — Annotation of co-expressed genes. (PDF 67 kb) [file 12864_2017_4425_MOESM18_ESM.pdf]

Table S12. Annotation of co-expressed genes.

Names	MSU ID	RAP-DB ID	Hierarchy	Description
CYP87C2	LOC_Os03g45619	Os03g0658800	0	Cytochrome P450 family protein.OsCYP87C2
FLA	LOC_Os02g20560	Os02g0308800	1	Fasciclin domain containing protein, expressed
Os03g22010	LOC_Os03g22010	Os03g0339300	1	Similar to Peroxidase (EC 1.11.1.7).
Os03g31430	LOC_Os03g31430	Os03g0428200	1	Similar to Beta-caryophyllene synthase QHS1.
RALFL30	LOC_Os04g31430	Os04g0383100	1	Rapid ALkalinization Factor RALF family protein precursor.
HKT1	LOC_Os04g51830	Os04g0607600	1	OsHKT1;4 - Na+ transporterrotein.
Os05g43870	LOC_Os05g43870	Os05g0514500	1	myosin heavy chain-related
Os06g03220	LOC_Os06g03220	Os06g0123000	1	Conserved hypothetical protein.
NCED2	LOC_Os12g24800	Os12g0435200	1	9-cis-epoxycarotenoid dioxygenase 1, chloroplast precursor, putative, expressed.
Os01g18120	LOC_Os01g18120	Os01g0283700	2	Similar to Cinnamoyl-CoA reductase (EC 1.2.1.44).
KAT1	LOC_Os01g55200	Os01g0756700	2	Potassium channel KAT1, putative, expressed.
1bglu2	LOC_Os01g59819	Os01g0813700	2	Os1bglu2 - beta-glucosidase homologue, similar to G. max isohydroxyurate hydrolase, expressed.
Os03g16290	LOC_Os03g16290	Os03g0269700	2	Fringe-related protein, putative, expressed.
RALFL20	LOC_Os04g31484	Os04g0384000	2	RALFL20 - Rapid ALkalinization Factor RALF family protein precursor, expressed.
Os04g32860	LOC_Os04g32860	Os04g0401100	2	Heavy metal transport/detoxification protein domain containing protein.
Os05g03972	LOC_Os05g03972	Os05g0130400	2	Protein of unknown function DUF247, plant family protein.
Os05g37600	LOC_Os05g37600	Os05g0448300	2	Glycerol-3-phosphate acyltransferase, putative, expressed.
Os05g45180	LOC_Os05g45180	Os05g0527800	2	Anthocyanidin 5,3-O-glucosyltransferase, putative, expressed.
Os06g21250	LOC_Os06g21250	Os06g0317400	2	Glycine rich protein family protein, putative, expressed.
Os06g21270	LOC_Os06g21270	Os06g0317600	2	Flycine rich protein family protein, putative, expressed.
Os07g41050	LOC_Os07g41050	Os07g0601600	2	NAD dependent epimerase/dehydratase family protein, putative, expressed.
Os09g07290	LOC_Os09g07290	Os09g0247600	2	Lipolytic enzyme, G-D-S-L family protein.
Os09g08720	LOC_Os09g08720	Os09g0262000	2	Similar to Cinnamoyl-CoA reductase (EC 1.2.1.44).
Os09g13650	LOC_Os09g13650	Os09g0307300	2	Microtubule-associated protein, putative, expressed.
Os09g20700	LOC_Os09g20700	Os09g0373300	2	Strictosidine synthase family protein.
Os10g42390	LOC_Os10g42390	Os10g0574400	2	Zinc finger, C3HC4 type domain containing protein, expressed
Os11g26920	LOC_Os11g26920	Os11g0457000	2	UDP-glucuronosyl/UDP-glucosyltransferase family protein.
Os11g42220	LOC_Os11g42220	Os11g0641800	2	Cupredoxin domain containing protein.
MYB.1	LOC_Os12g03150	Os12g0125000	2	Myb factor.
RALFL41	LOC_Os12g38370	Os12g0571800	2	RALFL41 - Rapid ALkalinization Factor RALF family protein precursor, expressed.
Os01g11010	LOC_Os01g11010	Os01g0208400	3	Peptide-N4-asparagine amidase A, putative, expressed.
MYB.2	LOC_Os01g36460	Os01g0545100	3	MYB family transcription factor, putative, expressed.
C3H9	LOC_Os01g45730	Os01g0645000	3	Zinc finger C-x8-C-x5-C-x3-H type family protein, expressed.
esterase PIR7A	LOC_Os01g70830	Os01g0934600	3	Esterase/lipase/thioesterase domain containing protein.
Os02g20540	LOC_Os02g20540	Os02g0308400	3	Beta-Ig-H3/fasciclin domain containing protein.
Os02g45160	LOC_Os02g45160	Os02g0673100	3	Aluminum-activated malate transporter, putative, expressed.
LTPL108	LOC_Os03g14654	Os03g0251000	3	LTPL108 - Protease inhibitor/seed storage/LTP family protein precursor, expressed.
Os03g24510	LOC_Os03g24510	Os03g0359600	3	Glycosyl transferase 8 domain containing protein, putative, expressed.
Os03g42569	LOC_Os03g42569	Os03g0624000	3	Similar to CDPK substrate protein 1.
4bglu10	LOC_Os04g39840	Os04g0474500	3	Beta-glucosidase homologue, similar to Os4Bglu12 exoglucanase/beta-glucosidase, expressed.
HEV2	LOC_Os04g41640	Os04g0493600	3	HEV2 - Hevein family protein precursor, expressed.
Os04g49900	LOC_Os04g49900	Os04g0588700	3	ABC transporter, transmembrane region domain containing protein.
Os05g09704	LOC_Os05g09704	Os05g0189300	3	HAD superfamily phosphatase, putative, expressed.
Os05g10210	LOC_Os05g10210	Os05g0190500	3	HAD superfamily phosphatase, putative, expressed.
POX1	LOC_Os05g41990	Os05g0499300	3	Similar to Peroxidase (EC 1.11.1.7).
Os06g10920	LOC_Os06g10920	Os06g0211700	3	Xyloglucan fucosyltransferase family protein.
Os07g01520	LOC_Os07g01520	Os07g0105800	3	Protein of unknown function DUF594 family protein.
PDC3	LOC_Os07g49250	Os07g0693100	3	Similar to Pyruvate decarboxylase isozyme 3 (EC 4.1.1.1) (PDC).
Os09g04624	LOC_Os09g04624	Os09g0132200	3	Lipolytic enzyme, G-D-S-L family protein.
CYP92A14	LOC_Os09g08990	Os09g0264400	3	Cytochrome P450 family protein.CYP92A14
Os09g39760	LOC_Os09g39760	Os09g0571100	3	Virulence factor, pectin lyase fold family protein.
POEI7	LOC_Os10g05860	Os10g0149200	3	Pollen Ole e I allergen and extensin family protein precursor, putative, expressed.
OSC7	LOC_Os11g08569	Os11g0189600	3	Similar to Cycloartenol synthase.
RALFL24	LOC_Os11g26340	Os11g0449600	3	Rapid ALkalinization Factor RALF family protein precursor, expressed.
SCP60	LOC_Os11g27264	Os11g0460800	3	OsSCP60 - Putative Serine Carboxypeptidase homologue, expressed
Os11g41850	LOC_Os11g41850	Os11g0637200	3	Similar to Sorbitol transporter.
