# Supplementary material for: Global identification, structural analysis and expression characterization of cytochrome P450 monooxygenase superfamily in rice
Source: BMC Genomics. 2018 Jan 10;19:35. doi: 10.1186/s12864-017-4425-8 (PMC5764023; doi:10.1186/s12864-017-4425-8)
Supplement: Supplementary file 1 — The identified OsCYPs and their related information. (PDF 131 kb) [file 12864_2017_4425_MOESM1_ESM.pdf]

Table S1. The identified OsCYPs and their related information.

[1] Predicted the position of the transmembrane helices separated by 'i' if the loop is on the inside or 'o' if it is on the outside.

[2] Predicted subcellular localization of OsCYPs. "Secretory pathway", "chloroplast", "mitochondrion" and "any other location" are represented as "S", "C", "M" and "_", respectively.

CYP name	MSU/TIGR locus ID	RAPDB_Locus ID	Protein ID[2]	W/C	Locus	AA full length	Theoretical pI	Molecular weight (average)	Molecular weight (monoisotopic)	FXXGXRXCXG motif	Position of FXXGXRXCXG motif	transmembrane helices[1]	Subsellular Localization (SignalP)[2]	Subsellular Localization (LocTree3)	Arabidopsis Ortholog	Rice Ortholog
CYP51G1	LOC_Os11g32240	OS11G0525200	LOC_Os11g32240.1	W	19048313..19052882	490	8.63	55,534.09	55,498.77	SFGGGRHGCLG	425 - 434	i12-34o	S	ER	AT1G11680	-
CYP51G3	LOC_Os05g12040	OS05G0211100	LOC_Os05g12040.1	C	6882663..6884876	500	9.38	55,391.52	55,356.56	AFGGGRHACVG	438 - 447	o5-27i	S	ER	-	-
CYP51G4P	LOC_Os07g37980	OS07G0567200	LOC_Os07g37980.1	W	22788931..22791186	220	10.04	24,761.05	24,745.69	VFGGGRHACVG	206-215	o	-	ER	-	-
CYP51H1	LOC_Os07g28160	OS07G0465400	LOC_Os07g28160.1	W	16438501..16442141	494	9.22	55,547.83	55,512.68	AFGAGRHSCAG	430 - 439	o4-21i65-84o	M	ER	-	-
CYP51H4	LOC_Os02g21810	OS02G0323600	LOC_Os02g21810.1	W	12970222..12972230	504	8.41	55,995.95	55,960.26	SFGAGKHACMG	443 - 452	i7-26o	S	ER	-	-
CYP51H5	LOC_Os02g02230	OS02G0113200	LOC_Os02g02230.1	W	712586..715746	526	9.19	58,723.60	58,686.18	PFSAGRHVCLG	421 - 430	o5-22i43-65o	S	ER	-	-
CYP51H6	LOC_Os05g34325	OS05G0415800	LOC_Os05g34325.1	W	20325336..20328408	514	8.64	57,629.58	57,592.70	SFGGGRRNSCVG	449-458	i7-29o	S	ER	-	LOC_Os05g34380
CYP51H7P	LOC_Os05g34330	OS05G0415900	LOC_Os05g34330.1	W	20335505..20351280	1750	8.70	198,927.72	198,802.62	SFGGGRTGCVG	393 - 402	o	C	mitochondrion	-	-
CYP51H8	LOC_Os05g34380	OS05G0416400	LOC_Os05g34380.1	W	20379467..20381121	469	8.83	53,666.11	53,631.69	SFGGGRHICIG	405 - 414	o	M	ER	-	-
CYP51H9	LOC_Os07g37970	OS07G0567100	LOC_Os07g37970.1	W	22785167..22787365	501	9.28	55,595.23	55,559.79	AFGGGRHACLG	437 - 446	o5-27i77-99o	S	ER	-	-
CYP701A19	LOC_Os06g37330	OS06G0569900	LOC_Os06g37330.1	C	22048646..22057757	504	6.23	56,831.41	56,794.78	AFGAGRRVCAG	441 - 450	i7-29o	S	chloroplast	AT5G25900	LOC_Os06g37364
CYP701A6v1	LOC_Os06g37364	OS06G0570100	LOC_Os06g37364.1	C	22073885..22082490	538	6.93	60,865.40	60,826.09	AFGAGRRACAG	475 - 484	o4-26i339-361o	S	chloroplast	AT5G25900	LOC_Os06g37330
CYP701A6v2	LOC_Os06g37364		LOC_Os06g37364.2	C	22073883..22082490	505	6.72	57,169.89	57,133.00	AFGAGRRACAG	442 - 451	o	S	chloroplast	-	-
CYP701A6v3	LOC_Os06g37364		LOC_Os06g37364.3	C	22073883..22081296	408	6.10	47,303.43	47,272.66	AFGAGRRACAG	345 - 354	o	-	chloroplast	-	-
CYP701A8	LOC_Os06g37300	OS06G0569500	LOC_Os06g37300.1	C	22020840..22028146	510	5.77	56,964.61	56,928.02	AFGAGRRTCAG	447 - 456	i5-27o	S	chloroplast	-	LOC_Os06g37224
CYP701A9	LOC_Os06g37224	OS06G0568600	LOC_Os06g37224.1	C	21982654..21992533	503	6.27	56,159.56	56,123.86	AFGAGRRTCAG	441 - 450	i5-27o	S	chloroplast	-	LOC_Os06g37300
CYP703A3	LOC_Os08g03682	OS08G0131100	LOC_Os08g03682.1	W	1752707..1755653	525	8.42	59,631.46	59,592.76	PFSAGKRKCPG	448 - 457	o	S	ER	AT1G01280	-
CYP704A3	LOC_Os04g48460	OS04G0573900	LOC_Os04g48460.1	W	28892561..28894834	506	8.37	58,168.99	58,131.63	AFQAGPRICLG	446-455	i5-24o	S	ER	-	LOC_Os10g38090<br>LOC_Os10g38110<br>LOC_Os10g38120
CYP704A4	LOC_Os06g03930	OS06G0129900	LOC_Os06g03930.1	C	1581991..1585822	525	7.19	60,017.10	59,978.60	SFNAGPRICLG	461 - 470	i12-34o	S	ER	AT2G44890<br>AT2G45510	-
CYP704A5v1	LOC_Os10g38110	OS10G0525000	LOC_Os10g38110.1	C	20411944..20416491	515	8.36	58,129.30	58,092.31	AFQAGPRICLG	455-464	o15-34i305-327o	-	ER	-	LOC_Os04g48460<br>LOC_Os10g38090<br>LOC_Os10g38120
CYP704A5v2	LOC_Os10g38110		LOC_Os10g38110.2	C	20411944..20414245	415	6.11	46,908.43	46,878.43	AFQAGPRICLG	355-364	o	-	ER	-	-
CYP704A6	LOC_Os10g38120	OS10G0525200	LOC_Os10g38120.1	C	20426804..20430567	532	8.04	60,264.52	60,226.39	AFQAGPRICIG	472-481	i24-46o	-	ER	-	LOC_Os04g48460<br>LOC_Os10g38090<br>LOC_Os10g38110
CYP704A7	LOC_Os10g38090	OS10G0524700	LOC_Os10g38090.1	C	20394588..20399339	511	8.02	57,303.50	57,266.94	AFQAGPRICLG	451-460	o15-34i301-323o	S	ER	-	LOC_Os04g48460<br>LOC_Os10g38110<br>LOC_Os10g38120
CYP704B2	LOC_Os03g07250	OS03G0168600	LOC_Os03g07250.1	C	3701751..3704082	544	8.14	61,182.52	61,143.24	AFQAGPRICLG	484-493	i13-35o	S	ER	AT1G69500	-
CYP706C1	LOC_Os08g43440	OS08G0547900	LOC_Os08g43440.1	C	27479061..27481168	517	6.26	56,307.92	56,272.10	PFGSGRRICAG	447 - 456	i7-26o	S	ER	AT4G12300<br>AT4G12310<br>AT4G12320	-
CYP706C2	LOC_Os01g50490	OS01G0700500	LOC_Os01g50490.1	W	28991234..28993285	506	9.14	56,018.13	55,982.16	PFGSGRRMCAG	436 - 445	i5-22o	M	ER	AT4G12330<br>AT5G44620	-
CYP707A37	LOC_Os09g28390	OS09G0457100	LOC_Os09g28390.1	C	17252028..17255514	500	9.11	56,166.65	56,130.86	PFGNGVHACPG	419 - 428	i5-27o	S	ER	AT3G19270	LOC_Os08g36860
CYP707A5v1	LOC_Os02g47470	OS02G0703600	LOC_Os02g47470.1	W	28995717..28998581	471	9.33	52,920.28	52,886.18	PFGNGTHSCPG	408 - 417	o	S	ER	AT4G19230<br>AT5G45340	-
CYP707A5v2	LOC_Os02g47470		LOC_Os02g47470.2	W	28995717..28998581	441	11.13	47,365.81	47,336.56	PFGNGTHSCPG	338-387	o	-	ER	-	-
CYP707A5v3	LOC_Os02g47470		LOC_Os02g47470.3	W	28996441..28998581	351	10.38	37,846.83	37,823.28	PFGNGTHSCPG	268-287	o	-	ER	-	-
CYP707A6	LOC_Os08g36860	OS08G0472800	LOC_Os08g36860.1	C	23288613..23294164	510	9.58	56,555.55	56,519.45	PFGSGVHACPG	434 - 443	i2-24o	S	ER	AT3G19270	LOC_Os09g28390
CYP709C10	LOC_Os06g09210	OS06G0191700	LOC_Os06g09210.1	W	4630848..4632543	537	8.79	58,452.90	58,414.94	AFSLGQRSCIG	476 - 485	i13-35o	S	ER	-	LOC_Os06g09220
CYP709C11	LOC_Os06g09220	OS06G0191800	LOC_Os06g09220.1	C	4633663..4635469	467	8.35	51,465.46	51,432.10	AFSLGQRSCIG	406 - 415	o	M	ER	-	LOC_Os06g09210
CYP709C12P	LOC_Os07g23710	OS07G0419000	LOC_Os07g23710.1	C	13382831..13383995	290	8.99	32,929.73	32,907.98	SFSSGPRSCIG	229 - 238	o	-	ER	-	-
CYP709C5	LOC_Os07g44140	OS07G0635500	LOC_Os07g44140.1	W	26382581..26385011	517	9.13	58,038.51	58,001.12	SFSSGPRACIG	456 - 465	i7-29o	S	ER	-	LOC_Os07g23570<br>LOC_Os07g44110
CYP709C6	LOC_Os07g44130	OS07G0635300	LOC_Os07g44130.1	W	26375648..26377640	411	9.28	47,049.08	47,018.36	SFSIGLRSCIG	346 - 355	o	-	ER	-	-
CYP709C8	LOC_Os07g44110	OS07G0635200	LOC_Os07g44110.1	W	26367084..26369068	520	9.61	58,779.75	58,741.59	SFSNGPRSCIG	455 - 464	i7-29o	S	ER	-	LOC_Os07g23570<br>LOC_Os07g44140
CYP709C9	LOC_Os07g23570	OS07G0418500	LOC_Os07g23570.1	W	13323931..13326401	519	8.98	58,547.30	58,509.07	SFSSGPRSCIG	458 - 467	i5-27o	S	ER	-	LOC_Os07g44110<br>LOC_Os07g44140
CYP709D1	LOC_Os01g24780	OS01G0349800	LOC_Os01g24780.1	W	13941318..13944691	523	8.91	58,192.52	58,155.75	SFSQGPRGCIG	462 - 471	i5-27o	S	ER	-	LOC_Os01g24780
CYP709E1	LOC_Os03g25480	OS03G0370900	LOC_Os03g25480.1	W	14553102..14556229	518	9.21	58,749.68	58,711.68	GFSIGPRSCIG	457 - 466	i2-24o	M	ER	AT2G46950<br>AT2G46960<br>AT4G27710	LOC_Os03g25480
CYP709E2Pv1	LOC_Os03g25490	OS03G0371000	LOC_Os03g25490.1	W	14559322..14561845	358	7.67	40,215.79	40,189.94	AFSIGPRSCIG	297 - 306	o	-	ER	-	-
CYP709E2Pv2	LOC_Os03g25490		LOC_Os03g25490.2	W	14559322..14561845	248	6.66	27,828.44	27,810.53	AFSIGPRSCIG	187 - 196	o	-	ER	-	-
CYP710A5	LOC_Os01g11270	OS01G0210900	LOC_Os01g11270.1	C	6031025..6032833	511	8.41	56,935.37	56,899.09	AFGAGPHQCVG	451 - 460	o15-37i224-243o	-	ER	-	LOC_Os01g11280<br>LOC_Os01g11300
CYP710A6	LOC_Os01g11280	OS01G0211000	LOC_Os01g11280.1	C	6037423..6039070	522	8.54	58,459.31	58,422.01	AFGAGPHQCVG	455 - 464	o	-	ER	-	LOC_Os01g11270<br>LOC_Os01g11300
CYP710A7	LOC_Os01g11300	OS01G0211200	LOC_Os01g11300.1	C	6057440..6059109	504	8.38	56,633.09	56,596.91	AFGAGPHQCVG	444 - 453	o	S	ER	-	LOC_Os01g11270<br>LOC_Os01g11280
CYP710A8	LOC_Os01g11340	OS01G0211600	LOC_Os01g11340.1	C	6076688..6078711	508	7.23	56,914.21	56,878.01	AFGAGPHQCVG	441 - 450	o10-27i214-233o	S	ER	AT2G28850<br>AT2G28860<br>AT2G34490<br>AT2G34500	-
CYP711A2	LOC_Os01g50530	OS01G0700900	LOC_Os01g50530.1	W	29024351..29025826	411	8.56	46,784.64	46,755.15	PFGIGPRACIG	348 - 357	o	-	mitochondrion	-	LOC_Os01g50580
CYP711A3	LOC_Os01g50580	OS01G0701400	LOC_Os01g50580.1	C	29039999..29041364	385	5.66	43,195.18	43,167.88	PFGIGPRACIG	321 - 330	o	-	ER	-	LOC_Os01g50530
CYP711A4	LOC_Os01g50590	OS01G0701500	LOC_Os01g50590.1	W	29045049..29047994	516	7.75	57,952.52	57,915.19	PFGIGPRVCIG	474 - 483	i13-31o	S	ER	AT2G26170	LOC_Os06g36920
CYP711A5v1	LOC_Os02g12890	OS02G0221900	LOC_Os02g12890.1	C	6802606..6805335	548	8.55	60,552.95	60,514.38	PFGIGPRACVG	484 - 493	i27-49o	-	ER	-	-
CYP711A5v2	LOC_Os02g12890		LOC_Os02g12890.2	C	6802606..6804635	423	7.17	46,720.70	46,690.84	PFGIGPRACVG	359 - 368	o	M	ER	-	-
CYP711A6	LOC_Os06g36920	OS06G0565100	LOC_Os06g36920.1	C	21755618..21758564	549	9.63	59,991.98	59,954.47	PFGLGPRACPG	482 - 491	i20-42o	M	ER	AT2G26170	LOC_Os01g50590
CYP714B1	LOC_Os07g48330	OS07G0681300	LOC_Os07g48330.1	W	28882154..28886452	534	8.20	58,035.76	57,998.57	PFGAGARTCLG	465 - 474	i7-29o	S	ER	-	-
CYP714B2	LOC_Os03g21400	OS03G0332100	LOC_Os03g21400.1	C	12235110..12237748	437	8.83	49,228.17	49,196.72	PFGAGARTCLG	328 - 337	o	-	ER	AT5G24900<br>AT5G24910	LOC_Os12g02640
CYP714C1	LOC_Os12g02630	OS12G0118900	LOC_Os12g02630.1	W	910825..912396	242	6.50	26,806.13	26,788.76	PFGHGPRTCIG	182 - 191	o	-	ER	-	LOC_Os07g48330
CYP714C16P	LOC_Os11g02710	OS11G0119700	LOC_Os11g02710.1	W	876096..879246	571	9.46	62,616.65	62,576.83	PFGVGVRTCAG	512 - 521	o	-	ER	-	-
CYP714C2	LOC_Os12g02640	OS12G0119000	LOC_Os12g02640.1	W	912504..916531	522	9.03	58,814.56	58,776.30	PFGVGVRTCAG	463 - 472	i9-31o	S	ER	AT5G24900<br>AT5G24910	LOC_Os03g21400<br>LOC_Os12g02640
CYP714D1	LOC_Os05g40384	OS05G0482400	LOC_Os05g40384.1	W	23728502..23738405	577	9.00	63,188.55	63,148.25	PFGLGARTCLG	497 - 506	i5-27o	S	ER	-	-
CYP715B1	LOC_Os02g57810	OS02G0824100	LOC_Os02g57810.1	W	35409423..35411018	531	9.23	58,231.40	58,194.28	PFGFGGRICVG	471 - 480	i7-29o	S	ER	-	-
CYP71AA2	LOC_Os01g72760	OS01G0957800	LOC_Os01g72760.1	W	42211882..42213692	528	8.83	58,827.22	58,789.85	PGGSGRRMRPG	443-452 (C->R)	i9-28o	S	ER	AT5G52400	LOC_Os01g72740
CYP71AA3	LOC_Os01g72740	OS01G0957600	LOC_Os01g72740.1	W	42206304..42208513	523	9.18	58,139.65	58,102.62	PGGAGRRMCPG	457-466	o4-23i35-57o	S	ER	-	LOC_Os01g72760
CYP71AB1	LOC_Os10g09160	OS10G0172100	LOC_Os10g09160.1	W	4946936..4948405	446	6.30	51,199.13	51,166.02	PFGAGRRMCPG	377 - 386	o	-	ER	-	-
CYP71AB2	LOC_Os06g30500	OS06G0500700	LOC_Os06g30500.1	C	17630572..17632346	505	6.18	56,859.85	56,823.38	PFGAGRRICPG	437 - 446	i5-27o	S	ER	-	LOC_Os06g30179
CYP71AB3	LOC_Os06g30179	OS06G0497200	LOC_Os06g30179.1	W	17411745..17428547	524	6.17	59,244.04	59,205.44	PFGAGRRICPG	456 - 465	o	-	ER	-	LOC_Os06g30500
CYP71AC2	LOC_Os06g45960	OS06G0671300	LOC_Os06g45960.1	C	27844654..27848256	546	9.01	60,556.09	60,517.62	PFGGGRRICPG	473 - 482	i13-35o	-	ER	-	-
CYP71AC3P	LOC_Os03g39540		LOC_Os03g39540.1	C	21959933..21960490	185	6.11	21,372.65	21,358.86	SFGAGRRMCLG	115 - 124	o	-	ER	-	-
CYP71AD1	LOC_Os05g35010	OS05G0424300	LOC_Os05g35010.1	C	20792434..20794367	542	6.41	59,381.91	59,344.78	PFGAGRRVCPG	475 - 484	i28-50o	-	ER	-	-
CYP71AF1	LOC_Os06g43520	OS06G0642600	LOC_Os06g43520.1	C	26159542..26164597	752	6.37	82,746.99	82,695.08	PFGAGRRMCPG	679 - 688	o	M	ER	-	-
CYP71AK2	LOC_Os09g36080	OS09G0530300	LOC_Os09g36080.1	W	20774283..20776230	507	9.14	54,819.32	54,785.07	PFGAGRRICPG	439 - 448	i2-20o	C	ER	-	-
CYP71C12	LOC_Os08g01450	OS08G0105400	LOC_Os08g01450.1	C	288778..290301	507	7.24	57,052.47	57,015.52	PFGTGRRICPG	442 - 451	i7-26o	S	ER	-	LOC_Os06g22020
CYP71C13P	LOC_Os08g01470	OS08G0105600	LOC_Os08g01470.1	C	293377..294882	340	9.02	38,474.16	38,449.03	PFRTGRRICPG	277-286	i7-29o	S	ER	-	-
CYP71C15	LOC_Os08g01510	OS08G0106000	LOC_Os08g01510.1	W	308328..310185	484	7.75	54,905.70	54,870.50	PFGSGRRMCPG	420 - 429	o	-	ER	-	LOC_Os08g01510
CYP71C16	LOC_Os08g01520	OS08G0106300	LOC_Os08g01520.1	W	318965..321012	524	8.90	59,218.02	59,179.94	PFGSGRRMCPG	458 - 467	i12-34o	S	ER	-	LOC_Os08g01520
CYP71C17	LOC_Os08g01490	OS08G0105800	LOC_Os08g01490.1	C	301642..304056	549	5.93	61,981.36	61,941.45	PFGAGRRICPG	480 - 489	i21-43o	-	ER	-	-
CYP71C19P	LOC_Os01g36294	OS01G0543600	LOC_Os01g36294.1	W	20124194..20126856	362	5.04	40,612.79	40,586.59	PFGAGRRICPG	284 - 293	o	-	ER	-	-
CYP71C20	LOC_Os06g22020	OS06G0325900	LOC_Os06g22020.1	W	12751745..12753286	513	8.74	57,967.75	57,930.23	PFGSGRRICPG	446 - 455	i5-27o	S	ER	-	LOC_Os08g01450
CYP71C32	LOC_Os11g27730	OS11G0465200	LOC_Os11g27730.1	C	15956522..15960376	513	8.90	58,019.29	57,981.61	PFGSGRRMCLG	450 - 459	i9-31o	S	ER	-	-
CYP71C33	LOC_Os11g28060	OS11G0469900	LOC_Os11g28060.1	C	16129383..16130581	219	4.81	25,204.83	25,188.65	PFGYGRRIVPLG	209-218 (C->V)	o	-	ER	-	-
CYP71E4	LOC_Os03g63310	OS03G0850200	LOC_Os03g63310.1	W	35777909..35781336	527	9.46	58,289.68	58,252.61	PFGSGRRICPG	460 - 469	o15-34i317-339o	S	ER	AT2G02580<br>AT3G26290<br>AT3G26300<br>AT3G26310<br>AT3G26320<br>AT3G26330<br>AT5G57260	-
CYP71E5	LOC_Os12g32850	OS12G0512800	LOC_Os12g32850.1	C	19848807..19851543	522	9.06	58,290.55	58,253.66	PFGTGRRICPG	453 - 462	o	S	ER	AT1G13080<br>AT1G13090<br>AT1G13100<br>AT1G13110<br>AT3G26150<br>AT3G26160<br>AT3G26170<br>AT3G26180<br>AT3G26190<br>AT3G26200<br>AT3G26210<br>AT3G26220<br>AT3G26230<br>AT3G26270<br>AT3G26280<br>AT3G26830<br>AT3G44250<br>AT3G53280<br>AT3G53290<br>AT3G53300<br>AT3G53305<br>AT5G35715	-
CYP71E6P	LOC_Os03g37080	OS03G0568400	LOC_Os03g37080.1	W	20551642..20558012	449	6.70	50,353.66	50,321.61	PFGSGRRICPG	381 - 390	o	-	ER	-	-
CYP71K1	LOC_Os01g27890	OS01G0377000	LOC_Os01g27890.1	C	15581786..15583686	538	8.58	59,885.72	59,847.13	PFGAGRRMCPG	466 - 475	i25-47o	-	ER	-	-
CYP71K10	LOC_Os06g43420	OS06G0641600	LOC_Os06g43420.1	C	26097154..26098851	504	8.55	56,862.12	56,825.68	PFGAGRRMCPG	434 - 443	i5-22o	S	ER	-	LOC_Os02g09200<br>LOC_Os02g09220<br>LOC_Os02g09240<br>LOC_Os02g09250<br>LOC_Os06g43350<br>LOC_Os06g43370<br>LOC_Os06g43384<br>LOC_Os06g43430<br>LOC_Os09g10340
CYP71K11	LOC_Os11g41680	OS11G0635300	LOC_Os11g41680.1	W	25021621..25023695	538	9.41	61,391.44	61,352.41	PFGVGRRICPG	467 - 476	i31-53o	-	ER	-	LOC_Os06g43480<br>LOC_Os06g43490<br>LOC_Os11g41710
CYP71K12	LOC_Os11g41710	OS11G0635500	LOC_Os11g41710.1	W	25040876..25042999	512	9.24	58,484.02	58,446.99	PFGVGRRICPG	441 - 450	i12-31o	S	ER	-	LOC_Os06g43480<br>LOC_Os06g43490<br>LOC_Os11g41680
CYP71K3	LOC_Os02g09390	OS02G0186800	LOC_Os02g09390.1	W	4824246..4825855	509	9.12	56,289.63	56,253.40	PFGAGRRICPG	439 - 448	i7-24o	M	ER	-	-
CYP71K4	LOC_Os02g09400	OS02G0186900	LOC_Os02g09400.1	W	4826405..4828225	518	7.83	57,386.37	57,349.88	PFGAGRRICPG	448 - 457	i5-23o	S	ER	-	-
CYP71K5	LOC_Os02g09410	OS02G0187000	LOC_Os02g09410.1	W	4828671..4830333	523	7.80	57,750.87	57,713.95	PFGAGRRMCPG	450 - 459	i5-24o	S	ER	-	-
CYP71K6	LOC_Os06g43490	OS06G0642300	LOC_Os06g43490.1	W	26145806..26148452	504	9.68	57,129.76	57,093.00	PFGAGRRMCPG	438 - 447	i7-29o	S	ER	-	LOC_Os06g43480<br>LOC_Os11g41680<br>LOC_Os11g41710
CYP71K7P	LOC_Os06g43480	OS06G0642200	LOC_Os06g43480.1	W	26137029..26139343	519	9.80	59,555.42	59,517.12	PFGAGRRMCPG	481 - 490	i7-24o	M	ER	-	LOC_Os06g43490<br>LOC_Os11g41680<br>LOC_Os11g41710
CYP71K8	LOC_Os06g43440		LOC_Os06g43440.1	C	26108626..26109554	219	5.17	24,498.19	24,482.47	PFGAGRRICPG	150 - 159	o	-	ER	-	-
CYP71K9	LOC_Os06g43430	OS06G0641800	LOC_Os06g43430.1	C	26105107..26107276	520	6.78	58,125.21	58,088.11	PFGAGRRMCPG	448 - 457	i7-29o	S	ER	-	LOC_Os02g09200<br>LOC_Os02g09220<br>LOC_Os02g09240<br>LOC_Os02g09250<br>LOC_Os06g43350<br>LOC_Os06g43370<br>LOC_Os06g43384<br>LOC_Os06g43420<br>LOC_Os09g10340
CYP71P1	LOC_Os12g16720	OS12G0268000	LOC_Os12g16720.1	C	9579747..9582014	523	7.69	57,764.67	57,728.19	PFGGGRRGCPG	446 - 455	o	C	ER	-	-
CYP71Q1	LOC_Os07g19210	OS07G0293000	LOC_Os07g19210.1	C	11380988..11383790	408	5.75	46,550.71	46,520.75	PFGSGRRICPG	334 - 343	o	-	ER	-	-
CYP71Q2	LOC_Os07g19130		LOC_Os07g19130.1	C	11326338..11327860	220	6.12	25,305.47	25,288.76	PFGSGRRICPG	163 - 172	o	-	ER	-	-
CYP71R1	LOC_Os05g43910	OS05G0515200	LOC_Os05g43910.1	W	25543222..25546410	522	6.45	56,605.82	56,570.31	PFGAGRRGCPG	457 - 466	i7-29o	S	ER	-	LOC_Os06g15680
CYP71R2P	LOC_Os06g15680	OS06G0267400	LOC_Os06g15680.1	C	8876051..8877757	544	10.04	59,676.89	59,639.62	PFGAGRRGCPG	471 - 480	i7-29o	S	ER	-	LOC_Os05g43910
CYP71S1	LOC_Os04g40470	OS04G0480700	LOC_Os04g40470.1	C	24049070..24051142	511	9.41	55,976.30	55,941.47	PFGIGRRRCPG	443 - 452	o	S	ER	-	LOC_Os04g40460
CYP71S2	LOC_Os04g40460	OS04G0480650	LOC_Os04g40460.1	C	24043880..24047054	588	8.96	64,514.87	64,474.54	PFGSGRRLCPG	450 - 459	o	S	ER	-	LOC_Os04g40470
CYP71T1	LOC_Os01g12740	OS01G0227400	LOC_Os01g12740.1	W	7048273..7050877	558	5.53	60,540.44	60,502.44	PFGAGRRGCPG	484 - 493	i7-29o	S	ER	-	LOC_Os01g12750
CYP71T2	LOC_Os01g12750	OS01G0227500	LOC_Os01g12750.1	W	7053100..7056375	561	6.56	60,390.50	60,352.44	PFGAGRRGCPG	487 - 496	i7-29o	S	ER	-	LOC_Os01g12740
CYP71T3	LOC_Os01g12760	OS01G0227700	LOC_Os01g12760.1	W	7061344..7065249	539	6.27	59,817.19	59,779.39	PFGAGRRGCPG	468 - 477	i13-35o	S	ER	AT1G11610<br>AT2G30750<br>AT2G30770<br>AT3G48270<br>AT3G48280<br>AT3G48290<br>AT3G48300<br>AT3G48310<br>AT3G48320<br>AT4G13290<br>AT4G13310<br>AT4G20240<br>AT5G24950<br>AT5G24960<br>AT5G42590	LOC_Os01g12770
CYP71T4	LOC_Os01g12770	OS01G0227800	LOC_Os01g12770.1	W	7071481..7073789	540	8.74	59,730.97	59,693.37	PFGAGRRGCPG	466 - 475	i5-27o	S	ER	AT1G11610<br>AT2G30750<br>AT2G30770<br>AT3G48270<br>AT3G48280<br>AT3G48290<br>AT3G48300<br>AT3G48310<br>AT3G48320<br>AT4G13290<br>AT4G13310<br>AT4G20240<br>AT5G24950<br>AT5G24960<br>AT5G42590	LOC_Os01g12760
CYP71T8	LOC_Os09g36070	OS09G0530275	LOC_Os09g36070.1	W	20768633..20770819	482	6.21	52,561.62	52,528.41	PFGAGRRLCPG	416 - 425	i5-22o	S	ER	-	-
CYP71U3	LOC_Os02g17760	OS02G0278400	LOC_Os02g17760.1	W	10261986..10268672	539	8.15	59,127.07	59,089.67	PFGAGCRQCPG	465 - 474	i17-39o	-	ER	-	-
CYP71V2	LOC_Os09g10340	OS09G0275400	LOC_Os09g10340.1	C	5643294..5645523	509	9.01	56,530.80	56,494.58	PFGSGRRMCPG	440 - 449	o	M	ER	-	LOC_Os02g09200<br>LOC_Os02g09220<br>LOC_Os02g09240<br>LOC_Os02g09250<br>LOC_Os06g43350<br>LOC_Os06g43370<br>LOC_Os06g43384<br>LOC_Os06g43420<br>LOC_Os06g43430
CYP71V3	LOC_Os12g44290	OS12G0640200	LOC_Os12g44290.1	C	27451287..27453035	513	7.66	57,391.50	57,354.83	QFGSGRRMCPG	444 - 453	o	-	ER	-	-
CYP71V4	LOC_Os02g12550	OS02G0217400	LOC_Os02g12550.1	W	6559488..6562188	508	9.05	56,474.08	56,437.72	PFGAGRRMCPG	441 - 450	i7-29o	S	ER	-	LOC_Os02g12540
CYP71V5	LOC_Os02g12540	OS02G0217300	LOC_Os02g12540.1	W	6556026..6558023	511	8.67	56,665.90	56,629.90	PFGAGRRICPG	444 - 453	o	S	ER	-	LOC_Os02g12550
CYP71W1	LOC_Os03g39650	OS03G0593600	LOC_Os03g39650.1	W	22048470..22052542	542	6.69	60,736.27	60,697.50	PFGAGRRMCPG	440 - 449	i5-27o	S	ER	-	-
CYP71W3	LOC_Os03g39690	OS03G0594100	LOC_Os03g39690.1	W	22083215..22086166	512	8.01	57,885.07	57,848.26	PFGAGRRICPG	443 - 452	i7-24o	S	ER	-	LOC_Os03g39760
CYP71W4	LOC_Os03g39760	OS03G0594900	LOC_Os03g39760.1	W	22123330..22125643	515	7.95	57,663.69	57,626.79	PFGSGRRVCPG	446 - 455	i5-23o	S	ER	-	LOC_Os03g39690
CYP71X10	LOC_Os02g09220	OS02G0184900	LOC_Os02g09220.1	W	4740044..4743193	514	6.45	57,024.26	56,987.76	PFGAGRRMCPG	443 - 452	i7-24o	S	ER	-	LOC_Os02g09200<br>LOC_Os02g09240<br>LOC_Os02g09250<br>LOC_Os06g43350<br>LOC_Os06g43370<br>LOC_Os06g43384<br>LOC_Os06g43420<br>LOC_Os06g43430<br>LOC_Os09g10340
CYP71X11	LOC_Os02g09200	OS02G0184700	LOC_Os02g09200.1	C	4724221..4726015	519	7.78	57,564.59	57,528.28	PFGSGRRMCPG	447 - 456	i7-29o	-	ER	-	LOC_Os02g09220<br>LOC_Os02g09240<br>LOC_Os02g09250<br>LOC_Os06g43350<br>LOC_Os06g43370<br>LOC_Os06g43384<br>LOC_Os06g43420<br>LOC_Os06g43430<br>LOC_Os09g10340
CYP71X12	LOC_Os02g09190	OS02G0184600	LOC_Os02g09190.1	C	4720935..4722712	487	9.02	53,499.80	53,465.90	PFGSRRKICPG	410-419	i7-26o	S	ER	-	LOC_Os02g09310<br>LOC_Os02g38930<br>LOC_Os02g38940
CYP71X13P	LOC_Os02g38930	OS02G0601400	LOC_Os02g38930.1	C	23531392..23533277	462	8.79	51,012.19	50,979.50	PFGAGRRSCPG	394 - 403	i9-28o	S	ER	-	LOC_Os02g09290<br>LOC_Os02g09310<br>LOC_Os02g38940
CYP71X14	LOC_Os02g38940	OS02G0601500	LOC_Os02g38940.1	C	23535117..23536974	526	7.72	58,687.90	58,650.24	PFGAGRRICPG	453 - 462	i7-26o	S	ER	-	LOC_Os02g09290<br>LOC_Os02g09310<br>LOC_Os02g38930
CYP71X1P	LOC_Os02g09330	OS02G0186300	LOC_Os02g09330.1	W	4800374..4802382	286	5.83	31,847.21	31,826.31	PFGAGRRMCSG	198-207	i5-27o	S	ER	-	-
CYP71X2	LOC_Os02g09320	OS02G0186200	LOC_Os02g09320.1	W	4796082..4797937	539	7.18	59,819.00	59,781.15	PFGAGRRICPG	455 - 464	i5-22o	S	ER	-	-
CYP71X3	LOC_Os02g09310	OS02G0186100	LOC_Os02g09310.1	W	4791457..4793241	478	6.55	53,645.53	53,611.03	PYGAGRRICPG	409-418	i7-26o	S	ER	-	LOC_Os02g09290<br>LOC_Os02g38930<br>LOC_Os02g38940
CYP71X4	LOC_Os02g09290	OS02G0185900	LOC_Os02g09290.1	W	4785203..4787184	514	6.27	57,632.83	57,596.02	PFGAGRRICPG	445 - 454	i7-26o	S	ER	-	-
CYP71X7	LOC_Os02g09250	OS02G0185300	LOC_Os02g09250.1	W	4765151..4767215	521	7.11	57,652.90	57,616.26	PFGAGRRMCPG	454 - 463	o	S	ER	-	LOC_Os02g09200<br>LOC_Os02g09220<br>LOC_Os02g09240<br>LOC_Os06g43350<br>LOC_Os06g43370<br>LOC_Os06g43384<br>LOC_Os06g43420<br>LOC_Os06g43430<br>LOC_Os09g10340
CYP71X8	LOC_Os02g09240	OS02G0185200	LOC_Os02g09240.1	W	4754173..4756309	514	6.79	56,931.32	56,895.12	PFGAGRRMCPG	443 - 452	i7-24o	S	ER	-	LOC_Os02g09200<br>LOC_Os02g09220<br>LOC_Os02g09250<br>LOC_Os06g43350<br>LOC_Os06g43370<br>LOC_Os06g43384<br>LOC_Os06g43420<br>LOC_Os06g43430<br>LOC_Os09g10340
CYP71Y1P	LOC_Os06g43410	OS06G0641500	LOC_Os06g43410.1	W	26092516..26094621	562	8.86	61,971.45	61,932.53	PFGAGRRMCPG	492 - 501	i7-24o	M	ER	-	-
CYP71Y3	LOC_Os06g43384	OS06G0641200	LOC_Os06g43384.1	W	26082406..26087091	520	7.21	56,771.49	56,735.71	PFGAGRRMCPG	448 - 457	i5-22o	M	ER	-	LOC_Os02g09200<br>LOC_Os02g09220<br>LOC_Os02g09240<br>LOC_Os02g09250<br>LOC_Os06g43350<br>LOC_Os06g43370<br>LOC_Os06g43420<br>LOC_Os06g43430<br>LOC_Os09g10340
CYP71Y4	LOC_Os06g43370	OS06G0640800	LOC_Os06g43370.1	W	26072378..26074146	512	8.62	55,459.21	55,424.21	PFGGGRRMCPG	442 - 451	i5-22o	S	ER	-	LOC_Os02g09200<br>LOC_Os02g09220<br>LOC_Os02g09240<br>LOC_Os02g09250<br>LOC_Os06g43350<br>LOC_Os06g43384<br>LOC_Os06g43420<br>LOC_Os06g43430<br>LOC_Os09g10340
CYP71Y5	LOC_Os06g43350		LOC_Os06g43350.1	W	26060866..26065357	518	8.97	56,577.45	56,541.69	PFGAGRRMCPG	448 - 457	o	M	ER	-	LOC_Os02g09200<br>LOC_Os02g09220<br>LOC_Os02g09240<br>LOC_Os02g09250<br>LOC_Os06g43370<br>LOC_Os06g43384<br>LOC_Os06g43420<br>LOC_Os06g43430<br>LOC_Os09g10340
CYP71Y6	LOC_Os06g43320	OS06G0640100	LOC_Os06g43320.1	W	26034857..26038356	526	6.91	56,987.15	56,950.78	PFGAGRRMCPG	456 - 465	i13-30o	-	ER	-	LOC_Os06g43304
CYP71Y7	LOC_Os06g43304	OS06G0639800	LOC_Os06g43304.1	W	26022267..26029283	528	7.20	57,224.28	57,187.84	PFGAGRRMCPG	457 - 466	o10-32i306-328o	S	ER	-	LOC_Os06g43320
CYP71Z1	LOC_Os04g27020		LOC_Os04g27020.1	W	15986497..15989538	511	8.81	56,641.81	56,605.30	PFGSGRRVCPG	441 - 450	i5-27o	S	ER	-	LOC_Os02g32770<br>LOC_Os02g36150<br>LOC_Os02g36190<br>LOC_Os07g11739<br>LOC_Os07g11870<br>LOC_Os07g11890<br>LOC_Os07g11970<br>LOC_Os10g30380<br>LOC_Os10g30390<br>LOC_Os10g30410
CYP71Z2	LOC_Os07g11739	OS07G0217600	LOC_Os07g11739.1	W	6494465..6502011	517	7.19	57,548.52	57,511.56	PFGSGRRICPG	447 - 456	i5-26o	S	ER	-	LOC_Os02g32770<br>LOC_Os02g36150<br>LOC_Os02g36190<br>LOC_Os04g27020<br>LOC_Os07g11870<br>LOC_Os07g11890<br>LOC_Os07g11970<br>LOC_Os10g30380<br>LOC_Os10g30390<br>LOC_Os10g30410
CYP71Z21	LOC_Os07g11870	OS07G0218700	LOC_Os07g11870.1	W	6574224..6577460	519	8.25	57,343.03	57,306.33	PFGSGHRICPG	449 - 458	o	M	ER	-	LOC_Os02g32770<br>LOC_Os02g36150<br>LOC_Os02g36190<br>LOC_Os04g27020<br>LOC_Os07g11739<br>LOC_Os07g11890<br>LOC_Os07g11970<br>LOC_Os10g30380<br>LOC_Os10g30390<br>LOC_Os10g30410
CYP71Z22	LOC_Os07g11970	OS07G0220200	LOC_Os07g11970.1	C	6632567..6635403	515	8.57	56,981.88	56,945.07	PFGSGHRICPG	445 - 454	o	M	ER	-	LOC_Os02g32770<br>LOC_Os02g36150<br>LOC_Os02g36190<br>LOC_Os04g27020<br>LOC_Os07g11739<br>LOC_Os07g11870<br>LOC_Os07g11890<br>LOC_Os10g30380<br>LOC_Os10g30390<br>LOC_Os10g30410
CYP71Z3	LOC_Os10g30380	OS10G0439700	LOC_Os10g30380.1	C	15789729..15792467	522	7.54	58,257.81	58,220.05	PFGSGRRMCAG	448 - 457	o5-27i108-130o	S	ER	-	LOC_Os02g32770<br>LOC_Os02g36150<br>LOC_Os02g36190<br>LOC_Os04g27020<br>LOC_Os07g11739<br>LOC_Os07g11870<br>LOC_Os07g11890<br>LOC_Os07g11970<br>LOC_Os10g30390<br>LOC_Os10g30410
CYP71Z4	LOC_Os10g30390	OS10G0439800	LOC_Os10g30390.1	C	15796178..15799887	524	8.26	58,312.69	58,275.24	PFGSGRRMCAA	449 - 458	i7-29o	S	ER	-	LOC_Os02g32770<br>LOC_Os02g36150<br>LOC_Os02g36190<br>LOC_Os04g27020<br>LOC_Os07g11739<br>LOC_Os07g11870<br>LOC_Os07g11890<br>LOC_Os07g11970<br>LOC_Os10g30380<br>LOC_Os10g30410
CYP71Z5	LOC_Os02g32770	OS02G0529800	LOC_Os02g32770.1	C	19459069..19463361	513	8.42	56,957.29	56,920.68	PFGSGRRICPG	442 - 451	i5-27o	S	ER	-	LOC_Os02g36150<br>LOC_Os02g36190<br>LOC_Os04g27020<br>LOC_Os07g11739<br>LOC_Os07g11870<br>LOC_Os07g11890<br>LOC_Os07g11970<br>LOC_Os10g30380<br>LOC_Os10g30390<br>LOC_Os10g30410
CYP71Z6	LOC_Os02g36150	OS02G0570500	LOC_Os02g36150.1	W	21788133..21791223	515	8.32	57,156.52	57,119.86	PFGSGRRICPG	445 - 454	o	S	ER	-	LOC_Os02g32770<br>LOC_Os02g36190<br>LOC_Os04g27020<br>LOC_Os07g11739<br>LOC_Os07g11870<br>LOC_Os07g11890<br>LOC_Os07g11970<br>LOC_Os10g30380<br>LOC_Os10g30390<br>LOC_Os10g30410
CYP71Z7	LOC_Os02g36190	OS02G0570700	LOC_Os02g36190.1	C	21810334..21813460	518	8.29	57,415.93	57,379.03	PFGSGRRICPG	444 - 453	i7-26o	S	ER	-	LOC_Os02g32770<br>LOC_Os02g36150<br>LOC_Os04g27020<br>LOC_Os07g11739<br>LOC_Os07g11870<br>LOC_Os07g11890<br>LOC_Os07g11970<br>LOC_Os10g30380<br>LOC_Os10g30390<br>LOC_Os10g30410
CYP71Z8	LOC_Os10g30410	OS10G0439924	LOC_Os10g30410.1	C	15806654..15811018	519	9.05	57,926.20	57,889.21	PFGSGRRICPG	449 - 458	i5-27o	S	ER	-	LOC_Os02g32770<br>LOC_Os02g36150<br>LOC_Os02g36190<br>LOC_Os04g27020<br>LOC_Os07g11739<br>LOC_Os07g11870<br>LOC_Os07g11890<br>LOC_Os07g11970<br>LOC_Os10g30380<br>LOC_Os10g30390
CYP721B1	LOC_Os05g33600	OS05G0405600	LOC_Os05g33600.1	C	19756222..19758913	510	9.32	57,298.74	57,262.44	PFGIGPTICVG	451 - 460	i2-24o	S	ER	AT1G75130	LOC_Os05g33590
CYP721B2	LOC_Os05g33590	OS05G0405500	LOC_Os05g33590.1	C	19742291..19744851	581	9.39	65,914.71	65,872.27	PFGIGPTICVG	450 - 459	i7-29o	S	ER	AT1G75130	LOC_Os05g33600
CYP722B1	LOC_Os05g01120	OS05G0101600	LOC_Os05g01120.1	C	101365..104149	518	9.46	58,607.88	58,570.19	VFGAGGRTCLG	456 - 465	i7-29o	S	ER	AT1G19630	LOC_Os05g01120
CYP723A2	LOC_Os04g03870	OS04G0128001	LOC_Os04g03870.1	C	1767007..1768449	480	8.74	52,823.81	52,790.48	PFGAGRRYCPG	412 - 421	o4-21i172-191o	M	ER	-	LOC_Os04g03890
CYP723A3	LOC_Os04g03890	OS04G0128400	LOC_Os04g03890.1	C	1779894..1781348	484	10.66	53,346.88	53,313.24	PFGAGRRSCPG	418 - 427	i5-22o	S	ER	-	LOC_Os04g03870
CYP724B1	LOC_Os04g39430	OS04G0469800	LOC_Os04g39430.1	C	23467128..23471594	480	7.99	53,704.40	53,670.16	PFGGGPRLCPG	419 - 428	i7-29o	S	ER	-	-
CYP727A1	LOC_Os07g45000	OS07G0644600	LOC_Os07g45000.1	W	26844721..26850330	566	8.06	61,785.74	61,745.98	PFGSGSRACVG	502 - 511	o10-32i45-64o	-	mitochondrion	-	-
CYP728A1	LOC_Os09g21260	OS09G0380300	LOC_Os09g21260.1	W	12831415..12832878	487	9.93	53,034.26	53,000.29	PFGGGARACPG	422 - 431	i2-24o	C	ER	-	LOC_Os07g33620.1
CYP728B1	LOC_Os07g33620	OS07G0520300	LOC_Os07g33620.1	W	20084700..20086612	498	9.47	54,939.83	54,905.05	AFGGGPRICPG	428 - 437	i2-24o	C	ER	-	LOC_Os09g21260.1
CYP728B3	LOC_Os07g33440	OS07G0518100	LOC_Os07g33440.1	W	19985712..19987668	492	9.97	54,189.21	54,154.47	AFGGGPRICPG	431 - 440	i5-27o	C	ER	-	LOC_Os07g33620
CYP728C1v1	LOC_Os07g33610	OS07G0520100	LOC_Os07g33610.1	W	20077861..20081621	436	9.57	49,463.42	49,432.06	PFGGGPRMCPG	414 - 423	o4-23i71-93o	M	ER	-	LOC_Os07g33480<br>LOC_Os07g33540<br>LOC_Os07g33550<br>LOC_Os07g33560<br>LOC_Os07g33580
CYP728C1v2	LOC_Os07g33610		LOC_Os07g33610.2	W	20077848..20081621	363	9.15	41,044.43	41,018.46	PFGGGPRMCPG	341 - 350	o	-	ER	-	-
CYP728C3	LOC_Os07g33580	OS07G0519600	LOC_Os07g33580.1	W	20063394..20065355	475	9.31	53,794.43	53,760.12	PFGGGPRMCPG	414 - 423	i7-24o	S	ER	-	LOC_Os07g33480<br>LOC_Os07g33540<br>LOC_Os07g33550<br>LOC_Os07g33560<br>LOC_Os07g33610
CYP728C4	LOC_Os07g33560	OS07G0519300	LOC_Os07g33560.1	W	20050247..20054045	407	8.41	45,949.15	45,919.76	PFGGGPSMCPG	341-350	o	-	ER	-	LOC_Os07g33480<br>LOC_Os07g33540<br>LOC_Os07g33550<br>LOC_Os07g33580<br>LOC_Os07g33610
CYP728C5	LOC_Os07g33550	OS07G0519100	LOC_Os07g33550.1	W	20045846..20049071	479	9.13	54,203.98	54,169.32	PFGGGPRMCPG	414 - 423	i7-24o	S	ER	-	LOC_Os07g33480<br>LOC_Os07g33540<br>LOC_Os07g33560<br>LOC_Os07g33580<br>LOC_Os07g33610
CYP728C7	LOC_Os07g33540		LOC_Os07g33540.1	W	20040134..20043772	383	9.47	44,061.28	44,033.05	PFGGGPRMCPG	317 - 326	i7-24o	S	ER	-	LOC_Os07g33480<br>LOC_Os07g33550<br>LOC_Os07g33560<br>LOC_Os07g33580<br>LOC_Os07g33610
CYP728C9v1	LOC_Os07g33480	OS07G0518500	LOC_Os07g33480.1	W	20012734..20015644	483	8.88	54,405.02	54,370.15	PFGGGPRMCPG	417 - 426	o5-22i280-302o	S	ER	-	LOC_Os07g33540<br>LOC_Os07g33550<br>LOC_Os07g33560<br>LOC_Os07g33580<br>LOC_Os07g33610
CYP728C9v2	LOC_Os07g33480		LOC_Os07g33480.4	W	20012734..20015644	283	6.01	32,082.76	32,062.23	PFGGGPRMCPG	217 - 226	o	M	ER	-	-
CYP729A1v1	LOC_Os10g23180	OS10G0378100	LOC_Os10g23180.1	C	12090254..12098641	501	8.83	55,414.67	55,379.36	VFGGGERICAG	443 - 452	o10-29i49-71o	S	ER	-	-
CYP729A1v2	LOC_Os10g23180		LOC_Os10g23180.2	C	12090255..12098641	410	9.06	44,882.63	44,853.92	VFGGGERICAG	352 - 361	o10-29i49-71o	S	ER	-	-
CYP729A2	LOC_Os10g23130	OS10G0378000	LOC_Os10g23130.1	C	12078273..12080274	131	8.44	15,293.72	15,283.67	VFGGGERICAG	60-69	i105-127o	-	ER	-	-
CYP72A17v1	LOC_Os01g43700	OS01G0627400	LOC_Os01g43700.1	W	25040701..25044050	554	9.46	62,002.09	61,962.53	PFSWGPRICIG	495 - 504	i7-29o39-61i	-	ER	-	-
CYP72A17v2	LOC_Os01g43700		LOC_Os01g43700.2	W	25040214..25044050	547	9.48	61,129.09	61,090.12	PFSWGPRICIG	488 - 497	o5-24i31-53o	-	ER	-	-
CYP72A18	LOC_Os01g43710	OS01G0627500	LOC_Os01g43710.1	C	25045176..25049414	523	9.31	59,873.58	59,835.10	PFGWGPRICIG	464 - 473	i7-29o	S	ER	AT3G14610<br>AT3G14620<br>AT3G14630<br>AT3G14640<br>AT3G14650<br>AT3G14660<br>AT3G14680<br>AT3G14690	-
CYP72A20	LOC_Os01g43740	OS01G0627800	LOC_Os01g43740.1	W	25060764..25063609	534	7.64	60,187.51	60,148.96	PFGWGPRICIG	475 - 484	o	-	ER	-	-
CYP72A21	LOC_Os01g43750	OS01G0627900	LOC_Os01g43750.1	W	25066171..25068299	528	8.92	60,177.37	60,138.24	PFGWGPRICIG	469 - 478	i7-29o	M	ER	-	-
CYP72A22	LOC_Os01g43760	OS01G0627933	LOC_Os01g43760.1	W	25070716..25072705	471	9.41	53,228.06	53,194.10	PFSWGPRICIG	412 - 421	o	M	ER	-	-
CYP72A23	LOC_Os01g43774	OS01G0628000	LOC_Os01g43774.1	W	25073849..25078549	650	9.06	72,766.20	72,719.44	PFGWGPRVCIG	591 - 600	o	-	ER	-	-
CYP72A24	LOC_Os01g43844	OS01G0628700	LOC_Os01g43844.1	W	25114081..25118368	531	9.01	59,470.06	59,431.80	PFGWGPRICIG	471 - 480	o	S	ER	-	-
CYP72A25	LOC_Os01g43851	OS01G0628900	LOC_Os01g43851.1	W	25119734..25121927	528	9.13	59,333.59	59,295.05	PFSWGPRICVG	469 - 478	i13-31o	S	ER	-	-
CYP72A31P	LOC_Os01g41800	OS01G0602200	LOC_Os01g41800.1	C	23663059..23664444	294	6.40	33,583.10	33,561.12	PFGWGPRICIG	235 - 244	o	-	ER	-	-
CYP72A32	LOC_Os01g41810	OS01G0602400	LOC_Os01g41810.1	C	23673292..23675869	535	9.11	61,387.85	61,348.13	PFGWGPRICIG	476 - 485	o10-32i341-363o	S	ER	-	LOC_Os01g41820
CYP72A33	LOC_Os01g41820	OS01G0602500	LOC_Os01g41820.1	C	23682382..23685393	552	8.63	63,024.43	62,983.65	PFGWGPRICIG	483 - 492	i5-27o	S	ER	-	-
CYP72A34	LOC_Os05g30890	OS05G0372300	LOC_Os05g30890.1	W	17934628..17941865	519	9.15	59,296.03	59,258.13	PFGWGPRICIG	460 - 469	i7-29o	S	ER	-	-
CYP72A35	LOC_Os01g52790	OS01G0728300	LOC_Os01g52790.1	C	30372204..30376917	519	9.14	59,124.35	59,086.73	SFAWGPRICLG	460-469	i11-33o	S	ER	-	-
CYP733A1	LOC_Os03g61980		LOC_Os03g61980.1	W	35131484..35134664	537	9.80	60,221.41	60,183.56	GFGSGPRMCPG	403 - 412	i2-21o	M	ER	-	LOC_Os03g61980
CYP734A2	LOC_Os02g11020	OS02G0204700	LOC_Os02g11020.1	C	5862711..5865569	557	9.29	61,964.47	61,924.81	PFGLGSRMCVG	488 - 497	i13-35o	-	ER	-	LOC_Os02g11020
CYP734A4	LOC_Os06g39880	OS06G0600400	LOC_Os06g39880.1	W	23704714..23707248	538	9.31	59,680.18	59,642.05	PFGLGSRMCIG	473 - 482	i5-27o	S	ER	-	LOC_Os06g39880
CYP734A5	LOC_Os07g45290	OS07G0647200	LOC_Os07g45290.1	C	27019245..27023518	537	8.75	59,909.82	59,871.35	PFGGGARVCIG	473 - 482	i7-29o	S	ER	-	LOC_Os07g45290
CYP734A6	LOC_Os01g29150	OS01G0388000	LOC_Os01g29150.1	C	16325487..16328374	589	9.71	66,070.97	66,028.64	PFGLGARMCIG	514 - 523	o5-27i48-65o	M	ER	AT2G26710	LOC_Os01g29150
CYP735A3	LOC_Os08g33300	OS08G0429800	LOC_Os08g33300.1	C	20761534..20763853	534	9.67	58,582.94	58,545.64	PFAAGPRNCVG	470-479	i5-27o	S	ER	AT1G67110<br>AT5G38450	LOC_Os09g23820
CYP735A4	LOC_Os09g23820	OS09G0403300	LOC_Os09g23820.1	C	14194357..14196590	535	9.83	59,412.04	59,374.20	PFAAGPRNCVG	474-483	o	S	ER	AT1G67110<br>AT5G38450	LOC_Os08g33300
CYP73A35P	LOC_Os01g60450	OS01G0820000	LOC_Os01g60450.1	C	34961976..34963642	481	9.49	54,654.27	54,619.96	PSGAGRRSCPG	416-425	i5-24o	S	ER	AT2G30490	LOC_Os05g25640
CYP73A38	LOC_Os05g25640	OS05G0320700	LOC_Os05g25640.1	C	14899987..14904546	500	9.02	56,883.21	56,846.98	PFGVGRRSCPG	435 - 444	i5-24o	S	ER	AT2G30490	LOC_Os01g60450
CYP73A39	LOC_Os02g26810	OS02G0467600	LOC_Os02g26810.1	W	15734823..15736934	533	8.77	59,480.82	59,443.03	PFGVGRRSCPG	468 - 477	o	S	ER	-	LOC_Os02g26770
CYP73A40	LOC_Os02g26770	OS02G0467000	LOC_Os02g26770.1	C	15716825..15718857	533	8.77	59,498.82	59,461.09	PFGVGRRSCPG	468 - 477	o	S	ER	-	LOC_Os02g26810
CYP74A4	LOC_Os03g55800	OS03G0767000	LOC_Os03g55800.1	C	31764987..31766985	512	9.22	56,504.19	56,468.59	PTLHDKQCAG	459-468	o	C	chloroplast	AT5G42650	-
CYP74A5	LOC_Os03g12500	OS03G0225900	LOC_Os03g12500.1	W	6608577..6610598	478	8.18	52,271.00	52,238.26	PSVDNKQCPG	443-452	o	M	chloroplast	-	-
CYP74E1	LOC_Os02g12680	OS02G0218700	LOC_Os02g12680.1	W	6628383..6630103	500	6.52	54,438.62	54,403.97	PSEGNKQCPG	446-455	o	-	chloroplast	-	LOC_Os02g12690
CYP74E2	LOC_Os02g12690	OS02G0218800	LOC_Os02g12690.1	W	6637973..6639881	510	6.91	55,329.69	55,294.59	PSEGNKQCPG	448-457	o	-	chloroplast	-	LOC_Os02g12680
CYP74F1	LOC_Os02g02000	OS02G0110200	LOC_Os02g02000.1	W	554513..556320	487	5.60	53,710.67	53,676.76	PSPGNKQCAA	439-448	o	-	chloroplast	AT4G15440	-
CYP75A11	LOC_Os03g25150	OS03G0367101	LOC_Os03g25150.1	W	14381298..14387811	519	7.60	56,342.38	56,306.24	PFGAGRRICAG	450 - 459	o	S	ER	-	-
CYP75B11	LOC_Os10g16974	OS10G0317900	LOC_Os10g16974.1	W	8494248..8504329	535	6.69	58,518.64	58,481.54	PFGAGRRICAG	462 - 471	i7-29o	S	ER	-	-
CYP75B3	LOC_Os10g17260	OS10G0320100	LOC_Os10g17260.1	W	8679310..8681284	526	8.16	57,517.28	57,480.85	PFGAGRRICAG	454 - 463	o4-26i75-97o	S	ER	AT5G07990	-
CYP76H11	LOC_Os08g12990	OS08G0226300	LOC_Os08g12990.1	C	7705969..7716625	1452	9.20	163,422.93	163,319.46	PFGAGRRICPG	444 - 453	o	M	ER	-	-
CYP76H4	LOC_Os03g14400	OS03G0248200	LOC_Os03g14400.1	C	7831876..7833791	501	8.99	55,428.09	55,393.27	PFGSGRRICPG	437 - 446	i3-25o	S	ER	AT1G33720<br>AT1G33730<br>AT2G45550<br>AT2G45560<br>AT2G45570<br>AT2G45580<br>AT3G61040	LOC_Os03g14420<br>LOC_Os10g08319<br>LOC_Os10g08474<br>LOC_Os10g08540
CYP76H5	LOC_Os03g14420	OS03G0248300	LOC_Os03g14420.1	C	7840807..7843174	500	8.58	56,354.14	56,318.53	PFGSGRRICPG	436 - 445	i3-25o	S	ER	AT1G33720<br>AT1G33730<br>AT2G45550<br>AT2G45560<br>AT2G45570<br>AT2G45580<br>AT3G61040	LOC_Os03g14400<br>LOC_Os10g08319<br>LOC_Os10g08474<br>LOC_Os10g08540
CYP76H6	LOC_Os10g08540	OS10G0167250	LOC_Os10g08540.1	W	4629293..4636151	835	9.35	92,582.92	92,525.13	PFGAGRRICPG	771 - 780	i5-27o	S	ER	AT1G33720<br>AT1G33730<br>AT2G45550<br>AT2G45560<br>AT2G45570<br>AT2G45580<br>AT3G61040	LOC_Os03g14400<br>LOC_Os03g14420<br>LOC_Os10g08319<br>LOC_Os10g08474
CYP76H8	LOC_Os10g08474	OS10G0166600	LOC_Os10g08474.1	W	4599938..4602910	494	8.33	55,131.96	55,097.13	PFGAGRRICPG	430 - 439	i5-27o	S	ER	AT1G33720<br>AT1G33730<br>AT2G45550<br>AT2G45560<br>AT2G45570<br>AT2G45580<br>AT3G61040	LOC_Os03g14400<br>LOC_Os03g14420<br>LOC_Os10g08319<br>LOC_Os10g08540
CYP76H9	LOC_Os10g08319	OS10G0165300	LOC_Os10g08319.1	W	4497746..4503667	501	8.23	56,127.25	56,091.63	PFGAGRRICPG	437 - 446	i5-27o	S	ER	AT1G33720<br>AT1G33730<br>AT2G45550<br>AT2G45560<br>AT2G45570<br>AT2G45580<br>AT3G61040	LOC_Os03g14400<br>LOC_Os03g14420<br>LOC_Os10g08474<br>LOC_Os10g08540
CYP76K1	LOC_Os09g27510	OS09G0447500	LOC_Os09g27510.1	C	16718630..16720923	505	7.19	54,589.94	54,555.66	PFGSGRRACPG	441 - 450	i7-24o	S	ER	-	-
CYP76L1	LOC_Os09g27500	OS09G0447300	LOC_Os09g27500.1	C	16710805..16713794	510	6.99	55,491.92	55,456.94	PFGAGRRACPG	442 - 451	o	S	ER	-	-
CYP76M1	LOC_Os08g36310	OS08G0465700	LOC_Os08g36310.1	W	22876101..22877810	500	8.77	55,985.95	55,950.09	PFGSGRRVCAG	435 - 444	o6-23i	M	ER	-	-
CYP76M10	LOC_Os08g39660	OS08G0507100	LOC_Os08g39660.1	C	25109655..25111322	505	7.19	56,187.96	56,152.34	PFGIGRRICPG	437 - 446	i5-24o	M	ER	-	LOC_Os08g39694<br>LOC_Os08g39730
CYP76M11P	LOC_Os08g39640	OS08G0507000	LOC_Os08g39640.1	W	25102165..25105616	311	6.03	34,720.22	34,697.79	PFWAGRRLCPG	239-248	o	C	ER	-	-
CYP76M13	LOC_Os12g09790	OS12G0199800	LOC_Os12g09790.1	C	5184081..5186323	488	8.81	54,001.98	53,967.61	PLGSGRRLCPA	421-430	o	M	ER	-	-
CYP76M14	LOC_Os01g38110	OS01G0561600	LOC_Os01g38110.1	C	21342129..21344066	524	6.90	58,277.17	58,240.47	PFGSGRRLCPG	458 - 467	i7-25o	S	ER	-	-
CYP76M17	LOC_Os02g36110	OS02G0569900	LOC_Os02g36110.1	W	21740304..21742030	499	8.71	55,418.58	55,383.16	PFGSGRRLCPG	434 - 443	i7-24o	M	ER	-	LOC_Os02g36070<br>LOC_Os02g36280<br>LOC_Os06g39780
CYP76M2	LOC_Os08g39730	OS08G0508000	LOC_Os08g39730.1	W	25155158..25156888	506	6.40	55,745.19	55,709.97	PFGAGRRLCPG	433 - 442	i5-24o	M	ER	-	LOC_Os08g39660<br>LOC_Os08g39694
CYP76M4Pv1	LOC_Os08g39694	OS08G0507702	LOC_Os08g39694.1	W	25121291..25134371	463	9.08	50,785.93	50,753.48	PFGTGRRLCPG	395 - 404	i5-24o	M	ER	-	LOC_Os08g39660<br>LOC_Os08g39730
CYP76M4Pv2	LOC_Os08g39694		LOC_Os08g39694.2	W	25131585..25134371	463	9.08	50,785.93	50,753.48	PFGTGRRLCPG	395 - 404	i5-24o	M	ER	-	-
CYP76M4Pv3	LOC_Os08g39694		LOC_Os08g39694.3	W	25121291..25134371	427	12.12	47,214.55	47,186.39	PFGTGRRLCPG	359-368	o	M	ER	-	-
CYP76M5	LOC_Os02g36030	OS02G0569000	LOC_Os02g36030.1	W	21662608..21664323	501	7.24	55,243.77	55,209.04	PFGSRRKICPG	436 - 445	i7-29o	S	ER	-	LOC_Os02g36070<br>LOC_Os02g36110<br>LOC_Os02g36280<br>LOC_Os06g39780
CYP76M6	LOC_Os02g36280	OS02G0571900	LOC_Os02g36280.1	C	21904354..21906350	542	8.66	59,858.03	59,820.05	PFGSGRRLCPG	475 - 484	i38-56o	-	ER	-	LOC_Os02g36030<br>LOC_Os02g36070<br>LOC_Os02g36110<br>LOC_Os06g39780
CYP76M7	LOC_Os06g39780	OS06G0599200	LOC_Os06g39780.1	W	23632462..23634203	500	8.69	55,521.72	55,486.21	PFGSGRRLCPG	435 - 444	i7-24o	M	ER	-	LOC_Os02g36030<br>LOC_Os02g36070<br>LOC_Os02g36110<br>LOC_Os02g36280
CYP76M8	LOC_Os02g36070	OS02G0569400	LOC_Os02g36070.1	W	21690378..21692186	500	7.84	55,933.24	55,897.32	PFGSGRRLCPG	435 - 444	i7-24o	M	ER	-	LOC_Os02g36030<br>LOC_Os02g36110<br>LOC_Os02g36280<br>LOC_Os06g39780
CYP76M9	LOC_Os06g30640	OS06G0501900	LOC_Os06g30640.1	C	17737912..17739429	505	8.25	55,731.61	55,696.08	PFGAGRRMCPG	439 - 448	i5-23o	M	ER	AC186603.6_FG003	-
CYP76N1P	LOC_Os02g29720	OS02G0500700	LOC_Os02g29720.1	C	17676359..17678224	424	9.65	48,073.76	48,043.37	PFGGGRRICLG	356 - 365	i5-27o	M	ER	-	-
CYP76P1	LOC_Os10g05490	OS10G0144700	LOC_Os10g05490.1	C	2709104..2711914	515	8.11	56,803.83	56,767.39	PFSAGRRFCLG	449 - 458	i2-24o	S	ER	-	LOC_Os12g09500
CYP76P2	LOC_Os12g09500	OS12G0196700	LOC_Os12g09500.1	C	4984225..4988325	511	9.34	56,678.64	56,642.89	PFSAGRRFCLG	445 - 454	i2-24o	S	ER	-	LOC_Os10g05490
CYP76P3	LOC_Os10g21050	OS10G0351200	LOC_Os10g21050.1	C	10662425..10663461	253	5.57	28,260.73	28,242.55	PFSAGRRVCLG	189 - 198	o	-	ER	-	-
CYP76Q1	LOC_Os03g14560	OS03G0249600	LOC_Os03g14560.1	C	7896938..7901673	499	9.06	55,829.17	55,793.54	PFGVGRRICLG	435 - 444	i2-24o	S	ER	-	LOC_Os06g19070
CYP76Q2	LOC_Os06g19070	OS06G0294600	LOC_Os06g19070.1	C	10843920..10846020	518	6.79	56,973.29	56,937.18	PFGLGRRICPG	454 - 463	i5-27o	S	ER	-	LOC_Os03g14560
CYP76V1	LOC_Os10g09090	OS10G0171300	LOC_Os10g09090.1	C	4914321..4917390	424	6.30	46,562.94	46,533.43	PFGAGRRICLG	403 - 412	o	-	ER	-	-
CYP77A18	LOC_Os04g33370	OS04G0407900	LOC_Os04g33370.1	W	20211340..20213320	528	9.69	58,644.29	58,606.82	PFSAGRRICPG	462 - 471	i12-34o	S	ER	AT3G10560<br>AT3G10570<br>AT5G04630<br>AT5G04660	-
CYP77B2	LOC_Os06g46680	OS06G0680700	LOC_Os06g46680.1	W	28354739..28358400	599	8.22	65,594.86	65,552.45	PFGAGRRICPA	519 - 528	o	-	ER	AT1G11600	-
CYP78A11	LOC_Os10g26340	OS10G0403000	LOC_Os10g26340.1	C	13658790..13660543	555	6.58	59,083.65	59,046.20	PFGAGRRVCPG	488 - 497	i35-57o	-	ER	AT5G09970	-
CYP78A12	LOC_Os03g30420	OS03G0417700	LOC_Os03g30420.1	C	17340415..17342284	515	9.00	56,047.90	56,012.25	PFGAGRRACPG	454 - 463	i13-35o	-	ER	-	-
CYP78A13	LOC_Os07g41240	OS07G0603700	LOC_Os07g41240.1	W	24713778..24715813	525	8.72	55,890.96	55,855.30	PFGMGRRGCPG	464 - 473	i13-35o	C	ER	AT1G13710<br>AT1G74110	-
CYP78A14	LOC_Os03g40600	OS03G0603100	LOC_Os03g40600.1	C	22567670..22568685	193	7.75	20,287.42	20,274.37	PFGAGRRACPG	128 - 137	o	-	ER	-	-
CYP78A15	LOC_Os08g43390	OS08G0547300	LOC_Os08g43390.1	C	27420501..27422836	551	9.21	59,793.00	59,755.02	PFGSGRRSCPG	485 - 494	i32-54o	-	ER	AT2G46660<br>AT3G61880	-
CYP78A16	LOC_Os09g35940	OS09G0528700	LOC_Os09g35940.1	W	20691306..20693116	553	9.13	60,741.14	60,702.64	PFGSGRRSCPG	487 - 496	o31-53i73-95o	-	ER	-	-
CYP78A17	LOC_Os03g04190	OS03G0134500	LOC_Os03g04190.1	C	1920043..1921896	515	8.15	55,803.27	55,767.63	PFGSGRRSCPG	452 - 461	i5-27o	S	ER	-	-
CYP78D1	LOC_Os11g29720	OS11G0489250	LOC_Os11g29720.1	C	17234285..17238178	538	9.80	59,635.49	59,596.88	PFGSGRRVCPG	475 - 484	i5-22o27-49i	S	ER	-	-
CYP79A10	LOC_Os04g08824	OS04G0171600	LOC_Os04g08824.1	W	4869932..4872151	491	9.26	55,726.83	55,690.91	SFSAGRRGCPA	455 - 464	i13-35o	S	ER	-	-
CYP79A7	LOC_Os03g37290	OS03G0570100	LOC_Os03g37290.1	C	20666009..20670823	547	8.84	60,620.96	60,582.75	SFSTGRRGCPG	476 - 485	i12-29o	C	ER	AT2G22330<br>AT4G39950<br>AT5G05260<br>AT5G35917	LOC_Os04g08824<br>LOC_Os04g08828
CYP79A9	LOC_Os04g08828	OS04G0171800	LOC_Os04g08828.1	W	4874374..4878345	550	8.45	62,232.20	62,192.12	SFSAGRRGCPA	477 - 486	i21-43o	-	ER	AT2G22330<br>AT4G39950<br>AT5G05260<br>AT5G35917	LOC_Os03g37290<br>LOC_Os04g08824
CYP79A9P	LOC_Os04g09430	OS04G0174100	LOC_Os04g09430.1	C	5040350..5043003	253	7.79	28,175.49	28,157.53	SFSAGRRGCPA	180 - 189	o	M	ER	AT2G22330<br>AT4G39950<br>AT5G05260<br>AT5G35917	LOC_Os03g37290<br>LOC_Os04g08824<br>LOC_Os04g08828
CYP81A6	LOC_Os03g55240	OS03G0760200	LOC_Os03g55240.1	W	31440748..31446119	732	7.66	80,401.22	80,350.26	PFGMGRRRCPG	660 - 669	i223-242o	-	ER	AT1G66540<br>AT2G23190<br>AT2G23220<br>AT3G28740<br>AT4G37320<br>AT4G37330<br>AT4G37340<br>AT4G37360<br>AT4G37370<br>AT5G36220	LOC_Os04g18380<br>LOC_Os12g39240<br>LOC_Os12g39300
CYP81A8	LOC_Os03g55260	OS03G0760500	LOC_Os03g55260.1	W	31453545..31455662	518	9.16	57,892.42	57,854.70	PFGMGRRRCPG	446 - 455	i5-22o	S	ER	-	-
CYP81L2	LOC_Os02g30110	OS02G0504000	LOC_Os02g30110.1	W	17880886..17886427	526	7.66	58,057.24	58,019.64	PFGAGRRLCPG	456 - 465	i30-52o	-	ER	-	LOC_Os02g30080<br>LOC_Os02g30100
CYP81L3	LOC_Os02g30100	OS02G0503900	LOC_Os02g30100.1	W	17873662..17876075	524	9.32	58,315.63	58,278.00	PFGMGRRRCPG	453 - 462	i7-24o	C	ER	-	LOC_Os02g30080<br>LOC_Os02g30110
CYP81L4	LOC_Os02g30090	OS02G0503800	LOC_Os02g30090.1	W	17867904..17871190	509	8.98	56,932.94	56,896.14	PFGMGRCKCPA	440-449	o	M	ER	-	-
CYP81L5	LOC_Os02g30080	OS02G0503700	LOC_Os02g30080.1	W	17862194..17866853	525	8.98	58,605.89	58,568.25	PFGMGRRKCPA	456 - 465	i5-22o	M	ER	-	LOC_Os02g30100<br>LOC_Os02g30110
CYP81M1	LOC_Os04g18380	OS04G0255600	LOC_Os04g18380.1	W	10144587..10150653	618	6.55	67,484.32	67,441.29	PFGMGRRRCPG	539 - 548	o	-	ER	AT1G66540<br>AT2G23190<br>AT2G23220<br>AT3G28740<br>AT4G37320<br>AT4G37330<br>AT4G37340<br>AT4G37360<br>AT4G37370<br>AT5G36220	LOC_Os03g55240<br>LOC_Os12g39240<br>LOC_Os12g39300
CYP81N1	LOC_Os12g39240	OS12G0582000	LOC_Os12g39240.1	C	24154936..24156950	544	6.61	58,399.47	58,362.60	PFGLGRRRCPG	472 - 481	i12-34o	S	ER	AT1G66540<br>AT2G23190<br>AT2G23220<br>AT3G28740<br>AT4G37320<br>AT4G37330<br>AT4G37340<br>AT4G37360<br>AT4G37370<br>AT5G36220	LOC_Os03g55240<br>LOC_Os04g18380<br>LOC_Os12g39300
CYP81N1P	LOC_Os12g39300		LOC_Os12g39300.1	C	24154798..24157079	508	6.55	54,733.93	54,699.30	PFGLGRRRCPG	436 - 445	o	C	ER	AT1G66540<br>AT2G23190<br>AT2G23220<br>AT3G28740<br>AT4G37320<br>AT4G37330<br>AT4G37340<br>AT4G37360<br>AT4G37370<br>AT5G36220	LOC_Os03g55240<br>LOC_Os04g18380<br>LOC_Os12g39240
CYP81P1	LOC_Os12g39310	OS12G0582700	LOC_Os12g39310.1	C	24184198..24186545	533	9.75	58,183.89	58,146.36	PFGMGRRKCPG	462 - 471	i13-35o	S	ER	-	-
CYP84A5	LOC_Os10g36848	OS10G0512400	LOC_Os10g36848.1	C	19728254..19735041	530	6.16	57,988.93	57,951.56	PFGSGRRSCPG	460 - 469	o	-	ER	AT4G36220	-
CYP84A6	LOC_Os03g02180	OS03G0112900	LOC_Os03g02180.1	C	726213..728004	519	6.72	56,443.30	56,407.24	PFGSGRRACPA	450 - 459	i13-31o	S	ER	-	-
CYP84A7	LOC_Os06g24180	OS06G0349700	LOC_Os06g24180.1	C	14150816..14153224	529	6.95	58,986.12	58,948.23	PFGSGRRSCPG	459 - 468	o57-74i81-103o	-	ER	-	-
CYP85A1	LOC_Os03g40540	OS03G0602300	LOC_Os03g40540.1	W	22538341..22542520	469	9.33	52,822.40	52,788.33	LFGGGSRMCPG	412 - 421	i2-24o	S	ER	AT3G30180<br>AT5G38970	-
CYP86A10v1	LOC_Os02g44654	OS02G0666500	LOC_Os02g44654.1	C	27063038..27065234	532	8.47	59,139.98	59,102.25	AFNAGPRICLG	452 - 461	i2-21o	S	ER	AT1G01600<br>AT1G63710<br>AT2G45970<br>AT4G00360	LOC_Os04g47250
CYP86A10v2	LOC_Os02g44654		LOC_Os02g44654.2	C	27063038..27065234	532	8.47	59,139.98	59,102.25	AFNAGPRICLG	452 - 461	i2-21o	S	ER	-	-
CYP86A11	LOC_Os04g47250	OS04G0560100	LOC_Os04g47250.1	C	28061310..28063652	545	8.70	60,100.89	60,062.76	AFNAGPRICLG	460 - 469	i5-22o	S	ER	AT1G01600<br>AT1G63710<br>AT2G45970<br>AT4G00360	LOC_Os02g44654
CYP86A9	LOC_Os01g63540	OS01G0854800	LOC_Os01g63540.1	W	36840077..36842130	545	8.98	61,146.31	61,107.57	AFNGGPRLCLG	458 - 467	i13-32o	S	ER	AT5G58860	-
CYP86B3	LOC_Os10g34480	OS10G0486100	LOC_Os10g34480.1	C	18389977..18394398	560	8.37	61,698.19	61,658.71	VFNGGPRLCLG	496 - 505	o	-	ER	AT5G08250<br>AT5G23190	-
CYP86E1v1	LOC_Os02g38290	OS02G0596300	LOC_Os02g38290.1	C	23152692..23154898	546	9.33	59,394.04	59,356.65	VFNAGPRLCIG	449 - 458	o	S	ER	AT1G24540	-
CYP86E1v2	LOC_Os02g38290		LOC_Os02g38290.2	C	23152689..23154879	545	9.25	59,300.91	59,263.58	VFNAGPRLCIG	449 - 458	o	S	ER	-	-
CYP87A4v1	LOC_Os04g48210	OS04G0570600	LOC_Os04g48210.1	C	28715432..28720804	486	9.16	54,943.04	54,907.82	AFGGGLRFCVG	428 - 437	o4-21i283-305o	M	ER	AT1G12740	LOC_Os02g45280<br>LOC_Os04g48170
CYP87A4v2	LOC_Os04g48210		LOC_Os04g48210.2	C	28715432..28720804	253	8.77	28,816.69	28,798.26	AFGGGLRFCVG	195 - 204	o	-	ER	-	-
CYP87A5	LOC_Os02g45280	OS02G0675400	LOC_Os02g45280.1	C	27507229..27511091	506	8.47	56,976.43	56,939.39	AFGGGLRFCVG	448 - 457	i5-27o	S	ER	-	LOC_Os04g48210
CYP87A6	LOC_Os04g48170	OS04G0570000	LOC_Os04g48170.1	C	28672747..28677225	514	8.99	57,406.94	57,369.78	AFGGGLRFCVG	456 - 465	o38-60i311-333o	S	ER	AT1G12740	LOC_Os02g45280
CYP87B1	LOC_Os11g18570	OS11G0289700	LOC_Os11g18570.1	W	10474864..10477749	484	8.81	55,412.19	55,376.52	PFGGGIRMCPA	426 - 435	i7-29o	S	ER	-	LOC_Os04g48200
CYP87B4	LOC_Os04g48200	OS04G0570500	LOC_Os04g48200.1	C	28703939..28708196	482	8.47	55,090.86	55,055.46	PFGGGVRLCLG	424 - 433	i7-29o	S	ER	-	LOC_Os11g18570
CYP87B5	LOC_Os07g29960	OS07G0482900	LOC_Os07g29960.1	C	17640723..17643252	404	6.23	46,669.94	46,640.05	PFGGGIRLCLG	359 - 368	o	-	ER	-	-
CYP87C2v1	LOC_Os03g45619	OS03G0658800	LOC_Os03g45619.1	C	25746001..25760794	495	8.76	55,950.24	55,914.05	AFGGGLRLCVG	437 - 446	i13-35o	S	ER	-	LOC_Os03g45619
CYP87C2v2	LOC_Os03g45619		LOC_Os03g45619.2	C	25746001..25760794	501	8.82	56,645.02	56,608.41	AFGGGLRLCVG	443 - 452	i13-35o	S	ER	-	-
CYP87C5P	LOC_Os12g18820	OS12G0286300	LOC_Os12g18820.1	C	10900850..10906863	317	6.80	36,113.16	36,089.52	AFGGGRRLCVG	259 - 268	o	-	ER	-	-
CYP88A5	LOC_Os06g02019	OS06G0110000	LOC_Os06g02019.1	W	580665..586450	506	9.43	57,008.13	56,971.19	PFGLGARLCPG	444 - 453	i13-35o	S	ER	AT1G05160<br>AT2G32440	LOC_Os06g02019
CYP89B1	LOC_Os10g37160	OS10G0515900	LOC_Os10g37160.1	W	19881378..19883255	516	8.70	57,760.91	57,724.38	PFGVGRRICAG	450 - 459	o4-22i211-228o	S	ER	-	-
CYP89B10	LOC_Os10g36960	OS10G0513400	LOC_Os10g36960.1	W	19804480..19806081	533	9.24	59,508.97	59,471.41	PFGAGRRICAA	308 - 320	i7-25o	S	ER	-	-
CYP89B11	LOC_Os10g05020	OS10G0139700	LOC_Os10g05020.1	C	2453869..2455724	530	8.38	58,225.58	58,188.24	PFGAGRRICPG	464 - 473	i5-27o	S	ER	AT1G64900<br>AT1G64930<br>AT1G64940<br>AT1G64950<br>AT2G12190	LOC_Os06g22340<br>LOC_Os10g05020<br>LOC_Os10g37100
CYP89B12P	LOC_Os06g42610	OS06G0632300	LOC_Os06g42610.1	W	25620940..25622315	253	9.22	27,474.67	27,456.96	PFGVKRRICVG	224-233	o	C	ER	-	-
CYP89B2	LOC_Os10g37120	OS10G0515400	LOC_Os10g37120.1	C	19864267..19865826	519	6.73	58,279.42	58,242.13	PFGVGRRICPG	454 - 463	o4-23i	M	ER	-	-
CYP89B3	LOC_Os10g37110	OS10G0515200	LOC_Os10g37110.1	W	19858612..19860691	523	9.14	58,157.49	58,120.45	PFGVGRRICAG	460 - 469	o4-23i216-233o	M	ER	-	-
CYP89B4	LOC_Os10g37100	OS10G0514901	LOC_Os10g37100.1	C	19856623..19858161	512	8.96	58,038.51	58,001.30	PFGVGRRICAG	449 - 458	i5-24o	M	ER	AT1G64900<br>AT1G64930<br>AT1G64940<br>AT1G64950<br>AT2G12190	LOC_Os06g22340<br>LOC_Os08g05620<br>LOC_Os10g05020
CYP89B5P	LOC_Os10g37070	OS10G0514700	LOC_Os10g37070.1	W	19847510..19848778	422	6.30	47,832.27	47,801.71	PFGAGRRICAG	334 - 343	o	M	ER	-	-
CYP89B6	LOC_Os10g37050	OS10G0514500	LOC_Os10g37050.1	W	19837812..19839400	514	7.72	57,499.68	57,462.78	PFGVGRRICAG	450 - 459	i9-26o	S	ER	-	-
CYP89B7P	LOC_Os10g37034	OS10G0514450	LOC_Os10g37034.1	W	19829259..19832723	241	6.60	27,350.97	27,332.48	PFGVGRRMCAG	164 - 173	i7-29o	S	ER	-	-
CYP89B8P	LOC_Os10g37020	OS10G0514300	LOC_Os10g37020.1	W	19826811..19828235	439	9.64	48,877.85	48,846.55	PFGVGRRMCAG	422 - 431	i5-22o	S	ER	-	-
CYP89B9	LOC_Os10g36980	OS10G0513900	LOC_Os10g36980.1	W	19810180..19812075	520	8.91	58,149.38	58,112.81	PFGVGRRICAG	458 - 467	i7-24o	M	ER	-	-
CYP89C1	LOC_Os06g22340	OS06G0328900	LOC_Os06g22340.1	C	12967302..12969111	514	7.63	56,586.46	56,550.63	PFGAGRRICPG	439 - 448	o	S	ER	AT1G64900<br>AT1G64930<br>AT1G64940<br>AT1G64950<br>AT2G12190	LOC_Os08g05620<br>LOC_Os10g05020<br>LOC_Os10g37100
CYP89C8P	LOC_Os08g05610	OS08G0152300	LOC_Os08g05610.1	C	3000430..3001833	467	8.78	51,458.55	51,425.88	PFGAGRRVCPG	411 - 420	o	S	ER	-	-
CYP89C9	LOC_Os08g05620	OS08G0152400	LOC_Os08g05620.1	W	3007241..3009195	516	6.25	56,387.94	56,352.18	PFGAGRRVCPG	446 - 455	o	S	ER	AT1G64900<br>AT1G64930<br>AT1G64940<br>AT1G64950<br>AT2G12190	LOC_Os06g22340<br>LOC_Os10g05020<br>LOC_Os10g37100
CYP89D1	LOC_Os01g24810	OS01G0350200	LOC_Os01g24810.1	W	13977964..13979538	524	9.06	58,426.93	58,389.79	PFGVGRRICPG	457 - 466	i3-25o	S	ER	-	-
CYP89E1	LOC_Os02g01890	OS02G0108800	LOC_Os02g01890.1	W	488913..490612	504	7.16	56,801.67	56,765.92	PFGVGRRMCPG	437 - 446	i5-22o	M	ER	-	-
CYP89F1	LOC_Os10g36740	OS10G0511200	LOC_Os10g36740.1	W	19671515..19673639	507	9.46	55,988.50	55,953.08	PFSAGRRTCPG	437 - 446	i7-29o	S	ER	-	-
CYP89G1	LOC_Os07g26870	OS07G0451300	LOC_Os07g26870.1	W	15560341..15561945	510	8.37	55,678.30	55,642.97	PFGAGRRACPG	442 - 451	i2-24o39-61i216-238o	S	ER	-	-
CYP90A19	LOC_Os12g04480	OS12G0139300	LOC_Os12g04480.1	C	1905848..1909152	501	9.37	55,719.63	55,684.39	PFGGGPRLCPG	439 - 448	i5-27o	S	ER	AT5G05690	LOC_Os11g04710
CYP90A3	LOC_Os11g04710	OS11G0143200	LOC_Os11g04710.1	C	2006968..2010121	501	9.31	55,710.76	55,675.46	PFGGGPRLCPG	439 - 448	i5-27o	S	ER	AT5G05690	LOC_Os12g04480
CYP90B2	LOC_Os03g12660	OS03G0227700	LOC_Os03g12660.1	W	6737549..6744506	506	9.20	57,328.52	57,291.78	PYGGGTRLCAG	442-451	i12-31o	S	ER	AT3G50660	-
CYP90D2v1	LOC_Os01g10040	OS01G0197100	LOC_Os01g10040.1	W	5236623..5244011	490	9.41	54,824.61	54,789.88	PFGGGQRLCPG	430 - 439	i2-24o	S	ER	AT3G13730	-
CYP90D2V2	LOC_Os01g10040		LOC_Os01g10040.2	W	5238162..5244520	243	5.84	27,888.15	27,870.39	PFGGGQRLCPG	183 - 192	o	M	ER	-	-
CYP90D3	LOC_Os05g11130	OS05G0200400	LOC_Os05g11130.1	W	6267906..6273074	503	9.18	55,440.36	55,405.06	PFGGGQRLCPG	446 - 455	i7-29o	S	ER	-	-
CYP92A11	LOC_Os09g26940	OS09G0441100	LOC_Os09g26940.1	C	16361323..16363289	525	7.27	58,441.70	58,404.78	PFGSGRRLCPG	452 - 461	i5-27o	S	ER	-	-
CYP92A12	LOC_Os08g35510	OS08G0456200	LOC_Os08g35510.1	C	22369377..22370939	520	8.29	58,459.84	58,422.37	PFGSGRRMCPG	448 - 457	o4-23i68-90o	M	ER	-	-
CYP92A13	LOC_Os09g08920	OS09G0263933	LOC_Os09g08920.1	C	4737231..4737840	193	5.60	21,399.91	21,386.17	PFGTGRRMCPG	120 - 129	o	-	ER	-	-
CYP92A14	LOC_Os09g08990	OS09G0264400	LOC_Os09g08990.1	C	4775513..4778505	519	7.73	58,179.55	58,142.36	PFSSGRRMCPG	446 - 455	i7-26o	S	ER	-	-
CYP92A15	LOC_Os02g29960	OS02G0503100	LOC_Os02g29960.1	W	17805488..17809024	537	9.00	60,143.43	60,105.41	PFGSGRRMCPG	465 - 474	o10-32i227-246o	S	ER	-	-
CYP92A7	LOC_Os09g26980	OS09G0441700	LOC_Os09g26980.1	C	16403633..16408074	522	8.80	59,621.94	59,584.16	PFGSGRRMCPG	449 - 458	o4-23i68-90o	S	ER	-	-
CYP92A8	LOC_Os09g26970	OS09G0441600	LOC_Os09g26970.1	W	16392870..16401322	1933	8.07	216,995.00	216,858.91	PFGSGRRMCPG	444 - 453	i3-25o	M	ER	-	-
CYP92A9	LOC_Os09g26960	OS09G0441400	LOC_Os09g26960.1	W	16387701..16390415	514	8.83	58,069.47	58,032.54	PFGSGRRMCPG	441 - 450	i5-27o	S	ER	-	-
CYP92C21	LOC_Os03g44740	OS03G0650200	LOC_Os03g44740.1	C	25219692..25221526	537	8.37	57,388.91	57,352.87	PFGSGRRVCPA	462 - 471	i5-22o	C	ER	-	-
CYP93F1	LOC_Os06g41070	OS06G0613600	LOC_Os06g41070.1	C	24531826..24533798	528	9.00	58,021.01	57,984.30	PFGSGRRICPG	454 - 463	i5-27o	M	ER	AT5G06900	-
CYP93G1v1	LOC_Os04g01140	OS04G0101400	LOC_Os04g01140.1	C	99618..101643	516	7.33	57,110.33	57,073.88	PFGSGRRGCPG	447 - 456	i12-34o	S	ER	-	-
CYP93G1v2	LOC_Os04g01140		LOC_Os04g01140.2	C	99618..101643	354	8.94	38,821.45	38,796.34	PFGSGRRGCPG	285 - 294	i12-34o	S	ER	-	-
CYP93G2	LOC_Os06g01250	OS06G0102100	LOC_Os06g01250.1	W	163205..165539	518	8.44	56,680.66	56,644.52	PFGSGRRACPG	446 - 455	o4-26i115-134o	-	ER	-	-
CYP94B4	LOC_Os11g29290	OS11G0483000	LOC_Os11g29290.1	W	16985590..16987260	556	9.07	59,867.67	59,829.52	VFQGGPRTCLG	476-485	o	S	ER	AT3G48520<br>AT5G63450	LOC_Os12g25660
CYP94B5	LOC_Os12g25660	OS12G0443000	LOC_Os12g25660.1	C	14866006..14867813	529	8.90	57,004.45	56,968.15	VFQGGPRTCLG	460-469	i7-29o	S	ER	AT3G48520<br>AT5G63450	LOC_Os11g29290
CYP94C2	LOC_Os11g05380	OS11G0151400	LOC_Os11g05380.1	C	2390446..2392366	520	8.62	56,579.14	56,542.77	VFQAGQRVCLG	450-459	i19-41o	-	ER	-	LOC_Os12g05440
CYP94C3v1	LOC_Os01g63930	OS01G0858350	LOC_Os01g63930.1	C	37095403..37099183	524	9.07	57,790.86	57,753.91	VFQAGVRVCIG	452-461	o	-	ER	AT2G27690	LOC_Os05g37250
CYP94C3v2	LOC_Os01g63930		LOC_Os01g63930.2	C	37095403..37099183	524	9.07	57,790.86	57,753.91	VFQAGVRVCIG	452-461	o	-	ER	-	-
CYP94C4	LOC_Os05g37250	OS05G0445100	LOC_Os05g37250.1	W	21790820..21793270	519	8.81	57,143.12	57,106.36	VFQGGVRVCVG	452-461	i21-43o	S	ER	AT2G27690	LOC_Os01g63930
CYP94C79	LOC_Os12g05440	OS12G0150200	LOC_Os12g05440.1	C	2468054..2469583	509	8.85	55,215.95	55,180.28	VFQAGLRVCLG	441-450	i7-29o	S	ER	-	LOC_Os11g05380
CYP94D10	LOC_Os01g58990		LOC_Os01g58990.1	W	34094609..34096144	511	9.04	58,254.24	58,216.91	VFNAGPRICIG	449 - 458	i7-29o	S	ER	AT1G34540<br>AT3G56630	LOC_Os01g58950<br>LOC_Os01g58960<br>LOC_Os01g58970<br>LOC_Os01g59020<br>LOC_Os01g59050
CYP94D11	LOC_Os01g58970	OS01G0803900	LOC_Os01g58970.1	W	34089461..34091401	525	6.51	59,527.84	59,489.88	IFHAGPRMCLG	464 - 473	i21-40o	M	ER	AT1G34540<br>AT3G56630	LOC_Os01g58950<br>LOC_Os01g58960<br>LOC_Os01g58990<br>LOC_Os01g59020<br>LOC_Os01g59050
CYP94D12	LOC_Os01g58960		LOC_Os01g58960.1	W	34084757..34086514	508	6.42	57,813.87	57,776.91	VFHAGPRMCLG	447 - 456	o	S	ER	AT1G34540<br>AT3G56630	LOC_Os01g58950<br>LOC_Os01g58970<br>LOC_Os01g58990<br>LOC_Os01g59020<br>LOC_Os01g59050
CYP94D13	LOC_Os01g58950		LOC_Os01g58950.1	W	34074828..34076357	497	6.07	56,325.09	56,289.20	VFHAGPRMCLG	436 - 445	o	S	ER	AT1G34540<br>AT3G56630	LOC_Os01g58960<br>LOC_Os01g58970<br>LOC_Os01g58990<br>LOC_Os01g59020<br>LOC_Os01g59050
CYP94D15	LOC_Os03g12260	OS03G0223100	LOC_Os03g12260.1	C	6443453..6445473	540	9.40	61,033.73	60,995.63	SFHGGPRVCLG	479 - 488	i19-41o	S	ER	-	-
CYP94D4	LOC_Os11g04310		LOC_Os11g04310.1	C	1795375..1796922	515	6.75	57,855.66	57,818.55	VFHAGPRMCLG	448 - 457	o	S	ER	-	LOC_Os11g04290<br>LOC_Os12g04100
CYP94D5	LOC_Os11g04290	OS11G0138300	LOC_Os11g04290.1	W	1775407..1777730	542	6.12	61,083.88	61,044.76	VFHAGPRMCLG	475 - 484	o	M	ER	-	LOC_Os11g04310<br>LOC_Os12g04100
CYP94D6	LOC_Os01g59050	OS01G0804900	LOC_Os01g59050.1	W	34131829..34133381	490	6.49	55,682.62	55,646.82	VFHGGQRMCLG	425 - 434	o5-27i	S	ER	AT1G34540<br>AT3G56630	LOC_Os01g58950<br>LOC_Os01g58960<br>LOC_Os01g58970<br>LOC_Os01g58990<br>LOC_Os01g59020
CYP94D63	LOC_Os12g04100	OS12G0134900	LOC_Os12g04100.1	W	1716158..1718032	518	6.04	58,430.89	58,393.46	VFHAGPRMCLG	451 - 460	i7-29o	S	ER	-	LOC_Os11g04290<br>LOC_Os11g04310
CYP94D64	LOC_Os12g04110	OS12G0135000	LOC_Os12g04110.1	C	1719707..1726259	1595	8.62	177,667.60	177,553.17	VFHAGPRMCLG	448 - 457	o	S	ER	AT1G52620	LOC_Os11g04295
CYP94D7	LOC_Os01g59020	OS01G0804400	LOC_Os01g59020.1	W	34115002..34116761	511	6.39	58,354.62	58,317.51	VFHAGPRMCLG	449 - 458	i7-24o	S	ER	AT1G34540<br>AT3G56630	LOC_Os01g58950<br>LOC_Os01g58960<br>LOC_Os01g58970<br>LOC_Os01g58990<br>LOC_Os01g59050
CYP94D9	LOC_Os01g59000		LOC_Os01g59000.1	W	34099444..34101175	515	7.26	58,291.83	58,254.70	VFHGGPRMCLG	447 - 456	i3-25o	S	ER	-	-
CYP94E1	LOC_Os01g72270		LOC_Os01g72270.1	C	41900002..41901651	549	6.66	60,304.74	60,266.62	VFHAGPRSCLG	476 - 485	i5-27o	S	ER	-	-
CYP94E2	LOC_Os01g72260	OS01G0951500	LOC_Os01g72260.1	C	41896504..41898331	546	9.02	60,508.37	60,470.13	VFHAGPRACLG	472 - 481	i7-25o	S	ER	-	-
CYP94E3	LOC_Os05g31740	OS05G0382500	LOC_Os05g31740.1	W	18487281..18489223	526	8.18	57,619.85	57,583.28	VFHAGPRACLG	461 - 470	i5-22o	S	ER	-	-
CYP96B2	LOC_Os03g04630	OS03G0139600	LOC_Os03g04630.1	W	2170213..2171945	536	8.66	60,682.52	60,643.02	PFITGPRTCLG	445-454	i2-24o	S	ER	-	LOC_Os03g04640
CYP96B3	LOC_Os03g04650	OS03G0140100	LOC_Os03g04650.1	W	2199772..2201650	508	8.55	57,300.44	57,263.37	SFNSGPRTCLG	445 - 454	i7-26o	S	ER	AT1G47620<br>AT1G57750<br>AT1G65340<br>AT2G21910<br>AT2G23180<br>AT4G32170<br>AT4G39480<br>AT4G39490<br>AT4G39500<br>AT4G39510<br>AT5G02900<br>AT5G52320	-
CYP96B4	LOC_Os03g04680	OS03G0140400	LOC_Os03g04680.1	W	2223102..2225205	538	9.19	60,434.32	60,394.94	AFNSGPRTCLG	473 - 482	o13-30i43-65o	S	ER	-	-
CYP96B5	LOC_Os03g04660	OS03G0140200	LOC_Os03g04660.1	W	2211807..2213941	516	6.08	57,609.28	57,571.92	SFNAGPRTCLG	451 - 460	i5-24o	S	ER	-	-
CYP96B6	LOC_Os03g04530	OS03G0138200	LOC_Os03g04530.1	W	2115174..2117105	522	8.56	58,054.42	58,016.68	PFNAGPRLCLG	463 - 472	i13-30o	S	ER	-	-
CYP96B7	LOC_Os08g16430	OS08G0264700	LOC_Os08g16430.1	W	10051525..10053152	398	9.55	43,455.73	43,427.25	AFNSGARMCPG	337 - 346	i5-27o	S	ER	-	-
CYP96B8	LOC_Os08g16260	OS08G0262500	LOC_Os08g16260.1	W	9921522..9923224	520	8.54	58,165.81	58,127.47	SFNSGPRMCPG	459 - 468	o	S	ER	-	LOC_Os08g16320
CYP96B9	LOC_Os03g04640		LOC_Os03g04640.1	W	2178693..2180216	507	8.84	56,856.85	56,820.06	TFSSGPRTCLG	443 - 452	o	S	ER	-	LOC_Os03g04630
CYP96D1	LOC_Os01g08800	OS01G0183500	LOC_Os01g08800.1	W	4417283..4419160	510	8.29	58,534.18	58,496.00	SFNTGPRSCLG	448 - 457	o5-27i302-324o	S	ER	-	LOC_Os01g08800
CYP96D2	LOC_Os05g08850	OS05G0181200	LOC_Os05g08850.1	W	4885395..4888011	516	6.44	56,054.80	56,018.86	AFNAGPRSCLG	454 - 463	o4-26i39-61o	S	ER	-	-
CYP96E1	LOC_Os01g08810	OS01G0183600	LOC_Os01g08810.1	W	4421318..4423107	505	9.20	55,327.14	55,291.66	AFNVGPRTCLG	444 - 453	o	S	ER	-	-
CYP97A4v1	LOC_Os02g57290	OS02G0817900	LOC_Os02g57290.1	C	35091194..35099291	643	5.65	69,861.98	69,817.64	PFGGGPRKCVG	534 - 543	o	C	ER	AT1G31800	-
CYP97A4v2	LOC_Os02g57290		LOC_Os02g57290.2	C	35092344..35099291	643	5.65	69,861.98	69,817.64	PFGGGPRKCVG	534 - 543	o	C	ER	-	-
CYP97A4v3	LOC_Os02g57290		LOC_Os02g57290.3	C	35092344..35099291	643	5.65	69,861.98	69,817.64	PFGGGPRKCVG	534 - 543	o	C	ER	-	-
CYP97A4v4	LOC_Os02g57290		LOC_Os02g57290.4	C	35092344..35099291	643	5.65	69,861.98	69,817.64	PFGGGPRKCVG	534 - 543	o	C	ER	-	-
CYP97B4v1	LOC_Os02g07680	OS02G0173100	LOC_Os02g07680.1	W	3996149..4002697	571	6.63	64,133.77	64,093.33	PFGGGPRKCVG	510 - 519	o	C	ER	AT4G15110	-
CYP97B4v2	LOC_Os02g07680		LOC_Os02g07680.2	W	3995980..4002697	494	5.65	56,178.68	56,143.16	PFGGGPRKCVG	433 - 442	o	-	ER	-	-
CYP97B4v3	LOC_Os02g07680		LOC_Os02g07680.3	W	3996127..4002697	391	5.33	44,632.08	44,604.01	PFGGGPRKCVG	330 - 339	o	-	ER	-	-
CYP97B4v4	LOC_Os02g07680		LOC_Os02g07680.4	W	3995980..4002697	391	5.33	44,632.08	44,604.01	PFGGGPRKCVG	330 - 339	o	-	ER	-	-
CYP97B4v5	LOC_Os02g07680		LOC_Os02g07680.5	W	3995980..4006898	494	5.65	56,178.68	56,143.16	PFGGGPRKCVG	433 - 442	o	-	ER	-	-
CYP97C2v1	LOC_Os10g39930	OS10G0546600	LOC_Os10g39930.1	W	21368384..21373100	561	6.45	62,138.66	62,099.51	PFSGGPRKCVG	488 - 497	o	C	ER	AT3G53130	-
CYP97C2v2	LOC_Os10g39930		LOC_Os10g39930.2	W	21368384..21373363	561	6.45	62,138.66	62,099.51	PFSGGPRKCVG	488 - 497	o	C	ER	-	-
CYP98A4v1	LOC_Os05g41440	OS05G0494000	LOC_Os05g41440.1	W	24271077..24275174	512	8.96	58,358.60	58,321.50	PFGAGRRVCPG	434 - 443	o	S	ER	AT2G40890	-
CYP98A4v2	LOC_Os05g41440		LOC_Os05g41440.2	W	24272786..24275174	362	7.17	41,332.67	41,306.32	PFGAGRRVCPG	284 - 293	o	-	ER	-	-
CYP99A2	LOC_Os04g10160	OS04G0180400	LOC_Os04g10160.1	W	5484768..5486773	532	8.43	59,533.09	59,494.65	PFGTGRRRCPG	461 - 470	i28-50o	C	ER	-	LOC_Os04g09920
CYP99A3	LOC_Os04g09920	OS04G0178400	LOC_Os04g09920.1	C	5332329..5334479	502	6.09	55,891.83	55,855.92	PFGTGRRRCPG	431 - 440	o4-26i106-128o	C	ER	-	LOC_Os04g10160
