# Supplementary material for: Global identification, structural analysis and expression characterization of cytochrome P450 monooxygenase superfamily in rice
Source: BMC Genomics. 2018 Jan 10;19:35. doi: 10.1186/s12864-017-4425-8 (PMC5764023; doi:10.1186/s12864-017-4425-8)
Supplement: Supplementary file 3 — Motif with best possible match and its number in each subfamily. (PDF 56 kb) [file 12864_2017_4425_MOESM3_ESM.pdf]

Table S2. Motif with best possible match and its number in each subfamily.

Motif	Best possible match	Width[1]	74 (5)	51 (10)	710 (4)	733 (1)	722 (1)	88 (1)	729 (2)	728 (9)	707 (3)	85 (1)	90 (5)	724 (1)	87 (8)	727 (1)	714 (6)	715 (1)	735 (2)	72 (13)	721 (2)	734 (4)	709 (10	97 (3)	704 (6)	96 (11)	86 (5)	94 (21)	711 (5)	701 (4)	77 (2)	89 (19)	723 (2)	73 (4)	98 (1)	78 (8)	81 (10)	706 (2)	76 (28)	703 (1)	79 (4)	75 (3)	84 (3)	92 (9)	93 (3)	71 (80)	99 (2)
1	LPFGAGRRICPGKNLAL	17	3	11	4	1	1	1	2	9	3	1	5	1	8	1	6	1	2	14	2	4	10	3	6	11	5	22	5	4	2	19	2	4	1	8	10	2	28	1	4	3	3	9	3	79	2
2	YLKAVIKETLRLHPP	15	7	13	4		1	1	1	9	3	1	5	1	7	1	5	1	2	14	2	4	10	3	6	10	5	21	5	4	2	19	2	4	1	8	10	2	28	1	4	3	3	9	3	79	2
3	WEDPEEFRPERFLDG	15	5	10	4	1	1	1	2	9	3	1	5	1	7	1	6	1	2	13	3	4	10	3	9	11	5	21	5	4	2	19	2	7	1	8	10	2	28	1	4	3	3	9	3	81	2
4	AEEDVEVGGYDVPKGTRVLVN	21		6	4	1	1	1	2	9	3	1	5	1	7	1	6	1	1	13	2	4	10	3	6	10	5	20	5	4	2	19		4	1	8	10	2	28	1	4	3	2	10	3	78	2
5	DLLLDFFFAGTDTTSTTLEWA	21	1	9	4	1	1	1	1	8	3	2	5	1	8	1	6	1	2	12	2	4	9	4	6	10	5	20	6	4	2	18	2	3	1	8	10	2	28	1	4	3	3	8	3	76	3
6	GEYWRQLRKICAVELLSARRV	21					1	1					2				5	1	2	12	2	4	8	2	6	11	5	21			2	14	2	4	1	7	11	2	26	1	3	3	3	8	3	72	2
7	MAELLRNPEVMAKAQEEV	18		7	4	1	2	1	1	9	3	1	5	1	8	1	5	1	2	12	3	4	9	3	6	10	5	21	5	4	2	18	2	4	1	7	10	2	29	1	4	3	3	10	3	78	2
8	RLPPGPWGLPVIGN	14	3	3	4	1		1	1	7	3	1	5	1	6								1		1	10	4	15	3	4	2	15		4	1	5	9	2	24	1	3	3	3	8	3	72	2
9	EVELALASLLYHFDW	15		9	1	1	1	1	2	8	3	1	5	1	10	1	6	1	2	13	3	4	10	3	6	11	3	22	5	4	2	17	3	4	1	8	10	2	26	1	3	3	3	9	3	81	2
10	VVVSSPEAAREVLRTHDAAFA	21			1	1	1			4	3	1	3	1	7	1	2		2	11	2	4	6	3	6	11	5	21	3	4	2	15	2	4	1	7	10	2	24	1	3	3	3	9	3	73	2
11	QDLLQRLTFDVISRAAFGSR	20	2	9					1	8	3		3	1	7	1	4	1	2	12	2	4	10	3	6	11	5	21	5			15	2	3	1	7	6		1	1	3	3	3	9	3	71	2
12	HRALRDLARRHGPVM	15		2		1				7	3	1	1		7	1	3	1		5		1							3	4	2	16		4	1	1	8	2	26	1		3	3	8	3	70	2
13	EELDMTEKFGLTVRRKTPLLA	21																										1		2	2	14	2		1	7	10	2	25	1		3	3	9	3	75	2
14	VWAIGRDP	8		1		1											1	1	2	9		4	6	2	5	11	3	20	1			14			1	8	10	2	28		1	3	2	9	3	79	2
15	DDLLDVLLRLQKEGG	15		9	5	1	1	1	1	8	3		3	1	8	1	4	1		13		2	9		6	1	5	21	5	2	1	17	2	4	1	7	9	2	31	1	3	2	2	8	3	71	2
16	SFRRVREEEVARLVR	15	1	1													1							1								2		2	1	2	4	2	26		1	3	1	8	3	73	
17	LLGGFNVSDLFPSLR	15																								2					2	2		6	1	7	10	2	27	1	3	3	3	7	3	70	2
18	RRVDEFLDRIIRERR	15	3	9		1		1	1	7	3	1	4	1	7		1	1		13		2	10	1	6	8	5	21	1		1	15	2	1	1	7	8	2	23	1	3	2	3	8	3	73	1
19	AIDFKGTDFEF	11																										1				1		2	1	4		1	24			3	3	9	3	73	2
20	RILTYGGRDIAFAPY	15															1														2	7		4	1		9		1	1	1	3	3	8	3	70	2
21	LVTEDDLSKLP	11	1	1	4				1	9	1	1	3		8				1	11	1	6	10	3	3			2		4	1	14		4	1	6	8	2	27	1	4	3	3	10	2	63	
22	KGEYFVAMLEDLLGDGIFNSD	21		1									1				5	1	2				1	1	6	1	5	21	1																		
23	IQIPGYRYLPTKKNRRMWQLD	21															4		2	13	2	4	9																1							2	
24	WKLKRLLNVGSERRL	15																							6	11	5	21																			
25	APLLLPRE	8					1																1							3		11	1	4	1		7	2	20	1		3		9		72	2
26	RPRRLERALRAQGVRGPRY	19							1								3	1	2	12	2	4	6														1		3								
27	YEEGKRIFQLQRELQELASKA	21															4	1	2	12	2	4	10	1								1															
28	YDVDLATRSEQIRFFADALKPAKLRSHVDPMVQEVEEYFAKWGESGT	50		10																																											
29	MLPTMSDCAEEMISRWESSLG	21		1							1						5	1		12			10	2															1								
30	SLPVDLPGTAFARAL	15			4	1		1	1	9	3	1	5	1	7										1	6	5	5				1															
31	LRLGEVPV	8		1												1				9	2	4	5									1				4	10		18		3	1	2	9	3	66	
32	TLRPKGGLPVRVKRR	15		7	4		1		1	6	1		5	1	1		7	1	2	9	1	4	10	3	3	11	5	21	3	2		1				1					3						
33	YLRAHAGEEVDVGEAVYGGVLNLVSSSFFSGDV	33																																				2	25								
34	GAFRPESPFKF	11								5													1		6	11	4	22				1							1								
35	LDEAAVRAIGAAQRDWLLYISRKMSVFAFFPAITKHLFRGR	41																														12	1											1			
36	LKATFLVGPEVSSHFFQGLESEISQGEIYEFTVPMFGKGV	40	1	9		1																								4														1			
37	LDLQGLRRRMA	11			1																															7	5	2	25	1		3	2	9	3		
38	PHFVANRHRFLDWST	15																							6	12	4	17																			
39	DGEGVDVTGSKEIRM	15																													1	17															
40	ERRDEFLRALDEAVK	15		2									1															14	1	4	1	1				1	1		4					2		48	2
41	YAAIQQRVILSHLRRWLDRSAANGGKAEPIRVPCRDMNLETSQTVFAC	50			4																																							1			
42	SWYPRSILEILGKRSILTLLGADHKRLRGLVLRLLGPENLK	41		1		1		1	1	3	3	1	5	1	7														2															2			
43	ASARPLPLRCHDIVPRVLP	19																	2	12	2	4	6							1						1											
44	AFVVDVVRAEVEKRLLPLLAR	21																								11	6	19	1															1		1	
45	GEVPLTMDEIK	11								1		1						1		11	2		8									15	2					2	1		1	1	3	7	3	53	2
46	LSPSYVHA	8								2							8	1	3	13	1	4	10																								
47	HHLVGALP	8		1											1																								5			1		7	2	53	2
48	RVFANVRADAFHLVGHPFGKKLFGDHNLIYLVGEEHKDLRRRIAPNFT	50			4																																										
49	KSMVATSDYGDFHKMAKRNIMLSMLGTSAQKQFRDTRERMISNVLST	50		1																								1		4																	
50	AAAAAGAAVNL	11	2	1			2			1			3				2			4		7	4	2	2		5	6	1	4	3	6	2			5	8		2	1	1	1	3	3	2	63	

[1]: The width of the motif. Each motif describes a pattern of a fixed with as no gaps are allowed in MEME motifs.
