# Supplementary material for: Global identification, structural analysis and expression characterization of cytochrome P450 monooxygenase superfamily in rice
Source: BMC Genomics. 2018 Jan 10;19:35. doi: 10.1186/s12864-017-4425-8 (PMC5764023; doi:10.1186/s12864-017-4425-8)
Supplement: Supplementary file 5 — Multiple sequence alignment of HmCYP51, OsCYP51G1, OsCYP51G3, OsCYP74A5, OsCYP74A4 and AtCYP74A1. Assignment of secondary structure elements is based on HmCYP51 and AtCYP74A1structures. Black frames localize Gotoh’s SRS regions 1–6 identified based on sequence alignment with P450cam, and triangles point to residues constituting the substrate binding site of HmCYP51 (red) and AtCYP74A1 (blue). (PDF 903 kb) [file 12864_2017_4425_MOESM5_ESM.pdf]

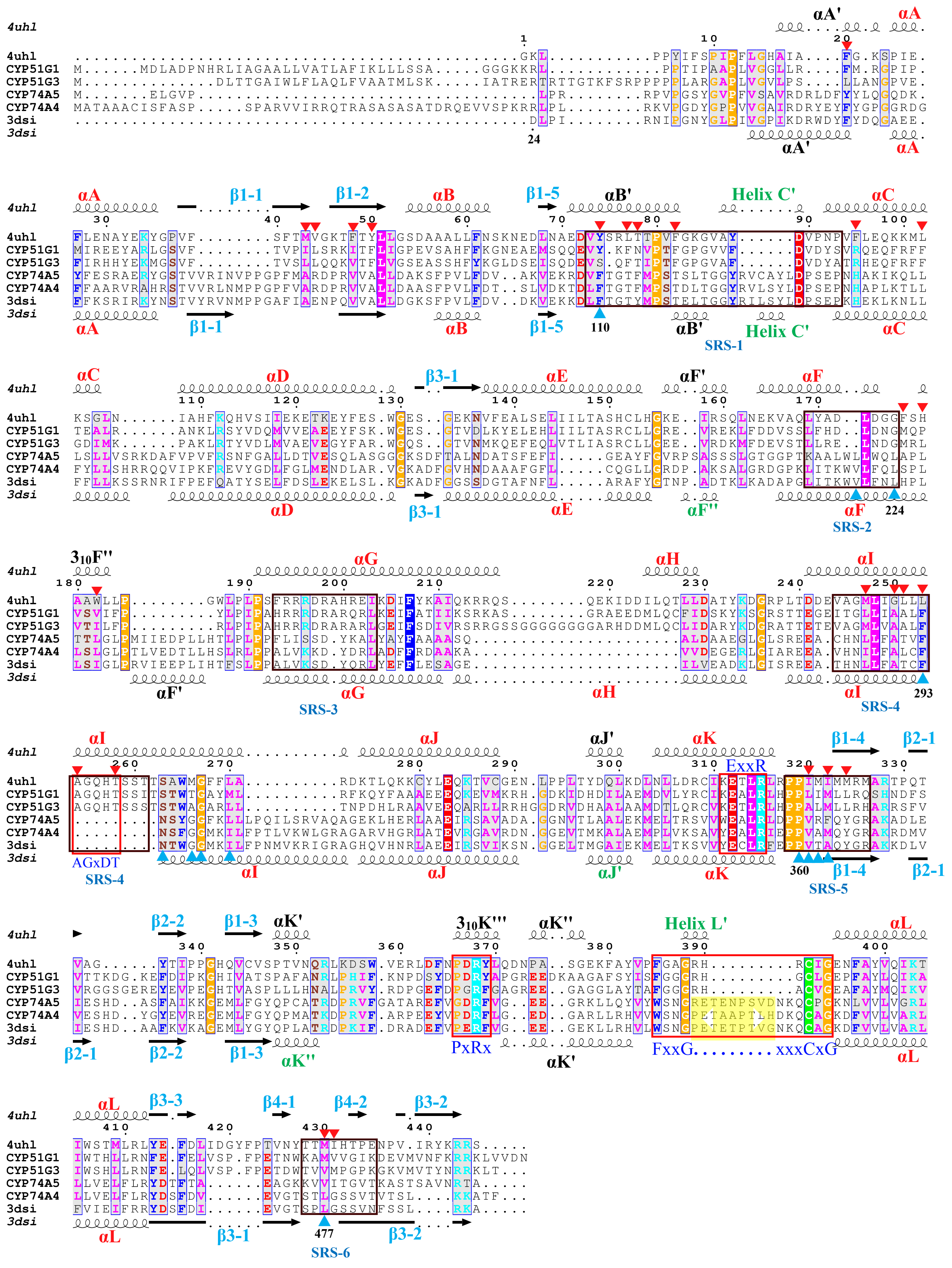

Figure S3. Multiple sequence alignment of HmCYP51, OsCYP51G1, OsCYP51G3, OsCYP74A5, OsCYP74A4 and AtCYP74A1. Assignment of secondary structure elements is based on HmCYP51 and AtCYP74A1structures. Black frames localize Gotoh's SRS regions 1-6 identified based on sequence alignment with P450cam, and triangles point to residues constituting the substrate binding site of HmCYP51 (red) and AtCYP74A1 (blue).
