# Supplementary material for: Global identification, structural analysis and expression characterization of cytochrome P450 monooxygenase superfamily in rice
Source: BMC Genomics. 2018 Jan 10;19:35. doi: 10.1186/s12864-017-4425-8 (PMC5764023; doi:10.1186/s12864-017-4425-8)
Supplement: Supplementary file 15 — List of the number of potential cis-acting elements in 326 OsCYP promoter regions. (PDF 94 kb) [file 12864_2017_4425_MOESM15_ESM.pdf]

Table S9. List of the number of potential cis-acting elements in the upstream 1,000 bp of 326 OsCYP promoter regions.

Condition						Light								light	light	light	light	light	light		endosperm-specific										seed-specific							Salt/Drought	Salt/Drought/Cold		Cold/Drought		Wouding	Salt			Endosperm												
Signal	Auxin	Auxin	Auxin	Auxin	Auxin	CK	GA	GA	GA																					ABA					ABA	ABA	ABA	ABA/JA	ABA/JA	Ethylene				light	JA						SA/Auxin						Light		
Factor						DREB/CBF	Dof/PBF		GA-regulated Myb	GCBP	HMG	HMG	HMG							MADS-BOX transcription factor	MYB	MYB	PBF	PBF	Soybean Embryo Factors	Soybean Embryo Factors	SPBF	SPBF		ZFP				60K protein		ABA/NAC	ABA/NAC	OBRE	AP2/ERF	AP2/ERF	bHLH/MYC	MYB	WRKY		bZIP	bZIP	bZIP	bZIP	bZIP	bZIP	bZIP	bZIP	bZIP	bZIP	bZIP/bHLH	bZIP/ERF	bZIP/MYB/bHLH	Dof	Dof
Elements	AAAACAA motif	ARE-2	CCGTCC-box	GAGACA motif	TGTCAC motif	CAAT-box	P-box	TCCACCT motif	GAACCGAA motif	GCCCC motif	TTCGAAATAT motif	GTAGAA motif	CAAAT motif	GT-1b box	GT2-box	GT3-box	TTTTCC motif	GAAAAA motif	GGTAACTC motif	CArG-box	AACA motif	Box I	prolamin-box	TGTCA/TGACA motif	SEF3 motif	SEF4 motif	SP8 motif	TATCCA motif	AGTCA motif	CACTC motifs	RY/Sph motif	Emlb	Endosperm motif	GAATTTAATTAA motif	B-box	CACGCG motif	CCACG motif	ABRE	DRE/CRT	GCC-box	E-box	MBS	W-box	GT1-box	ACGT motif	Gboxm	GCN4 motif	H-box	C-box	GACATGTC motif	as-1	vicilin box	VSF-1	GAGTCA motif	G-box; ABRE	O2-site	G-box	T/AAAAG motif	AGTA repeat
CYP51G1	1			1	1							1	2	2		1	2	1						2			1		2	1	1							3			1	1	1	1	3		1										4		
CYP51G3	3	1		1			1	1		1		1	2					4					1	1					1	3	1					1		1						2													3		
CYP51G4P										4			1	1				1					2	1	1				1	2						1		1			2		3											1					
CYP51H1										1			4	2				1						1				1		2											1	1	3														1		
CYP51H4	2				1					1			1				2	3			1			3	2					1							1	4	1				4					1						1	1		1		
CYP51H5					1					3			4		1	1		2		1			1	3					1	1	2					1		2			1		7		3		1							1					
CYP51H6	1				2								11				2	4			1			4			4		2								1	1					2																
CYP51H7P	1	1	1				1			1			4				2	2					2		1			1	2	1	1						1	1	1			1	1	2				2									1		
CYP51H8		1			1		1	1					8				1	2			1			4				1	1		1											2			1			1											
CYP51H9	1						1					1	6	5			1	3						2						1	1						1	2					1		2														
CYP71AA2		1			1							1	10	5				2			1			3					1		1												6		1														
CYP71AA3													1	1			4	3			1			1			1			1	1				1			1	4				3		4		1												
CYP71AB1			4							2		1	4			1								1	1					2	1					3		3	3				1		1			3											
CYP71AB2		2								1			9	1			2							3			2		6						1		1	1				1	10		1		1												
CYP71AB3			2	1						1		3	1	1			2	3					1	1					2	1	3						2	2					3	1	2			1											
CYP71AC2			1	2				1		2		1	4								2			2	2				2	1	2						2	3					4	1	3			1											
CYP71AC3P	2						2			2			7	2				4						1	1		1	1	1	4	1		1		1		1	1	1				2		2	1													
CYP71AD1	1				3								7	2			1	2						5					2	1	1				1					1			3		2		1												
CYP71AF1	2			1	1					3		1	7	2	1		2	3			3			6	1		2	2	2	1	1												5	2	1			1										1	
CYP71AK2	1						1						3	2				6			1						2		1	2								1						2	2		1											2	
CYP71C12					2							1	8	1	1			1						5						1	6								2					1	1			1											
CYP71C13P												1	3	2	2	1		4			2		1	3						1	2					1			1			1	3																
CYP71C15												1	2	2			1	1			1			2			4	2	1	1					1								1		1													1	
CYP71C16		1		1	2					1			4	2										2	1					1	2							2	2	1	2	2	3			1													
CYP71C17		1		1	1								2	1			1	3	1		1			1					1	3	1		1		3			1	3		2		4		2						1						1		
CYP71C19P							1			2			6	2			1	5					1	4	1						1				1		1	2	1		2	1	7	3				1											
CYP71C20	1									1			2	2				2			1			2					1	3								1	1				2		2			1											
CYP71C32	1	3	1				2						4	1			2	9					1	3	1			1		1	2										5		3		1			2						1			1		
CYP71C33	1	1		1	1		1			2		1	5	2	1		2	1					1	4	1			2	1	1									2		1		6								1								
CYP71E4		1	1				1			1			2				2							3	1				2	4						1		1	3		1		6		2			1						1					
CYP71E5	2			1	1	1							6	2		1	2	3			1			4					5	1	1					1							8		1			2											
CYP71E6P	1									2			17					2											4	1							1	2	2				5								1								
CYP71K1		1		1			1			2			3	2			1	1						1					1	3	2							1			2		5			1													
CYP71K10	1											1	8	3			2	1					1	6					2									2				1	5		2			1						1					
CYP71K11	1									1			4	1		1							1	1					2	1								1			2		4																
CYP71K12	1						1			1			4			1		4					1	2	1		1			1	1												1																
CYP71K3		1		1						1			4				2				1				2			1	5										1				5	1	1	1								1					
CYP71K4	1									4			3		1	1		2						1					2	1	1				1	2		2					2		2	1													
CYP71K5	1				1					1		1	2				1	1						4					1	1						2		1			1		5		3			1						1			1		
CYP71K6	1	1	1									1	3	1							2			2			1	1	1		1						1	1					3		1														
CYP71K7P													7	3		1	1	2							3				1	1	1							1				2														1			
CYP71K8			1	1						8			1											1	2					1	2					1		4	2				5		2			2			1								
CYP71K9	4			2									8	1									2	6				1	1	2	1				1								7		1														
CYP71P1	2				1					1			6	1			1	4					1	1					2		3	1					1	1					4														2		
CYP71Q1		1					1					2	4	1				3					1	2	1				3		2						1	2			1		6	1	2														
CYP71Q2		3								1			6			1		1						1	2				1	2	5						1	2	6				2		3	1		1											
CYP71R1		1			2					4		1	6	1			1	3						3	1					2		1						4	1	1	2		1		1												1		
CYP71R2P	1									1			3											3			1		3		1								1		2		4																
CYP71S1	1	1		2	5							1	10	1			1	3						9			2		2												2	1	2														1	1	
CYP71S2	2		2	1	1					2			1		1			1						4												1	2	6	5	2		1	3																
CYP71T1												1	2					3						1					1	2								2			1		1		2			1											
CYP71T2		2					1					2	3		1		2							2		1			1	1	1								1			1	4		1			1		1									
CYP71T3	2							1					2	1									1	2			1	2	3	1	2						1	1					2		2														
CYP71T4	1		2							1			2				1	4			1		1						2	3	1										1		2	1	1	1													
CYP71T8										1			4	1			1	2						4	2				1						1						1		4		1			1											
CYP71U3		1											3	1			2	2						3						1						1		1				1	2	1	1										1		1	1	
CYP71V2		3			1								6	1			1							1					1	1			1										4																
CYP71V3		1		1						4		1	3		1		2	1					1				1	1	1	1	2							1	1				5		2										1	1		1	
CYP71V4	1	1		1						1		2	3	1			2	3										3	2		1							1	2		2		2															1	
CYP71V5	1											1	7	1			2	3			1		2	2					6	2	3							1				1	4		2								1						
CYP71W1	2	2		1			1			1			6	3			2	4						4				1	2	1			1					1			1		8	3	2			1											
CYP71W3	2				1							1	2	1			2	5						3						2										1	1		1		2														
CYP71W4					1					2		1	1				1	1						1	1			2									1	3					1		3														
CYP71X10	3				1								8	1				2			2			2						2					1			2					2		2			2											
CYP71X11	2						1	3				2	1	3	1	1	1	4					1				1		2	1	1												2	1	2														
CYP71X12	1		1	1	2					1		2	6				1	3			1			5			1		1	4											2	2	2		2										1			1	
CYP71X13P			2	1	2									3			1	2					1	5	1		2		5	1	1						1	2					5		2	1											1		
CYP71X14												1	4	1	1		1	2							1		2		2	1								1	1		2		8	1	1														
